# Traditional uses, phytochemical, pharmacology, quality control and modern applications of two important Chinese medicines from *Rosa laevigata* Michx.: A review

**DOI:** 10.3389/fphar.2022.1012265

**Published:** 2022-10-06

**Authors:** Xiao-Xiao Quan, Yuan-Yuan Huang, Lu Chen, Jing-Quan Yuan

**Affiliations:** ^1^ Scientific Experimental Center of Guangxi University of Chinese Medicine, Nanning, China; ^2^ Guangxi Botanical Garden of Medicinal Plants, Nanning, China

**Keywords:** *Rosa laevigata* Michx., Fructus *R. laevigata*, Radix *R. laevigata*, traditional medicine, phytochemistry, pharmacology, quality control

## Abstract

*Rosa laevigata* Michx. is an ethnic medicine that have strong biological activities used in the traditional medicine system for the treatment of diabetes, nephropathy, myocardial damage, oxidative damage, liver damage and so on. Currently, The Chinese herb *R. laevigata* Michx. can be divided into two important medicines: Fructus *R. laevigata* and Radix *R. laevigata*, from which approximately 148 chemical components have been isolated, including flavonoids, lignans, polyphenols, steroids, triterpenoids, tannins as well as other components. Pharmacological studies have already confirmed that both of these herbs have antioxidant, anti-inflammatory, antiviral and anti-tumor activities, as well as renal protective, immunomodulatory, lipid-lowering, cardiovascular protective, bacteriostatic, and other pharmacological effects. Toxicological tests and quality control studies revealed the safety and nontoxicity of *R. laevigata* Michx. Therefore, this paper systematically summarizes the traditional uses, botanical, phytochemical, and pharmacology as well as the quality control and toxicology of Fructus and Radix, which in order to provide a comprehensive reference for its continued research.

## 1 Introduction

*Rosa laevigata* Michx. belongs to the Rosaceae family and is a widely used plant in China. Its different parts are used as herbs in Chinese medicine, its flowers, leaves and stems have different applications, but the main parts used are its fruits and roots, which are two herbs with so much important applications in Chinese medicine. R. *laevigata* is divided into many types of herbs according to its different medicinal parts, and among these the two most used are: Fructus *R. laevigata* and Radix *R. laevigata*.

One of these herbs is Fructus *R. laevigata* (JinYingZi) which is the dried ripe fruit of the Rosaceae plant that is often called Prickly Elm. It was first recorded in the *Shu Ben Cao* (during 935-960 AD) written by Han Baosheng and now included in the *Chinese Pharmacopoeia*. It belongs to the kidney, bladder and large intestine meridian. It has the effects of consolidating the essence and shrinking of urine, consolidating the collapse and stopping belt as well as astringent intestines and stopping diarrhea. It is mainly used for spermatorrhea, frequent enuresis, metrorrhagia and diarrhea (National Pharmacopoeia Committee, 2020).

Another one of these herbs is Radix *R. laevigata* (JinYingGen) which is also known as Tuogudan. It was first recorded in the *Ri Hua Zi Ben Cao* and it is flat and non-toxic. It is used to strengthen the essence and astringe the intestines as well as the treatment of spermatorrhea, enuresis, dysentery, diarrhea, metrorrhagia, uterine prolapse, hemorrhoids, scalding and so on (Tan et al., 2010).

Studies have shown that the different parts of the herbs lead to small differences in their efficacy. The extracts of Radix *R. laevigata* can treat age-related urinary incontinence by causing changes in bladder forcing muscles and bladder capacity during the filling phase (Tianjin No.3 Hospital, 2004). Fructus *R. laevigata* can treat the symptoms of kidney deficiency, its administration can improve kidney function as well as improve any difficulties in urination and edema in men and women. It also can reduce the frequent night urination. In addition, fructus *R. laevigata* can improve the function of the gastrointestinal tract, promoting the peristalsis of the intestines, increasing food digestion and reducing the accumulation of harmful substances in the intestinal tract. It also plays a role in elimination of the stool and combats diarrhea and it can reduce the occurrence of gastrointestinal diseases. In terms of chemical composition, both Fructus and Radix are similar. Triterpenoids are almost the same, while the flavonoids, tannins and other components are somewhat different. The corresponding pharmacological activities are also slightly different, with anti-inflammatory, antioxidant, anti-tumor and immunological activities allotted to both medicinal herbs, while other pharmacological activities are different. Fructus *R. laevigata* and Radix *R. laevigata* are also different in their applications and, each has its own focus. The Ministry of Health of China has rated Fructus *R. laevigata* as a new food resource, which has been developed into a third generation of wild fruit food. Therefore, Fructus *R. laevigata* is widely used in food ingredients, such as its addition when developing fruit juices, fruit wines and yogurt (Li et al., 2022). In addition, a brown pigment for use as a food additive can also be extracted from Fructus. It is also used as raw material for Chinese medicine to treat pelvic inflammation and diabetic cataract, while Radix *R. laevigata* is used as raw material in Sanjin tablets and gynecological Qianjin tablets to treat gynecological infections. In addition, and it is also used as the main raw material of Guangdong herbal tea.

At present, there are an ever-growing list of reports on Fructus *R. laevigata* and Radix *R. laevigata.* Therefore, this review respectively summarizes the chemical composition, pharmacological effects, and quality control as well as the extraction, separation and processing of Fructus *R. laevigata* and Radix *R. laevigata* in order to provide a basis for subsequent research on these two important Chinese medicines.

## 2 Materials and methods

The available information on *Rosa laevigata* Michx. was collected from scientific databases published from 1989 up to 2021. Information on *R. laevigata* was obtained from published sources, including monographs on medicinal plants, ancient and modern recorded classics, the Chinese Pharmacopoeia and electronic databases, such as Web of Science, PubMed, CNKI, Wanfang DATA, Google Scholar, Baidu Scholar and Flora of China (FOR). The search terms used for this review included “*Rosa laevigata* Michx.”, “Fructus *Rosa laevigata*”, “The root of *Rosa laevigata*”, “Radix Rosae laevigate” and “Rosaceae” all of which are accepted names and synonyms, “botanical characterization”, “compounds”, “traditional uses”, “pharmacology”, “toxicology”, “quality standard”, “extraction and purification” and “applications”. No language restrictions were applied during the search.

## 3 Botanical description and geographical distribution

### 3.1 Botanical description

Fructus *Rosa laevigata* Michx. is the dried ripe fruit of Rosaceae plant family. Flora of China describes it as an evergreen climbing shrub that is approximately 5 m tall and the branchlets are stout with a glabrous stem and having thin stripes. The leaflets are leathery, usually three but more rarely can be five and are 5–10 cm long with petioles. The leaves are elliptical and ovate, obovate or lanceolate-ovate, measuring approximately 2–6 cm long and 1.2–3.5 cm wide. The apex of each leaf is acute or rounded with a sparsely caudate-acuminate and the margin is sharply serrated. The coloration is bright green when viewed from above and yellow-green from below with glabrous. The petiolules and leaf rachis have prickles and glandular hairs. The stipules are free or the base are united with petiole presenting in a lance shape and the margins are finely toothed with glandular tips and are caducous. The flowers are solitary in the leaf axils and are 5–7 cm in diameter, whereas the pedicel are 1.8–2.5 cm long and can occasionally be up to 3 cm. Additionally, the pedicels and calyx tubes are densely glandular and hairy, and these become acicular as the fruit grows. The spals are ovate-lanceolate with a leaf-like apex and the margin are pinnately lobed or entire which often with spiny and glandular hairs. The inner surface is densely pilose and it is slightly shorter than the petals which are white and are broadly obovate. The apex is retuse. There are numerous stamens and carpels, and styles are free and hairy and much shorter than stamens. The purple-brown fruit is pear-shaped, obovate or sparsely sub-globose. The outer layer is densely covered with prickly hairs and the pedicel of fruit is approximately 3 cm long with persistent, sepals. The flowers can be collected in early summer (April to June) and the seeds are collected in July to November (Editorial Board of Flora of China, Chinese Academy of Sciences, 2004).

### 3.2 Geographical distribution

Roseceae are widely distributed in the temperate and subtropical regions of the northern hemisphere, and their centres are localized in Central and South-West Asia. Asia has the largest number of wild rose species and the longest history of their existence. China is the modern distribution centre of Rosaceae plants, and *R. laevigata* is mainly found in south-western, southern, central and eastern China. They grow in area such as Shaanxi, Anhui, Jiangxi, Jiangsu, Zhejiang, Hubei, Hunan, Guangdong, Guangxi, Taiwan, Fujian, Sichuan, Yunnan and Guizhou. This plant is usually found in sunny mountain fields, field margins and streamside, and it is generally found in the mountains at altitudes of 200–1600 m (Liu X. G. et al., 2013). *R. laevigata* is also found in Tibet, mainly in the sunny mountains of southern Tibet at altitudes of 1500–3500 m. *R. laevigata* produced in this region is influenced by the special climatic conditions and is of better medicinal quality than the mainland varieties (Liu et al., 2010a).

## 4 Traditional uses

*Rosa laevigata* Michx. was first published in the *Shu Ben Cao*(Han, Five Dynasties) written by Han Baosheng during 935-960 AD (Later Shu of the Five Dynasties) (Han et al., 935-960 AD). Its description in this auspicious work was: “*R. laevigata* is found everywhere. The flowers are white. The seeds resemble quince but are small and yellow with spines. It is often used in Chinese traditional medicine.” *Dream Pool Essays* (Shen et al., the end of the 11th century)*,* which was written at the end of the 11th century, mentioned the following: “*R. laevigata*, stop the ejaculation, to take its warm and astringent, the world with gold poppy, to be its red ripe, take the juice and boil the paste to use, a big mistake, red is the taste of sweet, boil the paste is all broken astringent taste, all lose the nature. Nowadays we should pick the half yellow leaves, and these should be dried and pounded before use.” It is also written in the *Compendium* (Li et al., the middle of the Ming Dynasty) (written in the middle of the Ming Dynasty, during the Jiajing period) that “If one takes *R. laevigata* for no reason, in order to obtain a quick desire, one should not; if one takes them if one’s essence is not solid, there is no blame.” Written by Huangfu Zhong between 1,368 and 1644 AD, the *Guide to Famous Doctors* (Huangfu et al., 1368-1644 AD)states that “*R. laevigata* is useful for treating dream loss and spermatorrhea”. There are also many local stories that include the effects of *R. laevigata*. For example, the *Min Dong Materia Medica* (The Editorial Committee of Eastern Fujian Materia Medica, 1962) mentions that *R. laevigata* can be used to treat hypospermia and spermatorrhea in men and leucorrhoea in women, as well as to treat pubic erections. The *Quanzhou Materia Medica* (Quanzhou Municipal Science and Technology Commission et al., 1961)also records that *R. laevigata* can cure frequent urination, polyuria, incontinence of urine, and also the chronic deficiencies diarrhea and dysentery.

Radix *R. laevigata* has also been included in many local chronicles: the Lingnan Herb Journal (Guangdong Institute of Traditional Chinese Medicine et al., 1961) records that it can cure spermatorrhea and treat stomach pains. The Hunan Medicinal Journal (Hunan Institute of Traditional Chinese Medicine, 2017) mentions that the roots of *R. laevigata* is a remedy for children’s enuresis, for diarrhoea, and as a treatment for bruises. It is also described in the *Jiangxi Folk Herbal Recipes* (Gong et al., 1963) as a remedy for women’s leaks and for fire and soup injuries, as well as for recurrent fires in the lower limbs. In the *Min Dong Materia Medica* (Editorial Committee of Eastern Fujian Materia Medica., 1962), the roots of *R. laevigata* are used to treat lumbar spinal pain and rheumatic joint pain. The Chinese Medicine in Guangdong (Guangdong Provincial Department of Health, 1959) documents its effectiveness in treating a prolapsed uterus. A summary of the prescription names, sources of prescriptions and formulas for *R. laevigata* and its roots is given in Supplementary Table S1.

This shows that the Chinese patent medicines which have *R. laevigata* as one of the main ingredients include Shuiluerwei pill, Zhuangyaojianshen pills, Zhuangyaojianshen tablets, ancient Chinese health essences, ancient Chinese health tablets and so on. Chinese patent medicines with the roots of *R. laevigata* as the main ingredient include Gynecological Qian Jin Tablets, San Jin Tablets, Gynecological Qian Jin Capsules, Jin Ji Capsules and Guangdong herbal tea. The sales of these products support the Chinese economy to approximately US $5 billion.

## 5 Modern application

The four representative prescription formulations that existed in traditional applications have been optimized with modern technology to produce the widely used proprietary Chinese mediciens (pCms) of today, namely Guangdong Herbal Tea Granules, Gynecology Qianjin Tablets, Sanjin Tablets and Jin Ji Capsules.

Guangdong Herbal Tea Granules (State Drug Certification Z44020615) are granules, which are manufactured by the Guangzhou Wanglaoji Pharmaceutical Co. They are used for clearing away damp-heat, relieving summer heat and producing body fluids containing waste products harmful to the person. They are also used for treating colds of the four seasons, fever and sore throat as well as damp-heat stagnation, dry mouth and redness of the urine.

Gynecology Qianjin Tablets (State Drug Certification Z43020027) are manufactured by the Zhuzhou Qianjin Pharmaceutical Co. These are used for clearing away heat, tonifying Qi and resolving blood stasis. For heat and stagnation caused by these symptoms that originate in the abdomen, manifesting as profuse, yellowish and thick, foul-smelling discharge, abdominal pain, lumbosacral pain and fatigue. These are also useful for treating chronic pelvic inflammatory disease, endometritis and chronic cervicitis with the above symptoms. As a Qianjin Pharmaceutical’s flagship product,“Gynecological Qianjin Tablets” still accounts for a disproportionately large share of the main business sales revenue, accounting for more than 80% of its sales revenues, and it is the “only product” of note from this company.

Sanjin Tablets (State Drug Certification Z45021645) are manufactured by the Guilin Sanjin Pharmaceutical Co. They are used for clearing away heat and detoxifying harmful toxins in the body, clearing away damp-heat and promoting drenching, and benefiting the kidneys. They are also used for treating pyorrhea and redness in the urine, dripping and astringent pain, frequent urination due to humidity and heat in the lower jiao as well as acute and chronic pyelonephritis, cystitis and urinary tract infections associated with the above symptoms. By the end of 2015, “Sanjin Tablets” had established long-term stable customer relationships with more than 500 distributors and more than 2,300 hospitals of Grade IIA or above as well as more than 21,000 pharmacies. They occupied the herbal markets in more than 30 provinces and cities across the country, with leading levels of quality, technology, efficacy and other indicators always being assured. This product has proven to have outstanding qualities and it has remained on the market for more than 30 years.

Jin Ji Capsules (State Drug Certification Z45020293) are manufactured by the Guangxi Lingfeng Pharmaceutical Co. Its functions include clearing away heat and detoxifying harmful toxins, invigorating the spleen and clearing away damp-heat, promoting circulation and activating circulation of the blood. In addition, for adnexitis, endometritis and pelvic inflammatory disease caused by damp-heat infiltration.

These proprietary Chinese medicines are mainly made of Radix *R. laevigata*, are household names in China and have a pharmaceutical output value of several billion RMB in the country.

## 6 Phytochemistry

**FIGURE 1:**
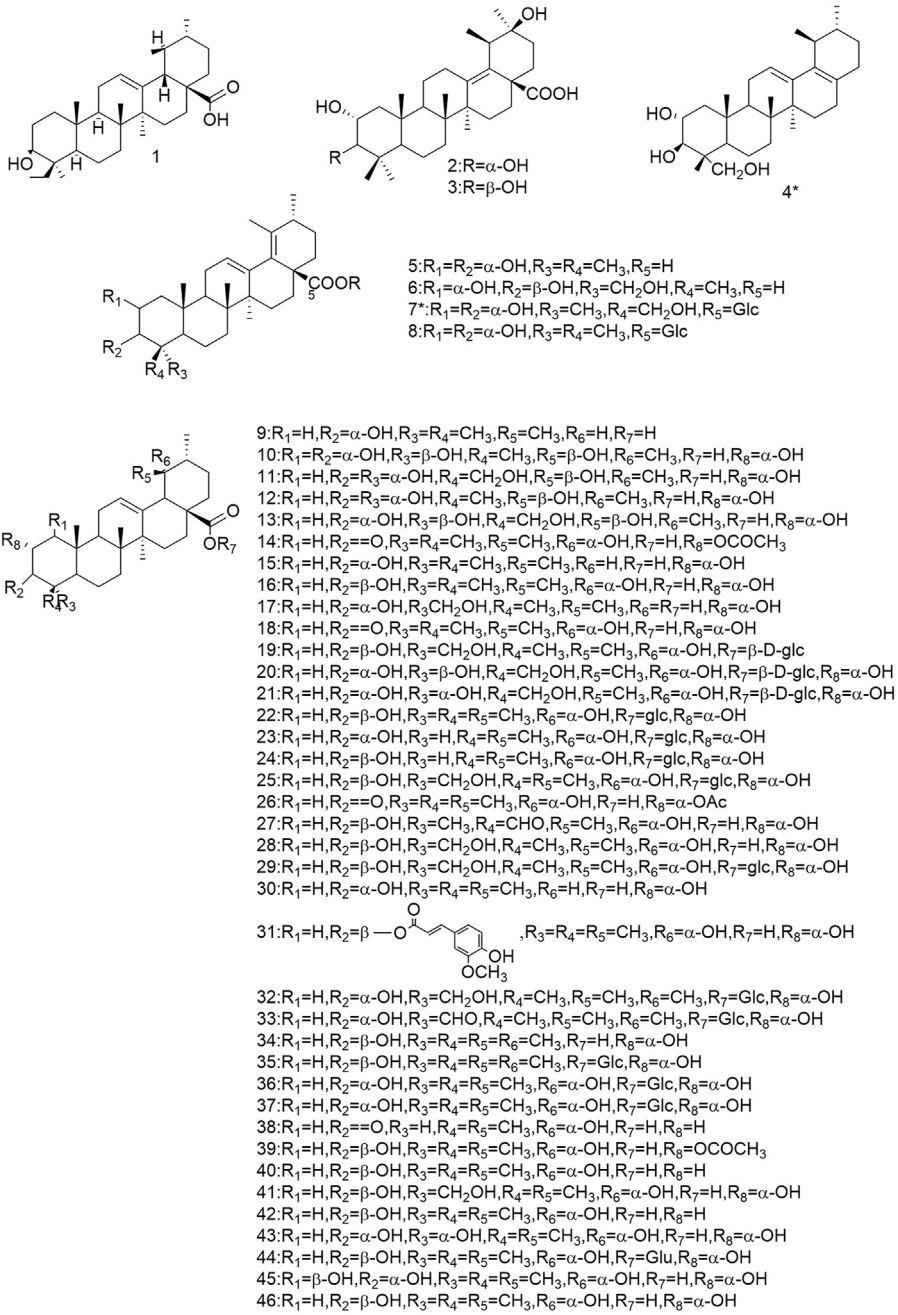
Diagrammatic representation of the structural formulas of triterpenoids of compounds found in *R. laevigata* Michx.

**FIGURE 2:**
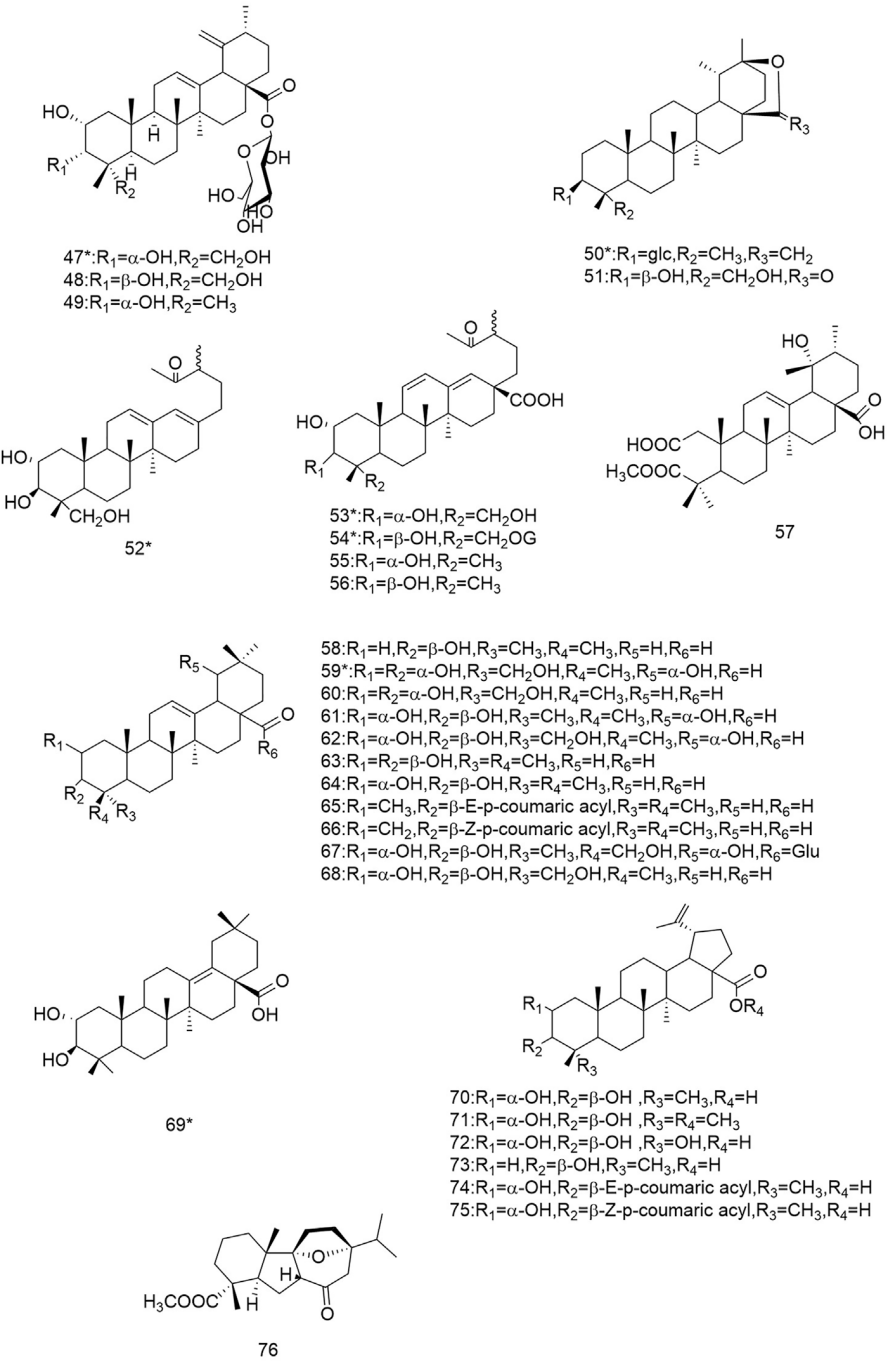
Diagrammatic representation of the structural formulas of triterpenoids of compounds found in *R. laevigata* Michx.

**FIGURE 3:**
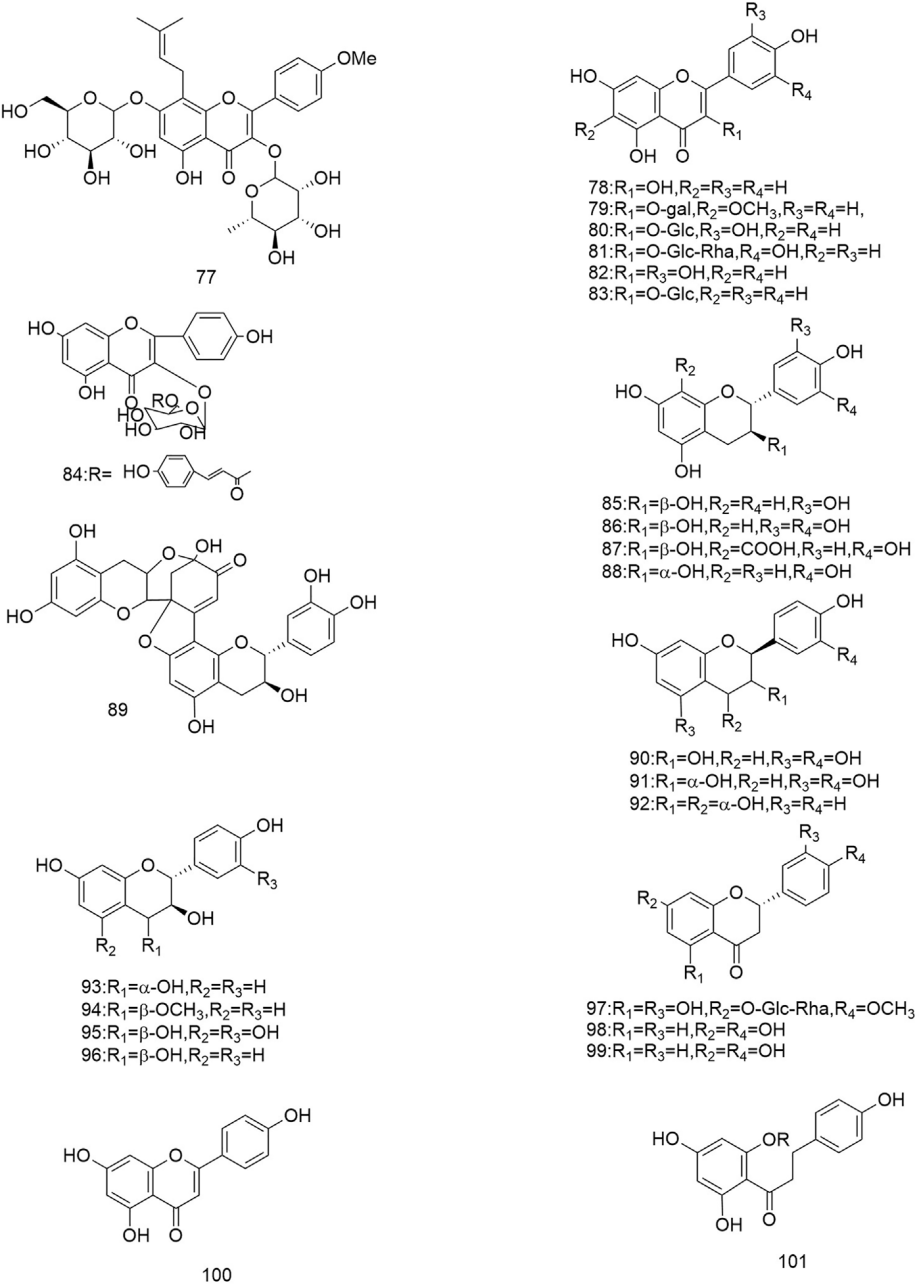
Diagrammatic representation of the structural formulas of flavonoids of compounds found in *R. laevigata* Michx.

**FIGURE 4:**
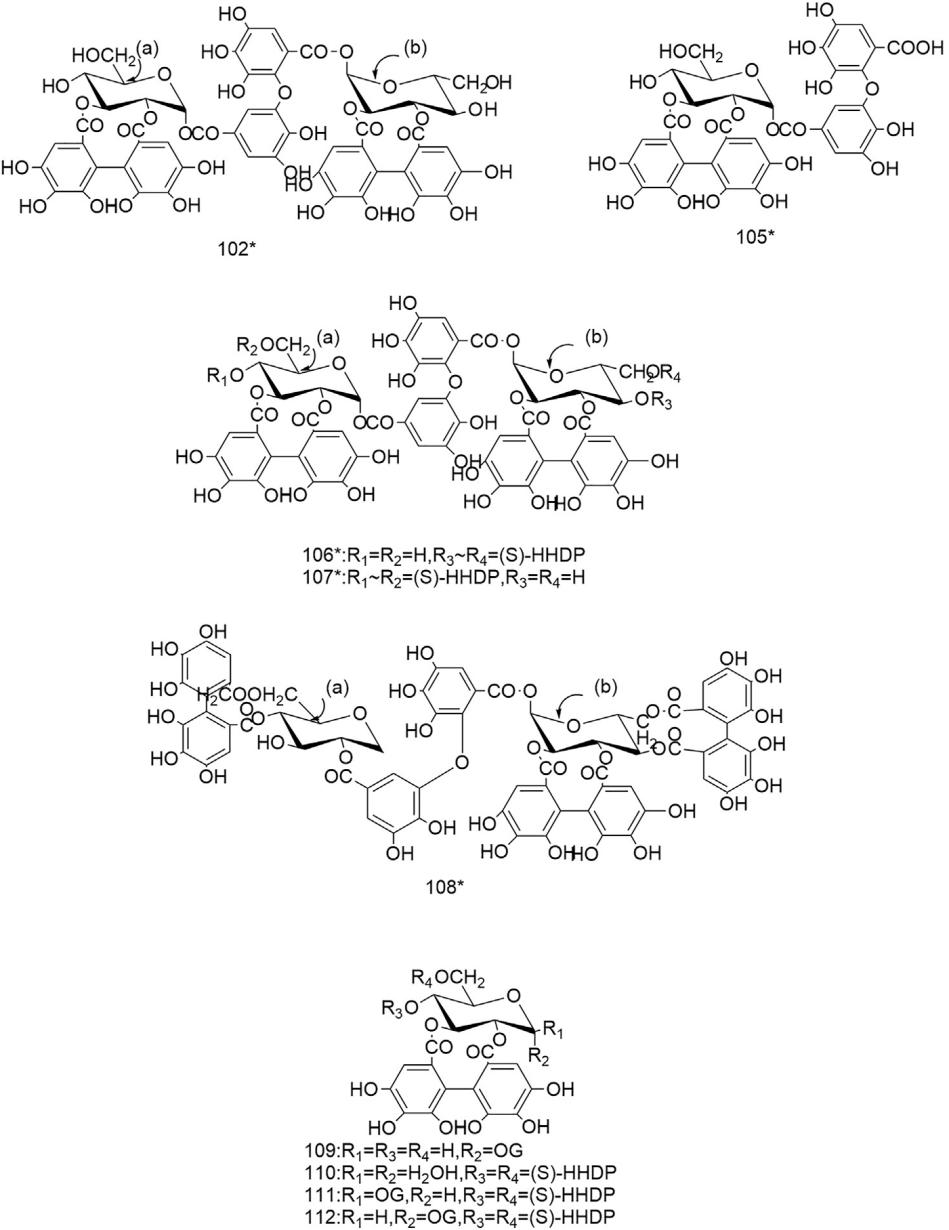
Diagrammatic representation of the structural formulas of tannins of compounds found in *R. laevigata* Michx.

**FIGURE 5:**
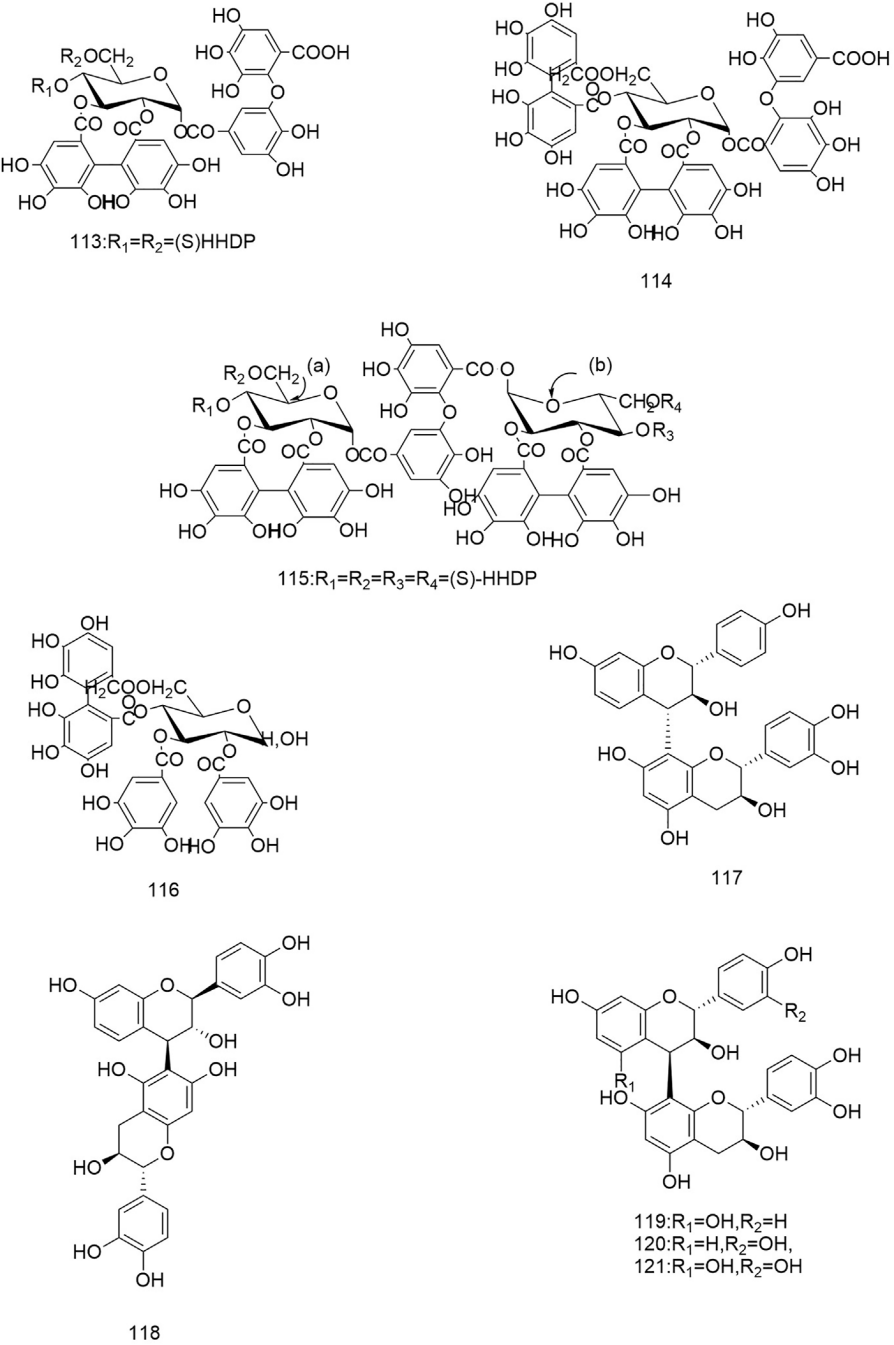
Diagrammatic representation of the structural formulas of tannins of compounds found in *R. laevigata* Michx.

**FIGURE 6:**
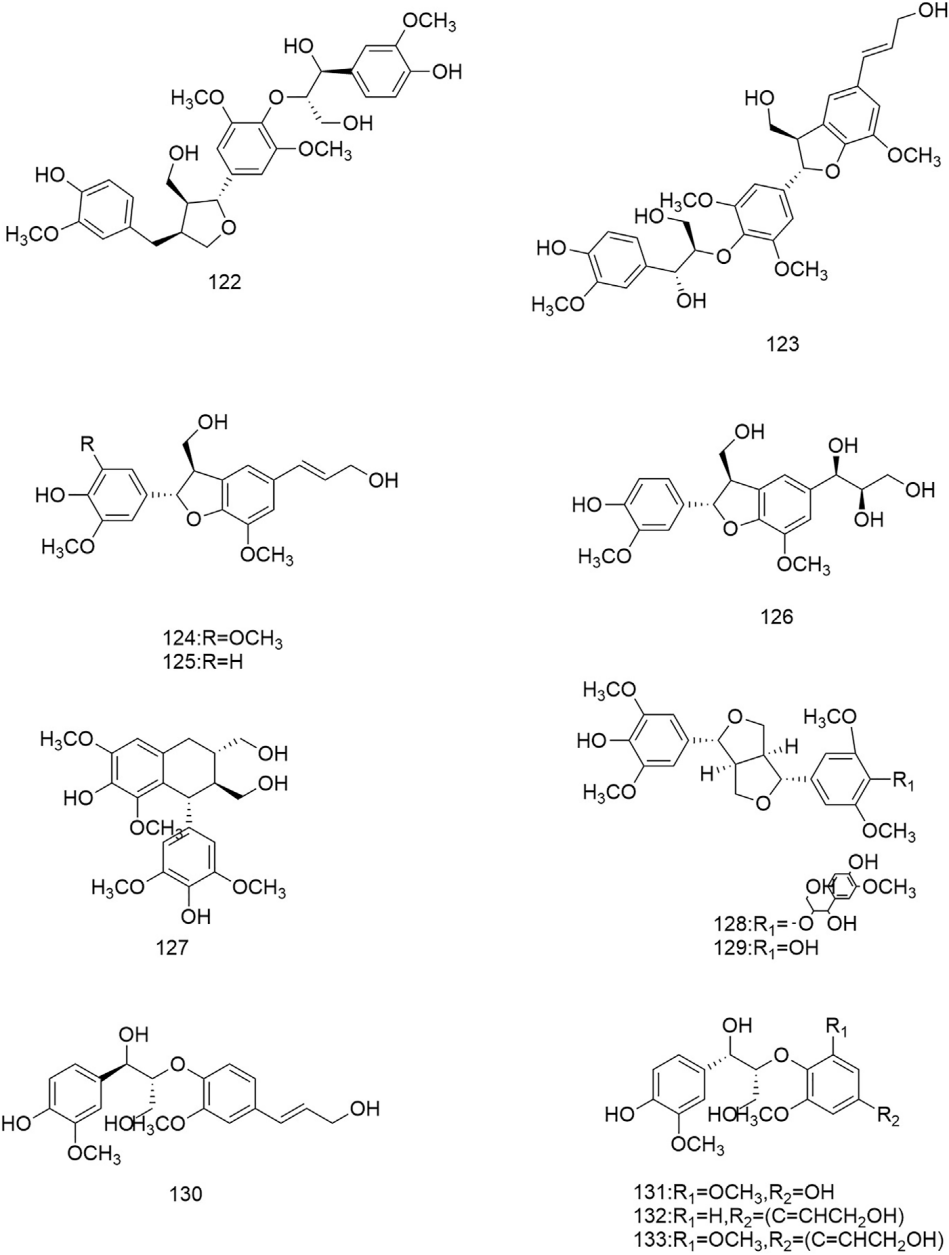
Diagrammatic representation of the structural formulas of others of compounds found in *R. laevigata* Michx.

**FIGURE 7:**
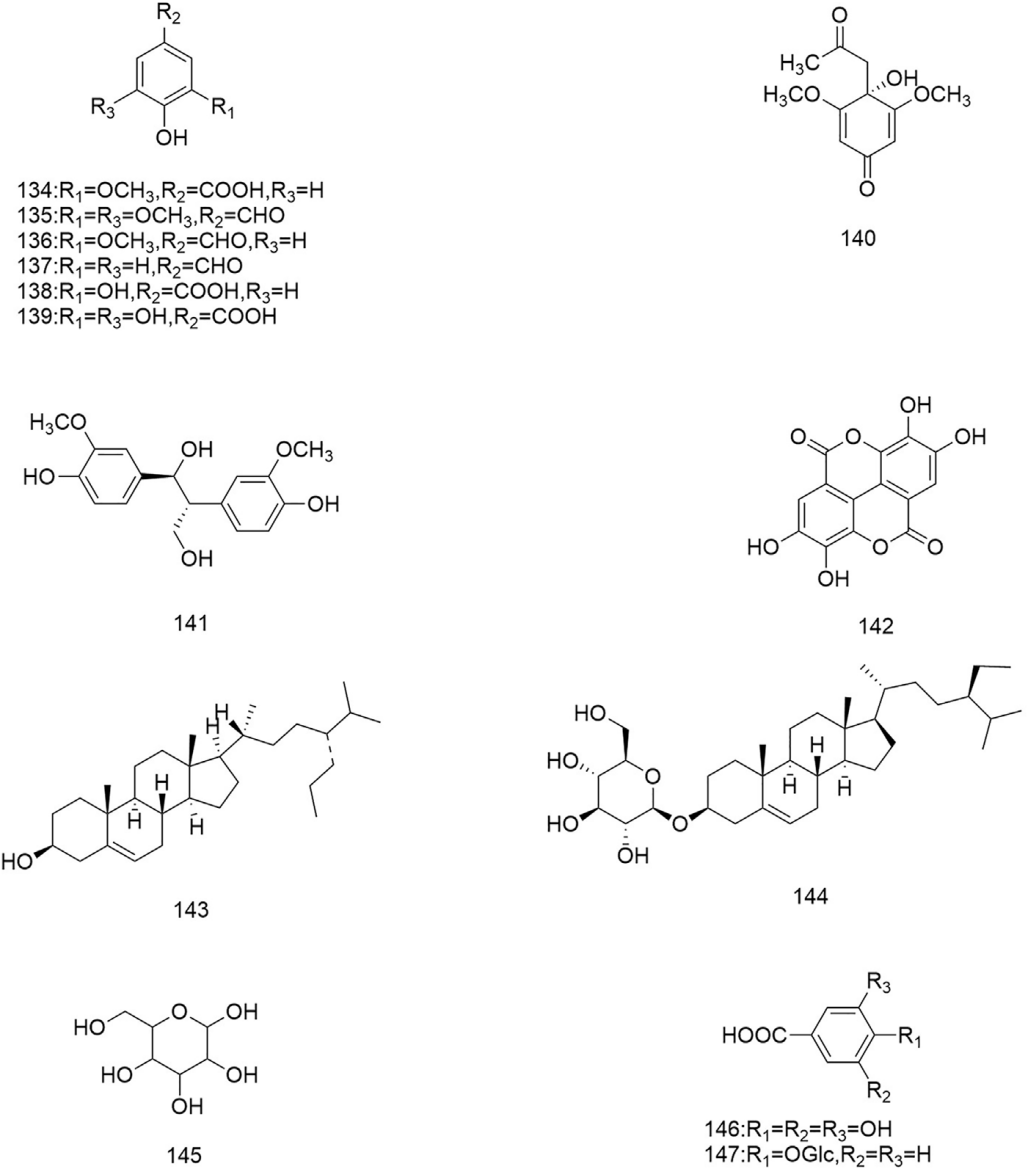
Diagrammatic representation of the structural formulas of others of compounds found in *R. laevigata Michx*.

So far 148 components have been isolated from *Rosa laevigata* Michx. of which (82 and 68 compounds are from Fructus *R. laevigata* and Radix *R. laevigata* and 15 and six are new, respectively. In addition, 12 identical chemical components are present in both herbs) and these include triterpenes, flavonoids, tannins, lignans, phenolics and other compounds. The chemical constituents that have been identified are listed in Table 1 and their corresponding structures are diagrammatically shown in Figures 1–7.

**TABLE 1:** The composition of *R. Laevigata* Michx.

NO.	Chemical ingredient	Source of ingredients	Ref
Triterpenoids			
Ursane			
1	Ursolic acid	Fructus,Radix	Li and Wei, 1997, Gao et al., 2010
2	2*α*, 3*α*, 20*β*-trihydroxyursane-13 (18)-en-28-oic acid	Radix	Li et al. (2017)
3	2*α*, 3*β*, 20*β*-trihydroxyursane-13 (18)-en-28-oic acid	Radix	Li et al. (2017)
4	2*ɑ*,3*β*,23-trihydroxy-12,17-dien-28-norursane*	Fructus	Li and Wei, (1997)
5	2*α*,3*α*-dihydroxy-12,18-diene-28-carboxylic acid	Radix	Liu et al. (2018)
6	2*α*,3*β*,23-trihydroxy-urs-12,18-dien-28-oic acid	Radix	Gao et al. (2010)
7	2*ɑ*,3*ɑ*,24-trihydroxy-urs-12,18-dien-28-oic acid-*β*-d-glucopyranosyl ester*	Radix	Yuan et al. (2008)
8	Rubuside B	Radix	Dai et al. (2016)
9	2*ɑ*-hydroxy-urs-12-en-28-oic acid	Fructus	Bi et al. (2008)
10	1*α*,2*α*,3*β*,19*α*-tetrahydroxyurs-12-en-28-oic acid	Fructus,Radix	Liu et al. (2013b), Gao et al., 2010
11	2*ɑ*,3*ɑ*,19*ɑ*,23-tetrahydroxy-urs-12-en-28-oic acid	Fructus	Liu et al. (2013a)
12	2*ɑ*,3*ɑ*,19*ɑ*-trihydroxy-urs-12-en-28-oic acid	Fructus	Liu et al. (2013b)
13	2*ɑ*,3*β*,19*ɑ*,23-tetrahydroxy-urs-12-en-28-oic acid	Fructus	Liu et al. (2013a)
14	(2*α*,19*α*)-Methyl-2-acetoxy-19-hydroxy-3-carbonyl-ursane-12-en-28-carboxylic acid	Radix	Liu et al. (2018)
15	2*α*,3*α*-dihydroxy-urs-12-en-28-oic acid	Radix	Li et al. (2017)
16	2*α*,3*β*,19*α*-trihydroxy-urs-12-en-28-oic acid	Radix	Gao et al. (2010)
17	2*α*,3*α*,23-trihydroxy--urs-12-en-28-oic acid	Radix	Gao et al. (2010)
18	2*α*,19*α*-dihydroxy-3-oxo-urs-12-en-28-oic acid	Radix	Gao et al. (2010)
19	*β*-d-Glucopyranosyl-3*β*,19*ɑ*-dihydroxy-2-oxo-urs-12-en-28-oate	Radix	Ding et al. (2017)
20	2*ɑ*,3*β*,19*ɑ*,23-tetrahydroxy-urs-12-en-28-oic acid-*β*-d-glucopyranosyl ester	Fructus, Radix	Wang et al., 2001, Wang X. J. et al. (2016)
21	2*ɑ*,3*ɑ*,19*ɑ*,23-tetrahydroxy-urs-12-en-28-oic acid-3-*O*-*β*-d-glucopyranosyl ester	Fructus, Radix	Bi et al., 2008, Ding et al., 2017
22	2*ɑ*,3*β*,19*ɑ*-trihydroxy-urs-12-en-28-oic acid-*β*-d-glucopyranosyl ester	Radix	Wang Y. M. et al. (2016)
23	2*ɑ*,3*ɑ*,19*ɑ*-trihydroxy-urs-12-en-28-oic acid-*β*-d-glucopyranosyl ester	Radix	Wang X. J. et al. (2016)
24	2*α*,3*β*,19*α*-trihydroxy-urs-12-en-28-oic acid-*β*-d-glucopyranosyl ester	Radix	Wang Y. M. et al. (2016)
25	2*α*,3*β*,19*α*,23-tetrahydroxy-urs-12-en-28-oic acid-*β*-d-glucopyranosyl ester	Radix	Wang X. J. et al. (2016)
26	(2*α*,19*α*)-methyl-2-acetyloxy-19-hydroxyl-3-oxo-urs-12-en-28-carboxylate	Radix	Dai et al. (2016)
27	2*α*,3*β*,19*α*-trihydroxy-24-oxo-urs-12-en-oic acid	Radix	Dai et al. (2016)
28	19*ɑ*-hydroxy-asiatic acid	Fructus, Radix	Ye et al., 1993, Gao et al., 2010
29	19*ɑ*-hydroxy-asiatic acid-28-*O*-*β*-d-glucopyrannoside	Fructus, Radix	Ye et al., 1993, Gao et al., 2010
30	Euscaphic acid	Fructus,Radix	Li and Wei, 1997, Zeng et al., 2011
31	19*α*-OH-3*β*-E-feruloyl corosolic acid*	Radix	Li et al. (2017)
32	Nigaichigoside F2	Radix	Zeng et al. (2011)
33	Rubuside J	Radix	Zeng et al. (2011)
34	Tomentic acid	Radix	Zeng et al. (2011)
35	Rosamutin	Radix	Zeng et al. (2011)
36	Rosamultin	Radix	Zeng et al. (2011)
37	Kajiichigoside F1	Radix	Zeng et al. (2011)
38	Pomonoic acid	Radix	Liu et al. (2018)
39	2-acetic acid Potentilla acid	Radix	Liu et al. (2018)
40	Pomic acid	Radix	Liu et al. (2018)
41	23-hydroxy-tormentic acid	Radix	Li et al. (2017)
42	Pomolic acid	Radix	Li et al. (2017)
43	Myrianthic acid	Radix	Gao et al. (2010)
44	Tormentic acid-28-*O*-*β*-d-glucopyranoside	Radix	Gao et al. (2010)
45	1*β*-Hydroxyrosalic acid	Radix	Dai et al. (2016)
46	Potentilla acid	Radix	Dai et al. (2016)
47	2*ɑ*,3*ɑ*,23-trihydroxy-urs-12.19 (29)-dien-28-oic acid-*β*-d-glucopyranosyl ester*	Radix	Yuan et al. (2008)
48	Quadranoside Ⅷ	Radix	Dai et al. (2016)
49	Sanguisorbin	Radix	Dai et al. (2016)
50	3*β*-[(R-l-arabinopyranosyl)oxy]-20*β*-hydroxyursan-28-oic acidδ-lactone*	Fructus	Li and Wei, (1997)
51	3*β*,23*ɑ*-dihydroxy-ursan-28-oic acid-δ-lactone	Fructus	Li and Wei, (1997)
52	2*ɑ*,3*β*,23-trihydroxy-19-oxo-18,19-seco-12,17-dien-28-norursane*	Fructus	Li and Wei, (1997)
53	2*ɑ*,3*ɑ*,23-trihydroxy-19-oxo-18,19-seco-urs-11.13 (18)-dien-28-oic acid*	Fructus	Li and Wei, (1997)
54	18,19-split ring-2*α*,3*β*,23*α*-trihydroxy-19-carbonyl-ursane-11.13 (18)-diene-28-carboxylic acid*	Radix	Liu et al. (2018)
55	18,19-seco-2*α*,3*α*-dihydroxy-19-oxo-urs-11.13 (18) -dien-28-oic acid	Radix	Dai et al. (2016)
56	Swinhoeic acid	Radix	Dai et al. (2016)
57	Claric acid-3-methyl ester	Radix	Liu et al. (2018)
Oleanane			
58	Oleanolic acid	Fructus, Radix	Li and Wei, 1997, Gao et al., 2010
59	2*ɑ*,3*ɑ*,19*ɑ*,23-tetrahydroxyolean-12-en-28-oic acid*	Fructus	Wu et al. (2009)
60	2*α*,3*α*,23-trihydroxy-olean-12-en-28-oic acid	Fructus,Radix	Wu et al., 2009, Gao et al., 2010
61	2*α*,3*β*,19*α*-trihydroxy-olean-12-en-28-oic acid	Fructus,Radix	Yoshida et al., 1989, Gao et al., 2010
62	2*α*, 3*β*, 19*α*, 23-tetrahydroxyolean-12-en-28-oic acid	Radix	Li et al. (2017)
63	2*α*,3*α*-dihydroxy-olean-12-en-28-oic acid	Radix	Gao et al. (2010)
64	Maslinic acid	Fructus	Li and Wei, 1997, Gao et al., 2010
65	3-*O*-trans-p-coumarinyl-Maslinic acid	Fructus	Liu et al. (2013a)
66	3-*O*-cis-p-coumarinyl-Maslinic acid	Fructus	Liu et al. (2013b)
67	Sericoside	Radix	Ding et al. (2017)
68	Arjunolic acid	Radix	Dai et al. (2016)
69	2*ɑ*,3*β*-dihydroxy-olean-13 (18)-en-28-oic acid*	Fructus	Wu et al. (2009)
Lupane			
70	2*α*,3*β*-dihydroxy-lup-20 (29) -en-28- oic acid	Fructus, Radix	Liu et al. (2013a), Gao et al., 2010
71	2*ɑ*,3*β*-dihydroxy-lup-20-en-28-methyl ester	Fructus	Liu et al. (2013b)
72	2*α*,3*β*,23-trihydroxy-lup-20 (29) -en-28-oic acid	Radix	Gao et al. (2010)
73	Betulinic acid	Fructus, Radix	Liu et al. (2013a), Zeng et al., 2011
74	3-*O*-trans-p-coumaric acid	Fructus	Liu et al. (2013b)
75	3-*O*-cis-p-coumaric acid	Fructus	Liu et al. (2013a)
Others			
76	Kaempferic acid	Radix	Dai et al. (2016)
Flavonoids			
Flavonols			
77	Icariin	Fructus	Yoshida et al. (1989)
78	Kaempferoside	Fructus	Yoshida et al. (1989)
79	6-methoxy-kaempferol-3-*O*-galactoside	Fructus	Yoshida et al. (1989)
80	Hypericin	Fructus	Yoshida et al. (1989)
81	Rutin	Fructus	Yoshida et al. (1989)
82	Quercetin	Fructus	Yoshida et al. (1989)
83	4′,5,7-trihydroxyflavonol-3-*O*- *β*- d-glucopyranoside	Fructus	
84	Lutein A	Fructus	Yoshida et al. (1989)
Flavan-3-ols			
85	(+)-catechin	Radix	Li et al. (2012)
86	(+)-gallocatechin	Radix	Li et al. (2012)
87	(+)-catechin-8-acetic acid*	Radix	Zeng et al. (2011)
88	Hydroxybenzoic acid- 4-*O*-*β*-d-glucopyranoside	Radix	Li et al. (2012)
89	Dehydrodicatechin A	Radix	Yan et al. (2014)
90	Catechin	Fructus, Radix	Yoshida et al., 1989, Ding et al., 2007
91	Epicatechin	Radix	Ding et al. (2017)
Flavan-3,4-diols			
92	(2R, 3S, 4S)-CIS-Leucine butyl-phthalide	Radix	Li et al. (2012)
93	Guibourtacacidine	Radix	Zeng et al. (2011)
94	Guibourtacacidine 4-methyl ether*	Radix	Zeng et al. (2011)
95	(2R, 3S, 4S)-CIS-Leucine butyl-phthalide	Radix	Li et al. (2012)
96	(2R, 3S, 4S)-CIS-Leucine butyl-phthalide	Radix	Li et al. (2012)
Flavanones			
97	Hesperidin	Fructus	Yoshida et al. (1989)
98	Glycyrrhizin	Fructus	Yoshida et al. (1989)
99	Dihydroapigenin	Fructus	Yan et al. (2014)
Flavones			
100	Apigenin	Fructus	Li et al. (2017)
Others			
101	Phlorizin	Radix	Li et al. (2012)
Tannins			
102	Tannin E*	Fructus	Li et al. (2012)
103	Tannin F*	Fructus	Li et al. (2012)
104	Tannin G*	Fructus	Li et al. (2012)
105	Laevigatin A*	Fructus	Li et al. (2012)
106	Laevigatin B*	Fructus	Li et al. (2012)
107	Laevigatin C*	Fructus	Li et al. (2012)
108	Laevigatin D*	Fructus	Li et al. (2012)
109	Sanguiin H-4	Fructus	Li et al. (2012)
110	Pedunculagin	Fructus	Li et al. (2012)
111	Casuarictin	Fructus	Li et al. (2012)
112	Potentillin	Fructus	Li et al. (2012)
113	Agrimonic acids A	Fructus	Li et al. (2012)
114	Agrimonic acids B	Fructus	Li et al. (2012)
115	Agrimoniin	Fructus	Li et al. (2012)
116	Tellimagrandin Ⅰ	Fructus	Li et al. (2012)
117	Guibourtinidol- (4*α*, 8) - catechin	Radix	Li et al. (2021)
118	Entisetinidol -(4*α*, 6)-catechin	Radix	Li et al. (2021)
119	Fisetinidol-(4*α*, 8) - catechin	Radix	Li et al. (2021)
120	Fisetinidol-(4*β*, 8) - catechin	Radix	Li et al. (2021)
121	Procyanidins B3	Radix	Li et al. (2021)
Other compounds			
122	Rosalaevins A	Fructus	Li et al. (2012)
123	Buddlenol B	Fructus	Li et al. (2012)
124	(-)-simulanol	Fructus	Li et al. (2012)
125	(+)-8-hydroxypinoresinol	Fructus	Li et al. (2012)
126	Euscaphic acid	Fructus	Li et al. (2012)
127	Polystachyol	Fructus	Li et al. (2012)
128	Buddlenol C	Fructus	Li et al. (2012)
129	Diasyringaresinol	Fructus	Li et al. (2012)
130	Threo-guaiacylglycerol-*β*-*O*-40-coniferyl ether	Fructus	Li et al. (2012)
131	Rosalaevins B	Fructus	Li et al. (2012)
132	Erythro-guaiacylglycerol-*β*-*O*-40-coniferyl ether	Fructus	Li et al. (2012)
133	Erythro-guaiacylglycerol-*β*-*O*-40-sinapyl ether	Fructus	Li et al. (2012)
134	4-hydroxy-3-methoxy-benzoic acid	Fructus	Li et al. (2012)
135	Syringaldehyde	Fructus	Li et al. (2012)
136	Vanillin	Fructus	Li et al. (2012)
137	4-Hydroxy-benzaldehyde	Fructus	Li et al. (2012)
138	Protocatechuic acid	Fructus	Wu et al. (2009)
139	Gallic acid	Fructus, Radix	Wu et al., 2009, Yoshida et al., 1989
140	4-acetonyl-3,5-dimethoxy-p-quinol	Fructus	Li et al. (2012)
141	1.2-(1*S*,2*R*)-bis(4-hydroxy-3-methoxy-phenyl)-1,3-propanediol	Fructus	Li et al. (2012)
142	Ellagic acid	Fructus	Li et al. (2012)
143	*β*-Sitosterol	Fructus, Radix	Wu et al., 2009, Ding et al., 2017
144	Carotene	Fructus, Radix	Wu et al., 2009, Ding et al., 2017
145	Glucose	Fructus	Li and Wei, (1997)
146	(*Z*)-3-methoxy-5-hydroxy- stilbene	Radix	Yoshida et al. (1989)
147	(*Z*)-piceid	Radix	Yoshida et al. (1989)
148	PPRLMF-2	Fruit	Zhan et al. (2020)

Note: Compounds with * are newly discovered compounds.

Among these, the triterpenes are its main active ingredients and are characteristic components of this herb.

### 6.1 Triterpenoids

The triterpenoids and their derivatives isolated from this species are divided into three types: the ursane, the oleanolane and lupine-types, of which the uresane-type accounts for the majority. To date, 76 triterpenoids have been identified from Fructus *R. laevigata* and Radix *R.laevigata*, with 57 (1-57) of the ursane-type, 12 (58-69) of the oleanane-type and 6 (70-75) of the lupine-type. In addition, there is one other conformation of triterpene (76), of which there are a total of 10 novel compounds. There are: 3*β*-[(R-l-arabinopyranosyl)oxy]-20*β*-hydroxyursan-28-oic acidδ-lactone (4), 2*ɑ*,3*β*,23-trihydroxy-12,17-dien-28-norursane (6), 2*ɑ*,3*β*,23-trihydroxy-19-oxo-18,19-seco-12,17-dien-28-norursane (7), 2*ɑ*,3*ɑ*,23-trihydroxy-19-oxo-18,19-seco-urs-11.13 (18)-dien-28-oic acid (8), 18,19-split ring-2*α*,3*β*,23*α*-trihydroxy-19-carbonyl-ursane-11.13 (18)-diene-28-carboxylic acid (9), 2*α*,3*α*,24-trihydroxy-urs-12,18-dien-28-oic acid-*β*-d-glucopyranosyl ester (14), 2*α*,3*α*,23-trihydroxy-urs-12.19 (29)-dien-28-oic acid-*β*-d-glucopyranosyl ester (16), 19*α*-OH-3*β*-E-feruloyl corosolic acid (42), 2*α*,3*α*,19*α*,23-tetrahydroxyolean-12-en-28-oic acid 59) and 2*α*,3*β*-dihydrox-yolean-13 (18)-en-28-oic acid (69).

### 6.2 Flavonoids

The flavonoids are widely found in natural plants and can be isolated from Fructus *R. laevigata* and Radix *R. laevigata*, and about 25 compounds have so far been isolated. These flavonoids are subdivided into flavonols (77-84), flavan-3-ols (85-91), flavan-3,4-diols (92-96), flavanones (97-99), flavonoids (100) and one other conformation of flavonoids (101). Of these 25 compounds, two are new and these are (+)-catechin-8-acetic acid (87) and guibourtacacidine 4-methyl ether (94).

The extraction and isolation of the total flavonoids from Fructus *R. laevigata* and Radix *R. laevigata* have been a particular focus of attention and most of these components have already been identified.

### 6.3 Tannins

To date a variety of tannins have been isolated from Fructus *R. laevigata* and Radix *R. laevigata*, including dimeric ellagitannins (102-104), hydrolysable tannins (105-108), condensed tannins (117-121) and a few other tannins (109-116). Among these seven newly discovered tannins have been identified and these are: tannin E-G (102-104), laevigatin A-D (105-108).

### 6.4 Other components

In addition to the three groups mentioned above, Fructus *R. laevigata* and Radix *R. laevigata* also contain lignans (122,131), phenols (123-130,132-137,140-142), catechins (138), benzoic acid derivatives (139), sterols (143-144), stilbene compounds (146-147), polysaccharide 148) and other compounds glucose 145).

## 7 Pharmacology

Pharmacological studies have shown that Fructus *Rosa laevigata* and Radix *Rosa laevigata* have a variety of pharmacological activities, including antioxidant activity and renal protection. Moreover, they play a role in immune regulation and have hypolipidemic, anti-inflammatory, cardiovascular protective and antibacterial activities. In addition, *Rosa laevigata* Michx*.* also has an important role in combating diabetes and it has antiviral and anti-tumour activities. The pharmacological effects of *R. laevigata* and its monomers are summarized in Table 2.

**TABLE 2:** Modern Pharmacological studies of *R. laevigata*.

Effect	Model	Part of plant/Extracts or compound	Positive control	Formulation on/dosage	Result	References
Antioxidant	SOD,MDA	Fruit/TFs (the ethanol extracts)	Fenofibrate (50 mg/kg/day) showed similar *in vivo* antioxidant activity to the extract	*in vivo*: 25.50 mg/kg/day	Decreasing the MDA level, improving the levels of CAT, SOD	Liu et al. (2010c)
	Hydrogen Peroxide-Induced Damage in PC12 Cells	Fruit/TFs	—	*in vitro*: 100–300 μg/ml	Decreasing cytochrome C release from mitochondria into the cytosol and intracellular Ca2+ levels, diminishing intracellular generation of ROS, inhibiting the phosphorylation levels of JNK, ERK and p38 MAPK, down-regulating the expressions of IL-1, IL-6, TNF-α, Fas,FasL, CYP2E1, Bak, caspase-3, caspase-9, p53, COX-2, NF-κB, AP-1, up-regulating the expressions of Bcl-2 and Bcl-xl	Liu et al. (2014); Liu et al. (2019)
	HUVECs	Fruit/TFs	—	*in vitro*: 26, 52, 104 μg/ml	Decreasing caspase-3, -9 activities, decreasing fragmented DNA, increasing the protein expressions of Procaspase-3, Bcl-2, decreasing the expressions of Bak, Bax, Bid and p53	Jia et al. (2012)
Renoprotective effect	streptozotocin-induced diabetic rats	Fruit/aqueous	—	*in vivo*: 5 g/kg/day	Decreasing the levels of malondialdehyde and ROS, inhibiting the expression of nuclear factor-κB p65 and monocyte chemoattractant protein-1 at both the protein and mRNA levels with a concomitant increase in the expression of the IκBα protein	Zhou et al. (2012)
	hypoxia/reoxygenation (H/R) model in NRK-52E cells and ischemia-reperfusion model in rats	Fruit/TFs	—	*in vivo*: 50, 100 and 200 mg/kg	Decreasing the levels of MDA, ROS, GSH, GSH-Px, up-regulating the level of Sirt 1, Nrf2 and HO-1, down-regulating Keap1, NF-κBp65, decreasing the level of the mRNA levels of IL-1β, IL-6 and TNF-α	Zhao et al. (2016)
Immunomodulation	RAW 264.7 cells in mice	Dried pulp/Polysaccharides	LPS-treated cells (20 μg/ml) showed similar *in vitro* activity to the extract	*in vitro*: 50–400 μg/ml	Up-regulating the p-ERK, p-JNK, p-p38, and p-p65	Zhan et al. (2020)
	AChE inhibitory activity	Fruit/ethanol	IC_50_ value of galanthamine was 36.30 μg/ml	*in vitro:* 2–200 μg/ml	Compounds 1 and 2 showed potent AChE inhibitory activities with IC50 values of 29.22 and 45.47 μg/ml, respectively. At high concentration, compounds 1 and 2 produced a 92% and 89% inhibition on the target enzyme	Gao et al. (2018)
Lipid-decreasing	high-fat diet-induced non-alcoholic fatty liver disease in rats	Fruit/TFs	silymarin (200 mg/kg, dissolved in water) and HFD	*in vivo*: 80, 160 mg/kg	Decreasing the relative liver weight, serum AST and ALT activities, the levels of serum lipid, LDL, blood glucose and insulin, suppressed lipid accumulation in liver, increasing serum HDL level, enhancing SOD activity, increasing GSH-Px and GSH contents, decreasng the concentration of MDA and CYP2E1 expression as well as preventing mitochondrial membrane potential dysfunctions and ultrastructural alterations	Zhang et al. (2013a)
	FADS2, ACOX3, SCD-1	Fruit/ethanol	—	*in vivo: HFD* + 800 mg/kg RLP and HFD +200 mg/kg RLP	Decreasing the serum lipid levels, elevate the serum high-density lipoprotein cholesterol levels, enhancing the antioxidant enzymes levels, upregulating of FADS2, ACOX3 and SCD-1	Zhang et al. (2020)
	Mouse obesity model	Fruit/ethanol	—	*in vivo*: 0.00625、0.012、 0.025、0.05、0.1、0.2、0.4、0.8、1.6 mg/ml	Body mass (*p* < 0.05), GLU and LDL-C levels were significantly lower (*p* < 0.01) and HDL-C was significantly higher (*p* < 0.01) in the drug-administered group	Li J. et al. (2019)
Anti-inflammatory	RAW 264.7 cells in mice	Leaves/ethanol	Hydrocortisone (50 lg/mL) showed similar *in vitro* activity to the extract	*in vitro*: g 0.5, 5, 10, 20, 50 and 100 μg/ml	The compound 12 exhibited moderate inhibition on NF-jB transcriptional activity with an IC50 value of 23.21 lM. The IC50 values of compound 12 were measured as 14.32, 8.53, 8.04, and 10.38 lM for the inhibitory activity on TNFarelease, IL-1b-release, IL-6-release, and IL-10-release, respectively	Yan et al. (2013)
	PM10-induced lung inflammatory disease model	Fruit/aqueous	—	*in vitro*: 125 and 250 μg/ml	Suppressing the expression level of MAPK/NF-κB pathways and its downstream signal, COX-2 in PM10-induced A549 cells, down-regulating the mRNA expression level of inflflammatory cytokines (TNF-α, IL-1β, IL-6, IL-13, and IL-17) in PM10-induced A549 cells	Ko et al. (2020)
	*in vitro* and *in vivo* model of allergic asthma	Fruit/aqueous	Dexamethasone	*in vivo*: 50, 100 mg/kg	Suppressing NF-κB activity and COX-2 expression levels in EGF-induced A549 cells	Lee et al. (2020)
	Xylene-induced auricular swelling test in mice, Agar granulation tissue proliferation test in mice	Radix/aqueous	—	*in vivo*: 0.2 ml/10 g	a certain inhibitory effect on xylene-induced auricular swelling in mice, with an inhibition rate of 9.86%, a strong inhibitory effect on agar-induced granulation tissue proliferation in mice. The inhibition rate of swelling reached 22.39%	Zou and Xie (2011)
Cardiovascular protective effect	cardiotoxicity induced by adriamycin in rats	Fruit/aqueous	—	*in vivo*: 1, 3, 5 g/kg	Upregulating the expression of the CuZn-SOD mRNA level, enhancing the activity of GSH-PX, CAT and T-SOD, inhibiting adriamycin-induced apoptosis, bax mRNA expression, increasing bcl-2 expression and bcl-2/bax ratio	Luo. (2014)
	myocardial infarction rat model	Fruit/ethanol	—	*in vivo*: 300 mg/kg weight/day in 2 ml of water	Restore the decreased cardiac function	Qu et al. (2015)
	Acute blood stasis model in rats	Fruit/TFs ethanol	—	*in vitro*: 0.06, 0.12, 0.24 g/kg	Reduce the viscosity of whole blood in rats. It can significantly reduce whole blood viscosity and inhibit platelet aggregation in rats	Chen et al. (2014)
Antibiotic	S.aureus, E. coli, *B. subtilis*	Fruit/polysaccharides, flavonoids	—	*in vitro*: 1.57, 3.13, 6.25, 12.5, 25, 50, 100 mg/ml	MICs of polysaccharides of R. laevigata on these three bacteria were 25, 50 and 3.13 mg/ml, MICs of flavonoids for these three bacteria were 3.13, 12.5 and 6.25 mg/ml	Liu et al. (2009)
	punching method in agar diffusion method	Radix, stem/polysaccharides	—	*in vitro*: 10, 20, 30, 40 mg/ml	Polysaccha ride s of the ro ot and stem of R. laevigata had inhibitory action on *staphylococcus* albus, staphy lococcus citreus, *Staphylococcus aureus*, standard *Klebsiella pneumoniae* and dysentery *bacillus* in a dose-dependent manner	Lai et al. (2009)
	doubling dilution method and inhibition zone	Radix/aqueous, 75% ethanolic extract, petroleum ether extract, chloroform extract, ethyl acetate extract	—	*in vitro*: 400 mg/ml	The MIC values of water extract of RRL Againsts *Staphylococcus aureus* (*S. aureus*), *Pseudomonas aeruginosa* (*P. aeruginosa*), *Proteus* vulgaris (P. vulgaris), *Salmonella* (SA) typhi were 10, 40, 10,40 mg/ml, the MIC inhibitory concentration values of 75%alcohol extract of RRL against *S. aureus*, *Shigella* Castellani and Chahners (S.C), P. vulgaris, SA typhi were 10, 10, 10, 20 mg/ml, the MIC value of ethyl acetate extract of RRL against only *S. aureus* is 20 mg/m1, Petroleum ether extract and chloroform extract of RRL have not antibacterial effect	Lai et al. (2012)
Anti-tumor	HL-7702 and BEL-7402 cell lines	Fruit/flavonoids (rutin and quercetin)	—	*in vitro*:/	The half-inhibitory concentration of Quercetin and Rutin on human liver cancer BEL-7402 cells *in vitro* was (7.625 ± 2.02) μmol/L, (29.91 ± 3.05) μmol/L, the half-inhibitory concentration of Querceti n and Rutin on HL-7702 was (35.54 ± 4.37) μmol/L, (29.91 ± 3.05) μmol/L	Huang and Liu. (2017)
	HL-7702 and BEL-7402 cell lines	Fruit/polysaccharides	—	*in vitro*:/	the half-inhibitory concentrations of RLP to HL-7702 and BEL-7402 was (31.04 ± 3.68) μmol/L, (12.43 ± 1.95) μmol/L	Huang and Liu. (2015)
	Mice tumor model	Radix/polysaccharides	5-Fu (20 mg kg^−1^) showed similar *in vivo* activity to the extract	*in vivo:* 50, 100, 200 mg kg^−1^	The life-prolonging rates of JYG polysaccharides (low, medium, high) were 3.07% ± 2.35%, 10.1L%±4.32%, 34.3% ± 8.31%	Feng and Tian. (2011)
Antiviral	RSV, HSV-1, COX-B5, EV71	Fruit/ethanol, petroleum ether extract, dichloromethane extract, Ethyl acetate extract, n-butanol extract, aqueous	Ribavirin (50 mg mL^−1^) showed similar *in vitro* activity to the extract	*in vitro*: 16 mg mL^−1^	Ethyl acetate extract showed good direct killing effect on RSV with TI value of 19.333, which was close to that of the ribavirin positive control (19.760). N-butanol extract showed direct killing effect on COX-B5 with TI value of 16.622, which was close to that of the ribavirin positive control (17.562). Alcohol extract showed best effect on HSV-1 with TI value of 18.922, which was close to that of the acyclovir positive control (23.742)	Liu et al. (2017a)
	RSV, EV71	Fruit/aqueous, acetone extract, methanol extract, 60% ethanolic extract	Ribavirin (50 mg mL^−1^) showed similar *in vitro* activity to the extract	*in vitro*: 16 mg mL^−1^	The three ultrasound extracts inhibited both viruses, and the effect on EV71 was better than that on RSV. The inhibition of both viruses was better than the other two extraction methods, with TI values of 6.10 and 6.19 respectively. The inhibition of both viruses by 60% ethanol ultrasound extract was better than the other two extraction methods, with TI values of 6.10 and 6.19, respectively; the inhibition of both viruses by acetone ultrasound extract was worse, with TI values of 4.51 and 5.08, respectively	Li N. et al. (2019)
	RSV, COX-B5, EV71	Fruit/polysaccharides	Ribavirin (50 mg mL^−1^) showed similar *in vitro* activity to the extract	*in vitro*: 16 mg mL^−1^	The TI values of RSV, COX-B5 were 80.895 and 41.541 respectively	Liu et al. (2017b)
Diabetes	SRA01/04 cell models of diabetic cataract	Fruit/aqueous	—	*in vitro*: 0.1, 5 and 10 g/L	Under high glucose conditions, R. laevigata inhibited ROS production and increased MMP by inducing HO-1 expression. This effect was mediated by the PI3K/AKt and Nrf2/ARE pathways, which are lost if one of these pathways when they are inhibited	Liu et al. (2015)
	LEC apoptosis of diabetic cataract rats	Fruit/aqueous	—	*in vivo*: 400 mg/kg	Inhibit the apoptosis of rat diabetic cataract lens epithelial cells by increasing the Bcl-2/Bax expression ratio	Zhou et al. (2014)
	*In vitro* DNS glucose uptake and Western blotting assays	Fruit/ethanol, in water, n-butanol, ethyl acetate and n-hexane)	Insulin or metformin	*in vitro*:/	The ethanolic extract of R. laevigata and its derivative sub-fractions significantly increased glucose uptake in hepatocytes, and the bioactive glutamatergic ingredients significantly increased glucose uptake in insulin resistant cells and promoted insulin sensitivity	Kumar et al. (2021)

### 7.1 Antioxidant activity

The antioxidant activity exhibited by *R. laevigata* depends on its ability to scavenge free radicals and cause the reduction of metal ions. Studies have shown that Fructus *R. laevigata* is rich in total flavonoids (TFs), a component that is closely associated with antioxidant activity. This suggests that TFs in *R. laevigata* may be a rich source of natural antioxidants for the prevention and treatment of diseases caused by oxidative stress such as cardiovascular disease (Li et al., 2021). Liu et al. (2010b) found that TFs of Fructus *R. laevigata* have a scavenging effect on 2,2-diphenyl-1-picrylhydrazyl (DPPH), hydroxyl and superoxide anion radicals and they have a strong reducing ability. Intragastric administration of TFs (25, 50 mg/kg/day) to hyperlipidemic mice for 4 weeks, showed that TFs significantly increased the levels of several antioxidant substances including catalase (CAT), superoxide dismutase (SOD), GSH and GPX in the liver. It was also seen that TFs significantly reduce hepatic malondialdehyde (MDA) concentrations in a dose-dependent manner.

Liu et al. (2014, 2019) also found a protective effect of TFs of Fructus *R. laevigata* against hydrogen peroxide-induced cell damage. The data showed that TFs down-regulated the expression of CYP2E1, iNOS, nuclear factor-kappab (NF-*κ*B), BaK and caspase-3 and significantly reduced the mRNA levels of TNF-α and Fas/Fasl. In addition, Jia et al. (2012) concluded that TFs of Fructus *R. laevigata* can reduce the expression of fragmented DNA, Bax, Bid and p53 as well as activity the activities of caspases-3 and -9. They also increased the protein expression of procaspase-3 and Bcl-2. It is clear that these three mechanisms of antioxidant activity are similar. In summary, TFs can exert antioxidant activity by reducing oxidative stress, cell inflammation and apoptosis, and there is a good quantitative relationship between the TF content and antioxidant activity at a range of concentrations.

There are also several flavonoid quercetin and proanthocyanidins that have antioxidant activity and their scavenging effects of free radicals are also strong. In addition, quercetin has some inhibitory effects on linoleic acid peroxidation, and proanthocyanidins have inhibitory effect on lipid oxidation (Xiao et al., 2010; Chen et al., 2013). Of course, the flavonoids are not the only compounds to have antioxidant activities in *R. laevigata*, and the others are the total phenols. Meng et al. (2012) found that total phenols have strong scavenging ability for 2.2′-azino-bis (3-ethylbenzothiazoline-6-sulfonic acid) (ABTS) radicals. They also have strong copper ion reduction and metal chelating abilities, Moreno et al. (2007), Apak et al. (2004) and, Du et al. (2009) investigated the ABTS scavenging, copper ion reducing, and metal chelating abilities, respectively. Their results showed a correlation between total phenolic content and antioxidant activity.

From a review of the relevant literature, it san be seen that polysaccharides in Fructus *R. laevigata* also have antioxidant activity. When the protection rate of vegetable and animal oils was approximately 50%, the mass concentration of polysaccharides was 2.8 and 5.9ng/ml, respectively. The scavenging of hydroxyl radicals by polysaccharides was 4.7 ng/ml when the mass concentration of *R. laevigata* was at 50%. The scavenging rate of superoxide anion radicals by the same concentration of polysaccharides of Fructus *R. laevigata* was only 23%. In addition the reduction ability of polysaccharides of *R. laevigata* to Fe^3+^ was also found to be very strong. The results showed that the antioxidant activity of Fructus *R. laevigata* had a good quantitative relationship with the polysaccharide content. However, a single approach cannot fully demonstrate the extent of the antioxidant capacity of a substance. Therefore, it is not always straightforward to correlate the antioxidant capacity of different substances (Wang Y. M. et al., 2016).

### 7.2 Renoprotective effects

Fructus *R. laevigata* is believed to be able to improve kidney health. There are many studies that have verified the kidney-protective effects of this herb. Zhou et al. (2012) investigated the effects of *R. laevigata* on renal oxidative stress in streptozotocin-induced diabetic rats. The results showed that *R. laevigata* significantly improved the renal dysfunction of these diabetic rats. The mechanism involves an increase in SOD and total antioxidant activities and a reduction in the levels of MDA and reactive oxygen species (ROS). The expression of nuclear factor-*κ*B p65 and monocyte chemotactic protein-1 was inhibited at the protein and mRNA levels, respectively, while the expression of I*κ*B*α* protein was increased. Zhao et al. (2016) found that TFs in *R. laevigata* have a significant nephron-protective effect on ischemia-reperfusion injury (IRI) by affecting the Sirt1/Nrf2/NF-*κ*B signaling pathway. After treatment with *R. laevigata*, SOD activity and total antioxidant power increased and the levels of MDA and ROS decreased in rats with kidney injury, achieving an overall reduction in kidney injury. Thus, the effect of adminishtration of *R. laevigata* extracts on kidney damage is mainly dependent on Sirt 1.

### 7.3 Immunomodulation

In recent years, a number of studies have shown that the ethanol extracts of Fructus *R. laevigata* can have some immunological activity. Zhan et al. (2020) identified a novel acidic polysaccharide, PPRLMF-2, which recognizes pattern recognition receptors (PRRs) of macrophages and enhances their immunomodulatory activity by activating MAPKs and NF-*κ*B signaling pathways. The mechanism of action is that PPRLMF-2 can significantly increase the phagocytosis and the secretion of cytokines in murine RAW264.7 cells lines. In addition, SR, GR, Toll-like receptor-2 (TLR-2) and Toll-like receptor-4 (TLR-4) are the major PRRs that upregulate the expression of p-Extracellular signal-regulated kinase (p-ERK), p-c-Jun N-terminal kinase (p-JNK), phospho-NF-*κ*B p38 (p-p38) and phospho-NF-*κ*B p65 (p-p65). In addition, Gao et al. (2018) also found the presence of polyhydroxy triterpenoids which have anti-acetylcholinesterase activity in extracts from *R. laevigata*. Experiments showed that two pure polyhydroxytriterpenes, 1 and 2, were isolated by the authors and these showed potent anti-acetylcholinesterase activities. Diao et al. (2014) studied the effects of fermented *R. laevigata* on the immune performance of weaned piglets. They showed that fermented *R. laevigata* could significantly increase the levels of immunoglobulin IgA, IgM and IgG in these animals (the most suitable concentration was 0.2%) and this improved the immunity of the weaned piglets.

Peng et al. (2014) performed a study on the immunomodulatory activity of Radix and Rhizome *R. laevigata* of different origins. Their results showed that the hydroextracts of Radix *R. laevigata* had some specific immunosuppressive activities. It was concluded that different medicinal parts of *R. laevigata* different origins had similar immunological activities without any significant differences.

### 7.4 Lipid-decreasing

TFs components of Fructus *R. laevigata* have received more attention as lipid-lowering. Zhang et al. (2013a) investigated the effects of TFs in *R. laevigata* on non-alcoholic fatty liver disease (NAFLD) induced by high-fat diets in animal studies. The results suggest that TFs inhibit hepatic fat accumulation by suppressing the expression of some key molecules in the fatty acid synthesis pathway and promoting the *β*-oxidation fatty acids. They also showed that TFs did not achieve lipid-lowering activity by inhibiting cholesterol synthesis. In addition, Liu Y. et al. (2010) also found that TFs had strong hypolipidemic activity and the levels were governed partly by enhancing the antioxidant system.

A review of other related literature revealed that the polysaccharides in *R. laevigata* also have hypo-lipidemic activity. Zhang et al. (2020) found that low molecular polysaccharides extracted from the fruits of *Rosa laevigata* (RLPs) reduced serum lipid levels and increased those of HDL cholesterol. After further study, the results showed that RLPs (with molecular weights of 9,004, 8,761 and 7,571 Da) exhibited significant hypolipidemic effects in high-fat diet-induced muscle, and they did so by integrating with the PPPAR signaling pathway to improve lipid metabolism dysfunction. The study also illustrated that high doses of RLPs havd stronger hypo-lipidemic effects, and these were more positive when compared to that of polymeric polysaccharides. Another paper also investigated the hypo-lipidemic activity of the polysaccharide, RLP-2 (21.5 kDa) (Yu et al., 2013). They found that the mechanism of action in both studies were similar, and further studies determined that the strength of the activities of these polysaccharides verified the effects of molecular weight on the hypolipidemic activity. In addition, the total polyphenols and saponins in *R. laevigata* also have been shown to have some hypo-lipidemic activity (Dong et al., 2019; Li J. et al., 2019). However, because the mechanism of lipid synthesis is complex and it has been difficult to identify a specific major factor or mechanism to explain the hypo-lipidemic activity of *R. laevigata*, so further studies still need to be performed.

### 7.5 Anti-inflammatory

Among the studies on the anti-inflammatory activity of Fructus *R. laevigata*, triterpenoids have received the most attention. Yan et al. (2013) investigated the anti-inflammatory activity of triterpenoids by using the LPS macrophage-induced luciferase assay. Binding of NF-*κ*B to the *κ*B site can control cytokine expression, and the mechanism by which triterpenoids inhibit NK-*κ*B activation may be determined by performing NF-*κ*B luciferase assays in NF-*κ*B-luc293 cells. The triterpenoids 4, 9, 11, and 12 in this study were found to have inhibitory effects on cytokine release in LPS-stimulated mouse macrophages RAW264.7. It was also found that compound 12 had a much stronger anti-inflammatory activity than compound 11. This was probably, due to the presence of a hydroxyl group at C-23, which may have greatly reduced the release of inflammatory factors. Compound 12 showed moderate inhibition of the transcriptional activity of NF-*κ*B with an IC_50_ of 23.21 μM. Its inhibitory activities against TNF*α*-release, IL-1*β*-release, IL-6-release and IL-10-release were 14.32, 8.53, 8.04 and 10.38μM, respectively. The presence of glucoside in this compound may have caused an increase in the anti-inflammatory activity.

Several scholars have also found anti-inflammatory effects of *R. laevigata* in animal disease models. Ko et al. (2020) found that *R. laevigata* inhibited the expression levels of MAPK/NF-*κ*B pathways and its downstream signal COX-2 in PM10-induced A549 cells in an induced lung inflammatory disease model. In this dhtudy, *R. laevigata* was shown to reduce the pro-inflammatory factors activated by PM10. The results in the literature suggest that *R. laevigata* alleviates PM10-induced lung inflammation, and the mechanism may be that pretreatment with this herb medicine inhibits PM10-induced activation of MAPK phosphorylation and suppresses nuclear translocation of NF-*κ*B p65. In contrast, in a mouse model of chronic inflammation, Lee et al. (2020) investigated whether *R. laevigata* exhibited anti-inflammatory effects associated with acute asthma in both *in vitro* and *in vivo* experiments. Their results showed that after RLM pretreatment, which significantly inhibited EGF-induced NF-*κ*B activity and COX-2 expression levels in A549 cells, inflammatory cells, IgE secretion and related substances were reduced in the model in a dose-dependent manner to reduce allergic airway inflammation. The anti-inflammatory mechanism seen may act through the inhibition of IgE and related cytokines.

It was not only Fructus *R. laevigata* which had anti-inflammatory activity, but also Radix *R. laevigata*. Its chemical composition is high in tannins, which play an important role as anti-inflammatory agents (Zou and Qiu, 2011). Zou and Qiu (2011) established a model and found Radix had a better anti-inflammatory effect by measuring its effect on NO release from lipopolysaccharide-induced peritoneal macrophages in mice, and this effect was related to the inhibition of NO release.

### 7.6 Cardiovascular protective effect

The protective effects of Fructus *R. laevigata* on cardiovascular and cerebrovascular are mainly due to the antioxidant and anti-inflammatory activities of its active ingredients. For example, Luo et al. (2014) found that *R. laevigata* could increase the mRNA expression levels of CuZn-SOD protein as well as glutathion peroxidase (GSH-PX), catalase (CAT) and superoxide dismutase (SOD) activities in myocardial tissue. High doses of *R. laevigata* reduced myocardial apoptosis induced by adriamycin and enhanced Bcl-2 gene expression and decreased Bax levels. This confirmed the protective effects of *R. laevigata* on adriamycin-induced myocardial injury. Liu et al. (2010a) found that high doses of oral TFs reduced total cholesterol (TC), triglycerides (TG), low-density lipoprotein cholesterol (LDL-C) and high-density lipoprotein cholesterol (HDL-C).

Jia et al. (2012) demonstrated the protective effects of TFs in *R. laevigata* against hydrogen peroxide-induced injury in human umbilical vein endothelial cells. Qu et al. (2015) established a myocardial infarction (MI) rat model to study the ability of the active ingredients of *R. laevigata* in the treatment of this condition. The results showed oral administration of the active ingredients to MI-induced animals gradually restored the decline in cardiac function caused by MI and induced the myocardial regeneration and replacement of necrotic heart tissues. The authors hypothesized that the mechanism of myocardial regeneration induced by the active ingredients of *R. laevigata* may be the multiple properties of the active ingredients in anti-inflammatory and anti-oxidative stress. This promoted cell survival and prevented IRI. However, the action of the specific ingredients and the specific mechanisms involved are still unclear and need to be further investigated. Chen et al. (2014) found that medium and high concentrations of TFs significantly reduced whole blood viscosity in rats and had some inhibitory effects on platelet aggregation, thus exerting antithrombotic effects. Additionally, Luo et al. (2014) found that *R. laevigata* could improve doxorubicin (DOX)-induced myocardial toxicity and that co-administration with cloxacin (LOS) provided better myocardial protection. This mechanism may also be related to the anti-inflammatory and antioxidant effects of this plant.

### 7.7 Antibiotic effect

One of the many pharmacological effects of Fructus *R. laevigata*, such as its antibacterial effect, also plays an important role in daily life. *Staphylococcus aureus*, *Bacillus subtilis* and *Escherichia coli* are considered to be serious modern day hazards and these can affect food hygiene and safety. Liu et al. (2009) preliminarily studied the inhibitory effect of extracts of polysaccharides and flavonoids from the fruits of *R. laevigata* on these three species of bacteria. The results showed that both extracts had some antibacterial effects, with the polysaccharide having the best antibacterial effect on *E. coli* (MICs of polysaccharides of *R. laevigata* on these three bacteria were 25, 50 and 3.13 mg/ml, respectively). This was followed by *S. aureus*, while the inhibitory effect of flavonoids was exactly the opposite (MICs of flavonoids for these three bacteria were 3.13, 12.5 and 6.25 mg/ml, respectively). Both the polysaccharides and flavonoids showed the weakest inhibition with *B. subtilis*. However, the relationship between its inhibitory mechanism and its specific structural composition needs to be further investigated. A review of the relevant literature revealed that the roots and stems of *R. laevigata* are also known to possess antibacterial activities. The acetone extract of *R. laevigata* had an inhibitory effect on *Streptococcus mutans* which usually exist in the oral cavity, and the inhibitory effect of this extract was mainly derived from the tannin component of *R. laevigata* (Li, 2006).

Lai et al. (2009) determined the bacterial inhibitory effects of the roots and stems polysaccharides of *R. laevigata* using the punch-hole method and an agar diffusion technique. The results showed that the polysaccharides in the roots and stems of *R. laevigata* inhibited *Staphylococcus albicans, Staphylococcus citricola, S. aureus, Klebsiella pneumoniae and Bacillus dysenteriae* in a dose-dependent manner. There were also no significant differences in the inhibitory effects of extracts from the two sites. In addition, Lai et al. (2012) also investigated the differences in the antibacterial effects between extracts obtained from using different polar solvents of the roots. The results showed that the aqueous and alcoholic extracts of Radix *R. laevigata* had better antibacterial effects, mainly due to the higher content of flavonoids, tannins and triterpenoids in these two extracts.

### 7.8 Anti-tumor activity

Huang and Liu. (2017) studied the effects of two flavonoid active components of Fructus *R. laevigata*, rutin and quercetin, on the proliferation of human hepatocellular carcinoma cells cultured *in vitro*. Both flavonoids were found to exert some inhibitory effect on the proliferative effects of human hepatoma cells BEL-7402 cultured *in vitro*, with rutin and quercetin having IC_50_s of 29.91 ± 3.05 and 7.625 ± 2.02 μmol/L, respectively. There were also less toxic to normal human hepatocytes cultured *in vitro* HL-7702, with IC_50_s of 29.91 ± 3.05 and 35.54 ± 4.37 μmol/L, respectively. Others also investigated the *in vitro* antitumor activity of polysaccharides from *R. laevigata* and found that these compounds had some inhibitory effects on human hepatocellular carcinoma cells cultured *in vitro* (Huang and Liu, 2015). The inhibitory effect on the proliferation of human hepatocellular carcinoma cells, BEL-7402, cultured *in vitro*, had an IC_50_ of (12.43 ± 1.95) μmol/L. There was a lower toxicity to normal human hepatocytes, HL- 7,702, *in vitro*, with an IC_50_ of (31.04 ± 3.68) μmol/L. Therefore, it was also concluded that the polysaccharide compounds in *R. laevigata* also had some anti-tumor activity.

However, polysaccharides in Radix *R. laevigata* had no significant therapeutic effect when used directly as an anti-tumour drug. However, when combined with 5-Fu, it had a significant potentiation and toxicity reduction effect (Feng and Tian, 2011), suggesting that polysaccharides in the root of *R. laevigata* may act as a potential anti-tumour adjuvant.

### 7.9 Antiviral activity

Liu et al. (2017a) studied the direct inactivation, prophylaxis and post-viral penetration inhibition of respiratory syncytial virus (RSV), herpes simplex virus-1 (HSV-1), coxsackie B5 (COX-B5) and enterovirus 71 (EV71) by different extracts of Fructus *R. laevigata*. Their results showed that Fructus *R. laevigata* had no preventive effect and no post-penetration inhibition against these viruses. The direct inactivation of RSV by ethyl acetate extract was good with a therapeutic index (TI) of 19.333, which was comparable to that of the positive control, ribavirin (TI of 19.760). The n-butanol extract was able to directly inactivate COX-B5 with a TI of 16.622 and the positive control, ribavirin, with a TI of 17.562. The alcohol extract was most effective against HSV-1 with a TI of 18.922 and the positive control, acyclovir, with a TI of 23.742, while there was no direct inactivation of EV71. However, when using the sonicated of *R. laevigata* in acetone, methanol and ethanol it was found that the 60% sonicated ethanol extract was relatively more effective against RSV and EV71 than the other two (Li N. et al., 2019).

Liu et al. (2017b) also investigated the *in vitro* antiviral activity of polysaccharide in *R. laevigata* and studied their inhibitory effects on RSV, COX-B5 and EV71. They showed that the polysaccharides had *in vitro* anti-RSV, COX-B5 and EV71 activities, and the anti-RSV and COX-B effects were better than those of the positive control drug ribavirin, with TI of 80.895 and 41.541, respectively. Also some studies have shown that a combination of polysaccharides of *R. laevigata* and adriamycin has a significant potentiation and toxicity reduction effect, which could significantly increase the tumour inhibition rate. In addition, some studies have shown that the active ingredients of *R. laevigata* against HSV is a polyhydroxyl pigment (Luo et al., 1989). However, the active ingredients against other viruses have not yet been identified and further research is still needed in this area.

### 7.10 Diabetes

Diabetes is now a “very common” disease and is increasingly being researched and the active use of herbal medicines to treat diabetes and its related diseases are increasing. Liu et al. (2015) investigated the effects of Fructus *R. laevigata* on the production of ROS and the mitochondrial membrane potential (MMP) in lens epithelial cells under high glucose conditions by establishing a cell model of SRA01/04 in diabetic cataract. The results showed that under high glucose conditions, *R. laevigata* inhibited ROS production and increased MMP by inducing HO-1 expression. This effect was mediated by the PI3K/AKt and Nrf2/ARE pathways, which are lost if one of these pathways when they are inhibited. Thus it was concluded that *R. laevigata* can play an important role in the treatment of diabetic cataract.

In a study by Zhou et al. (2014), Fructus *R. laevigata* was found to inhibit the apoptosis of rat diabetic cataract lens epithelial cells by increasing the Bcl-2/Bax expression ratio, and thus delaying the onset and development of diabetic cataract. In addition, Kumar et al. (2021) used *in vitro* DNS glucose uptake and western blotting analysis to study the hypoglycaemic effects of ethanolic extracts of *R. laevigata* and its derivative subfractions (in water, n-butanol, ethyl acetate and n-hexane). They studied the effects of its main bioactive compound, glutarone, on normal and high glucose-induced insulin-resistant hepatic HepG2 cells. The results showed that the ethanolic extract of *R. laevigata* and its derivative sub-fractions significantly increased glucose uptake in hepatocytes, and the bioactive glutamatergic ingredients significantly increased glucose uptake in insulin resistant cells and promoted insulin sensitivity. This led them to conclude that *R. laevigata* had an important role in hypoglycaemia and that it can also be effective in improving the hyperglycaemic and hyperlipidemic states of patients with type Ⅱ diabetes (Deng and Zhang, 2018).

### 7.11 Other activities

The liver is the largest metabolic organ in the body and is associated with a large number of diseases related to its damage and hepatotoxicity. Many studies have revealed that the mechanism of action of Fructus *R. laevigata* in protecting liver tissues is most closely linked to their anti-inflammatory activity, antioxidant capacity and resistance to oxidative stress. Generally, the main components that exert hepatoprotective effects are the TFs. For example, Zhang et al. (2013b) studied the protective effect of TFs on CCl_4_-induced hepatotoxicity in mice and found that pretreatment with TFs reduced the expression of CYP2E1, iNOS, NF-*κ*B, Bak, Caspase-3, TNF-ɑ and Fas/FasL, and upregulated the levels of Bcl-2. This reduced the incidence of liver lesions and improved the abnormalities of hepatocytes, thus achieving a protective effect on the liver. In addition, TFs significantly reduced the CCl_4_-induced increase in aspartate aminotransferase (AST) and alanine transferase (ALT) activities. The TFs of *R. laevigata* also exerted protective effects against lipopolysaccharide (LPS)-induced liver injury in mice by modulating FXR signaling (Dong et al., 2018). They also significantly reduced serum ALT, AST, total triglycerides (TG), total cholesterol (TC) levels and the relative liver weight, resulting in improvement of pathological changes in the liver.

TFs could also significantly reduce tissue MDA levels and increased SOD and GSH-Px levels, and a mechanistic study showed that these compounds significantly increased nuclear erythroid-like factor 2-related factor 2 (Nrf2), heme oxygenase 1 (HO-1), NAD(P)H dehydrogenase (quinone 1) (NQO1), glutamate-cysteine ligase catalytic (GCLC) subunit and glutamate-cysteine ligase regulatory (GCLM) subunit expression levels. They also decreased the expression levels of ECH-like associated protein 1 (Keap1). Additionally, TFs significantly inhibited the nuclear translocation of NF-*κ*B and subsequently reduced the expression levels of interleukin (IL)-1*β*, IL-6, high mobility group box 1(HMGB-1) and cyclooxygenase-2 (COX-2) by activating FXR and forkhead box O3 (FOXO3a) against inflammation. They could notably reduce the expression levels of sterol regulatory element binding protein-1c (SREBP-1c), acetyl coenzyme a carboxylase-1 (ACC1), fatty acid synthase (FASN) and stearoyl coenzyme A desaturase 1 (SCD1). These compounds increased the expression levels of carnitine palmitoyltransferase 1 (CPT1) by activating FXR to regulate lipid metabolism. In addition to TFs, the total saponins (TSs) in Fructus *R. laevigata* also had a protective effect against CCl_4_-induced acute liver injury in mice. A study showed that pretreatment with these compounds significantly reduced the protein expression levels of CYP2E1, ATF6, GRP78, EIF2, COX-2, NF-*κ*B, p53, Caspase-9 and cytokeratin 18 and the phosphorylation levels of MAPKs. They also significantly reduced the gene expression of TNF-ɑ, IL-6, Fas/FasL and Bax. Like TFs, TSs also upregulated Bcl-2 expression and also reduced serum AST and ALT activities, therefore exerting a hepatoprotective effect by these mechanisms (Dong et al., 2013).

Dong et al. (2015) found a protective effect of TSs against CCl_4_-induced hepatic fibrosis. The mechanism may be that TSs act as an anti-fibrotic agent by down-regulating matrix metalloproteinases for matrix degradation and also by modulating the signaling pathways related to TGF*β*/Smad, focal adhesion kinase 1 (FAK), phosphatidylinositol three kinase (PI3K) protein kinase B (Akt)-p70S6 kinase and MAPKs. Liu et al. (2010b) found that TFs of Fructus *R. laevigata* also had a protective effect against paracetamol-induced liver injury. Dong et al. (2014) investigated the effects of TSs in *R. laevigata* on liver injury together with acetaminophen and showed that TSs exerted a protective effect against acetaminophen-induced liver injury by inducing autophagy and anti-inflammatory effects as well as apoptotic cell death.

Xie et al. (2001) found strong scavenging effects of Fructus *R. laevigata* extracts on NO^2-^ under pH acidic conditions that simulated gastric juice production. It has also been found that TFs and the polysaccharide, TNBS, from Fructus *R. laevigata* exerted mucosal protective effects in mice with Crohn’s disease by reducing the weight/length ratios of their colons. The TNF-ɑ and IFN-γ levels in the colonic tissues were also reduced (Bian et al., 2018). An aqueous extract of Fructus *R. laevigata* was also found to play an important role in the urinary system, which may be of benefit in reflex incontinence (Lu et al., 1995). In addition, it was shown that a compounded extract of Fructus *R. laevigata* could effectively treat white diarrhoea in piglets in a dose-dependent manner (Xiang et al., 2015).

One study also found that the alcoholic extract of Radix *R. laevigata* exhibited significant tolerance to hypoxia and prolonged the survival periods of mice after its administration (Huang and Liu, 2015).

## 8 Toxicology

It is well known that many herbs have therapeutic effects as well as adverse reactions. Relatively few toxicity studies have been reported for *Rosa laevigata* Michx., but as it becomes more widely used, such issues should receive more attention and raise serious questions about its safety in clinical settings. Zhang et al. (2012) studied the subchronic toxicity of TFs obtained from *R. laevigata* in a 90-day subchronic toxicity study in rats given oral doses of 500, 1,000 and 2000 mg/kg/day of TFs. Toxicity assessment of TFs based on ophthalmic examination, body weight, feed and water consumption, urinalysis, haematology, clinical biochemistry and pathology were performed in rats. No signs of toxicity of TFs were observed at doses of 500 and 1,000 mg/kg/day, while a decrease in platelet count and an increase in cardiomyocyte voids were found in rats at a dose of 2000 mg/kg/day compared to the control group. In the 2000 mg/kg/day dose group, the relative weight of cardiomyocytes increased significantly in male rats, while the absolute and relative weight of the adrenal glands decreased significantly in female rats at doses of 1,000 and 2000 mg/kg/day. The analysis concluded that at a dose of 1,000 mg/kg/day, the TFs caused only mild side-effects in both male and female rats. This led to the selection of a dose of 500 mg/kg/day for rats as the no visible adverse effect level (NOAEL) for *R. laevigata.*

Li and Feng. (1990) conducted the first toxicological evaluation test on the brown pigment found in *R. laevigata.* The Ames test is one used for assessment of acute toxicity and the mouse testicular chromosome test that is a mouse bone marrow micronucleus test for harmful elements such as lead and arsenic were performed. The test results showed that the brown pigment was non-toxic, and all other tests were negative, thus concluding that brown pigment in *R. laevigata* is a safe natural pigment. Pang et al. (2006) investigated the mutagenic effect of *R. laevigata* on mice by performing the bone marrow micronucleus sperm deformation and sperm non-programmed DNA (UDS) tests. The results showed that within the dose range used in the experiments, *R. laevigata* did not cause an increase in the frequency of bone marrow micronuclei and hourly sperm malformations in mice. In addition, no induction effect on male mouse germ cells UDS were observed, indicating that *R. laevigata* caused no genetic damage on mice and that is a safer herbal medicine.

## 9 Quality control

### 9.1 Fructus *Rosa laevigata*

#### 9.1.1 Content determination

The factors affecting the variation in the constituent composition of *Rosa laevigata* Michx. are mainly related to geographical distribution. The polysaccharides and TFs in *R. laevigata* varies greatly from different locations and this can range from 30.5 to 42.7% and from 3.1 to 5.3%, respectively (Shi, 2012). In Fructus *R. laevigata*, the polysaccharides and TFs are in negative correspondence, and in general, the herb containing high polysaccharide contents will have a relatively low TF content (Zhang et al., 2014). In addition, Lin and Zhou. (2009) determined the tannin content in the fruits of *R. laevigata* according to the method of the *Chinese Pharmacopoeia* (I) 2005 edition and concluded that the concentration ranged from 10.20 to 29.76%. Their experiments also showed that the tannin content in *R. laevigata* also varies according to the geographical environment. Xu et al. (2002) determined the content of triterpenoid acids in *R. laevigata* from different origins by HPLC, and they showed that the levels of triterpenoid organic acids in the herbs from different origins differed greatly.

National Pharmacopoeia Committee, 2020 edition stipulates that the polysaccharides obtained from *R. laevigata* should not be less than 25.0% with respect to the anhydrous glucose (C_6_H_12_O_6_) content.

#### 9.1.2 Inspection

National Pharmacopoeia Committee, 2020 edition stipulates that the moisture content of *R. laevigata* should not exceed 18.0% and the total ash content should not exceed 5.0%.

#### 9.1.3 Thermogravimetric analysis

By conducting thermos-gravimetric analysis (TGA) studies on four different parts of *R. laevigata* including the roots, stems, pulp and seeds, Zhu et al. (2009) analyzed the characteristic parameters of TGA pyrolysis of different parts of the plant. They also derived the pattern of influence of the rate of temperature rise and species characteristics on the pyrolysis of *R. laevigata*. In addition, their study also showed the ease of decomposition (from easy to difficult) of four parts: seeds, pulp, stem and roots.

#### 9.1.4 Odour analysis

Chen et al. (2011) investigated the variation of odour fingerprints of *R. laevigata* at different time-periods, and then conducted principal component analysis (PCA) and discriminant factor analysis (DFA) on the odour response values measured by using electronic nose (EN) sensors. They also performed statistical quality control (SQC) analysis. The experimental results showed that the combined use of EN, PCA and DFA for odour analysis can be used for quality control of *R. laevigata*.

### 9.2 Radix *Rosa laevigata*

#### 9.2.1 Content determination

In the case of Radix *R. laevigata*, this herb is relatively scarce and is not included in the pharmacopoeia. There is a lack of complete quality standards for it, and therefore there are only a few studies regarding its content determination. Nevertheless, a review of the relevant literature revealed that the amounts of gallic acid and catechin in radix of *R. laevigata* and concoction products from different origins was determined by HPLC. The results showed that the content of gallic acid and catechin in the root of *R. laevigata* from different origins varied significantly, but all the samples contained more catechin than gallic acid (Wei et al., 2021a). In addition, Tan (2012) determined the content of polysaccharides in the roots, stems and fruits of *R. laevigata* from different origins by taking UV spectrophotometry measurement. The results showed that the polysaccharide content of the roots and stems of *R. laevigata* from different origins were lower than the fruits. From this they concluded that there were also differences in the polysaccharide content of different parts of *R. laevigata*.

Wei et al. (2021b) adopted ICP-MS analysis in order to compare the content of metal elements in roots of *R. laevigata* and its associated products from different origins. The results showed that the most abundant macronutrients were Al and Fe, with the concentration of Al reaching 33.9–270 mg/kg in its concoction. Other trace elements found were B, Ba, Mn, Zn and Sr Theis method provides a necessary reference for the development of better quality standards for Radix *R. laevigata*.

#### 9.2.2 Inspection

Since Radix *R. laevigata* is not included in the pharmacopoeia, Su et al. (2012) stipulated through experimental studies, that the moisture content should not exceed 14.0% and the total ash content should not exceed 6.0%, of which the acid insoluble ash content should not exceed 2.0%. The results of this experiment provide a scientific basis for better quality control of Radix *R. laevigata*.

## 10 Extraction and separation processes

Flavonoids and polysaccharides are the main components and active compounds in *Rosa laevigata* Michx., and the optimization studies with respect to their extraction are essential to ensure the efficacy and quality control of this herbal medicine.

### 10.1 Fructus *Rosae levigatae*

#### 10.1.1 Flavonoids

Li et al. (2009) studied the extraction process of flavonoids in *R. laevigata* by using a warm water extraction method. They also used ethanol and acetone for extractions, and then compared the three methods. They found acetone was better than ethanol which was better than warm water for extraction the constituents. The best extraction conditions were obtained by an orthogonal experiment using 60% ethanol, in a 70°C water bath, at a solid-liquid ratio of 1:150. The best extraction conditions were obtained by using the acetone method: acetone concentration 60%, water bath temperature 50°C and a solid-liquid ratio 1:50. Jiang et al. (2015) used the Box-Behnken response surface method to optimize the extraction process of *R. laevigata*, and screened out the best extraction process to be: 60% ethanol concentration, 13:1 material-to-liquid ratio, 2 h extraction time and 2 times extraction.

#### 10.1.2 Polysaccharides

Shang et al. (2018) used the water extraction method to optimize the extraction process of *R. laevigata* formula granules. Their preferred extraction process was eight times the amount of water and two reflux extractions for 2 hours each time. The average extraction rate of polysaccharides from *R. laevigata* was calculated to be 22.17%.

### 10.2 Radix *Rosa laevigata*

Yi et al. (2011) studied the extraction of TFs from the roots of *R. laevigata* by using ethanol. Then they optimized the process conditions for obtaining TFs by using the orthogonal test method. The results showed that the extraction conditions for TFs from the roots of *R. laevigata* were best with 80% ethanol, 50°C extraction, reflux for 2.5 h and a material-liquid ratio of 1:50, and the dissolution rate of TFs was measured as 33.90%. Wu et al. (2011) conducted a comparative study of four different resins to obtain the best macroporous adsorbent resin for the purification of TFs from the roots of *R. laevigata* and optimized this process. The results showed that AB-8 was the best macroporous adsorbent resin for the purification of TFs from the root of *R. laevigata*. The best process for the separation and purification was 20.0 ml of 0.200 mg/ml sample solution with the AB-8 resin as the adsorbent and 50% ethanol as the eluent. The purification rate of TFs from the roots of *R. laevigata* could reach 205.40% under these optimal conditions.

## 11 Future outlook

1) In terms of chemical composition: most of the current research is focused on the study of flavonoids and triterpenes, and there is relatively little work on other active ingredients such as phenols, tannins and polysaccharides. Therefore, there is a need to strengthen the research on the other ingredients in order to improve the progress of the research on the chemical composition of Fructus *R. laevigata* and Radix *R. laevigata.*
2) In terms of the pharmacology: as mentioned above, most of the research today is on flavonoids and triterpenes, so there is a lot of pharmacological studies corresponding to these, while the pharmacological activities of other components are less developed. Moreover, the current pharmacological studies on *R. laevigata* and its roots are mainly focused on its antioxidant and anti-inflammatory activities, and the many other pharmacological effects that are derived from these two activities, such as renal and cardiovascular protection. Therefore, the mechanism of action has to be studied in depth. In addition, the anti-tumour activity, which is widely studied today, is rarely addressed, which leads to the conclusion that research in this area should be strengthened to provide a scientific basis for the development of new drugs.
3) In terms of quality control: While Fructus *R. laevigata* has been included in the *Chinese Pharmacopoeia*, Radix *R. laevigata* is not yet included. Although there are studies on its quality standards, there is still a need to continue to explore its quality standards in depth and to include Radix *R. laevigata* in the pharmacopoeia at an the earliest possible date.
4) In terms of toxicity studies: the toxicity studies on *R. laevigata* are too limited at present and the only study that has produced data is a sub-chronic toxicity study on rats with the TFs of *R. laevigata.* There are no data to support the few other studies and no toxicity studies on other components have so far emerged. Therefore, extensive toxicity studies should be conducted on this aspect to study the toxic effects of the various components of Fructus *R. laevigata* and Radix *R. laevigata* to determine their side-effects, acute toxicity and chronic toxicity to provide a scientific basis for the clinical use of these herbs.
5) In terms of concoction processing: at present, the edible development of *R. laevigata* is limited to pure fruit juice drinks, compound drinks and fruit wine brewing. Further exploration is needed in terms of its food promotion and value innovation.

## 12 Summary

As one of the herbs of medicinal and food origin, *Rosa laevigata* Michx. has strong biological activities of its active chemical components, which has led to its wide use in clinical and daily life. The Chinese National Ministry of Health has rated it as a new food resource and it has now developed into a third generation wild fruit food. Therefore, it is widely used in food ingredients, such as the development of fruit juices, fruit wines and yoghurt. In addition, a brown pigment that can be used as a food additive can also be extracted from *R. laevigata*. Pharmacological studies have shown that *R. laevigata* has the effect of improving the gastrointestinal tract, promoting intestinal peristalsis, increasing the digestion of food, reducing the accumulation of harmful substances in the intestinal tract, playing a role in eliminating persistent stools and also reducing the occurrence of gastrointestinal diseases. It is also used as a raw material in Chinese medicines for the clinical treatment of pelvic inflammatory disease, diabetic cataract, etc. The roots of *R. laevigata* are used as raw materials in San Jin tablets and gynaecological Qian Jin tablets for the treatment of diseases related to gynaecological infections. *R. laevigata* and its roots also have antioxidant, renal protective, immunomodulatory, hypo-lipidemic, anti-inflammatory, antiviral, anti-tumour, cardiovascular protective and antibacterial activities. In addition, it also plays an important role in diabetes mellitus. Chemical composition studies have shown that *R. laevigata* and its roots are rich in triterpenoids, flavonoids and polysaccharides, which are inextricably linked to its diverse pharmacological activities. As more and more scholars continue to study Fructus and Radix Rosae laevigatae in depth, these two herbs have a very promising future in the pharmaceutical, health-care and food industry markets.
