# Supplementary material for: Traditional uses, phytochemical, pharmacology, quality control and modern applications of two important Chinese medicines from Rosa laevigata Michx.: A review
Source: Front Pharmacol. 2022 Oct 6;13:1012265. doi: 10.3389/fphar.2022.1012265 (PMC9582767; doi:10.3389/fphar.2022.1012265)
Supplement: Supplementary file 1 [file Table1.docx]

**Table S1** The use of traditional *R. laevigata* Michx. compounds prescription in China.

| Name of prescription | Prescription | Traditional uses | Prescription sources |
| --- | --- | --- | --- |
| Fructus *R. laevigata* | | |  |
| Capsule Shengjing | *Cervus nippon* Temminck, Cornu Cervi Pantotrichum 46.4g/ *Lycium barbarum*. L, Fructus Lycii 46.4g/ *Panax* ginsengC. A. Mey. Radix et Rhizoma Ginseng 46.4g/ *Cordyceps sinensis* (Berk.) Sacc. Cordyceps 46.4g/ *Cuscuta chinensis* Lam. Semen Cuscutae 46.4g/ *Astragalus complanatus* R. Brown. Semen Astragali Complanati 46.4g/ *Epimedium brevicornu* Maxim. Herba Epimedii 46.4g/ *Polygonatum sibiricum* Red. Rhizoma Polygonati 46.4g/ *Polygonum multiflorum* Thunb. Radix Polygoni Multiflori 92.8g/ *Morus alba* L. Fructus Mori 46.4g/ *Psoralea corylifolia* L. Fructus Psoraleae 46.4g/ *Drynaria fortunei* (Kunze)J. Sm. Rhizoma Drynariae 92.8g/ *Curculigo orchioides* Gaertn. Rhizoma Curculiginis 46.4g/ *R. laevigata* Michx.Fructus *Rosae Laevigatae* 46.4g/ *Rubus chingii* Hu, Fructus Rubi 46.4g/ *Eucommia ulmoides* Oliv. Cortex Eucommiae 46.4g/ *Sargentodoxa cuneata* (Oliv.) Rehd. Caulis Sargentodoxae 46.4g/ *Verbena oficinalis* L. Herba Verbenae Officinalis 46.4g/ *Ginkgo biloba* L. Folium Ginkgo 92.8g | Treatment of soreness and weakness of the waist and knees, dizziness and tinnitus due to deficiency of kidney yang, fatigue, azoospermia, oligospermia, weak sperm, and non-liquidation of semen in men | Compilation of national Chinese patent medicine standards Department of Nephrology, internal medicine |
| Xiaoer Zhixieling granule | *Panax ginseng* C. A. Mey.Radix(生晒参) /*Coix lacryma-jobi* L.var.*ma-yuen*(Roman.)Stapf,Semen Coicis/*Atractylodes macrocephala* Koidz.Rhizoma Atractylodis Macrocephalae (fried)/*Poria cocos* (Schw.)Wolf,Poria/*Gallus gallus domesticus* Brisson,Endothelium Corneum Gigeriae Galli/*Terminalia chebula*Retz.Fructus Chebulae/*Papaver somniferum* L., Pericarpium papaveris/*R. laevigata* Michx.Fructus *Rosae Laevigatae*/*Euryale ferox* Salisb.Semen Euryales/*Massa Medicata Fermentata,*Medicated Leaven | Treatment of spleen deficiency and dampness, intestinal slippage and prolonged diarrhea | Compilation of nationa Chinese patent medicine standards Pediatric volume of oral cancer |
| CORDYCEPS AURICULARIA  oral solution | *Cordyceps sinensis*(Berk.)Sacc.Cordyceps 0.86g/ *Cuora trifasciata* Bell.Concha Curoa(金钱龟) 1.43g/*Dioscorea Morinda officinalis opposita* Thunb.Rhizoma Dioscoreae 14.28g/*Lycium barbarum* L.Fructus Lycii 14.28g/*Eucommia ulmoides* Oliv.Cortex Eucommiae 14.28g/*Morinda officinalis* How,Radix Morindae Officinalis 14.28g/*Astragalus membranaceus*(Fisch.)Bge.var.*mongholicus*(Bge.)Hsiao,Radix Astragali 28.57g/*Codonopsis pilosula* (Franch.)Radix Codonopsis 28.57g/*Poria cocos* (Schw.)Wolf,Poria 28.57g/*Dimocarpus longan* Lour.Arillus Longan 28.57g/*R. laevigata* Michx.Fructus *Rosae Laevigatae* 42.86g/Chicken 500g/Sucrose 50g/ Monosodium glutamate 0.4g | Treatment of dizziness and dizziness caused by deficiency of qi and blood and kidney essence, mental fatigue, loss of appetite, forgetfulness and insomnia, white face and palpitations, lumbago and weakness | Compilation of national Chinese patent medicine standards Internal medicine blood body fluid volume |
| Shouwu tablet | *Polygonum multiflorum* Thunb.Radix Polygoni Multiflori Praeparata cum Succo Glycines Sotae 360g/*Rehmannia glutinosa* Libosch.Radix Rehmanniae 20g/*Achyranthes bidentata* Bl.Radix Achyranthis Bidentatae 40g/*Morus alba* L.Fructus Mori 182g/*Ligustrum lucidum* Ait.Fructus Ligustri Lucidi 40g/Eclipta prostrata (L.) L.Herba Ecliptae 235g/*Morus alba* L.Folium Mori 40g/*Sesamum indicum* L. Semen Sesami Nigrum 16g/*Cuscuta chinensis* Lam.Semen Cuscutae 80g/*R. laevigata* Michx.Fructus *Rosae Laevigatae* 259g/ *Psoralea corylifolia* Fructus Psoraleae 40g (fried with salt)/*Siegesbeckia orientalis* L.Herba Siegesbeckiae 80g/*Lonicerae japonica* Thunb.Flos Lonicerae Japonicae 20g/Magnesium stearate 1.8g/Starch 15g | Treatment of dizziness, tinnitus, waist and limb numbness, premature graying of the hair due to deficiency of the liver and kidneys | Compilation of national Chinese patent medicine standards Internal medicine blood body fluid volume |
| Xiyangsen Jinqiangui mixture | *Panax quinquefolium* L.Radix Panacis Quinquefolii 1.14g/*Chinemys Reevesii*(Gray)/*Chinemys Reevesii*(Gray),Tortoise 1.43g, /*Polygonum multiflorum* Thunb.Radix Polygoni MultifloriPraeparata cum Succo Glycines Sotae 42.86g/*R. laevigata* Michx.Fructus *Rosae Laevigatae* 13g | Treatment of weakness due to deficiency of kidney qi, mental fatigue, weakness of limbs, shortness of breath and lazy speech, dizziness and dizziness, post-illness deficiency | Compilation of national Chinese patent medicine standards Internal medicine blood body fluid volume |
| Yishen Yangyuan Mixture | *Polygonum multiflorum* Thunb.Radix Polygoni Multiflori 156g/*Polygonatum sibiricum* Red.Rhizoma Polygonati 156g/*R. laevigata* Michx.Fructus *Rosae Laevigatae* 364g/*Angelica sinensis* (Oliv.)Diels Radix Angelicae Sinensis 8.3g/*Cibotium barometz*(L.)J.Sm.Rhizoma Cibotii 156g/*Cuscuta chinensis* Lam. Semen Cuscutae 10.4g/*Citrus reticulata* Blanco,Pericarpium Citri Reticulatae 6.3g/ *Psoralea corylifolia* L.Fructus Psoraleae 10.4g/ Sucrose 450g/Sodium benzoate 3g/Talc powder 6g | Treatment of liver and kidney deficiency, weakness of spleen qi, withered face, tiredness and poor circulation, soreness of the waist and knees | Compilation of national Chinese patent medicine standards Internal medicine blood body fluid volume |
| Tonglin capsule | *Smilax glabra* Roxb.Rhizoma Smilacis Glabrae 300g/*Allium tuberosum* Rottl.Semen Allii Tuberosl 300g/*Alisma orientalis*(Sam.)Juzep.Rhizoma Alismatis 200g/ *Atractylodes lancea*(Thunb.)DC.Rjizoma Atractylodis 150g/*Clematis armandii* Franch.Caulis Clematidis Armandii 150g/ *Sophora flavescens* Ait.Radix Sophorae Flavescentis 150g/*Rheum palmatum* L.Radix et Rhizoma Rhei 150g/*R. laevigata* Michx. Fructus *Rosae Laevigatae* 150g/*Cervus nippon* Temminck,Cornu Cervi Pantotrichum 50g/*Scolopendra subspinipes mutilans* L.Koch,Scolopendra 45g/*Benincasa hispida*(Thunb.) Cogn.Semen Benincasae 150g | Treatment of kidney deficiency and stagnation | Compilation of national Chinese patent medicine standards Department of surgery and Gynecology |
| Qianliexiao capsule | *R. laevigata* Michx.Fructus *Rosae Laevigatae* 150g/*Cervus nippon* Temminck,Cornu Cervi Pantotrichum 50g/*Atractylodes macrocephala* Koidz.Rhizoma Atractylodis Macrocephalae 30g/*Rheum palmatum* L.Radix et Rhizoma Rhei 150g/*Polygonum cuspidatum* Sieb. Et Zucc.Rhizoma et Radix Polygoni Cuspidati 150g/*Smilax glabra* Roxb.Rhizoma Smilacis Glabrae 300g/*Sophora flavescens* Ait.Radix Sophorae Flavescentis 150g/*Alisma orientalis*(Sam.)Juzep.Rhizoma Alismatis 200g/*Clematis armandii* Franch.Caulis Clematidis Armandii 150g/*Coix lacryma-jobi* L.var.*ma-yuen*(Roman.)Stapf,Semen Coicis 150g/*Scolopendra subspinipes mutilans* L.Koch,Scolopendra 30g | Treatment of prostatitis | Compilation of national Chinese patent medicine standards Department of surgery and Gynecology |
| Baogong Zhixue Granule | *Ostrea rivilaris* Gould, Concha Ostreae(calcined) 667g/*Paeonia lactiflora* Pall.Radix Paeoniae Alba 333g/*Platycladus orientalis*（L.）Franco, Platycladi Cacumen Carbonisatum 400g /*Rehmannia glutinosa* Libosch.Radix Rehmanniae 333g/*R. laevigata* Michx.Fructus *Rosae Laevigatae*400g/*Bupleurum chinense* DC.Radix Bupleuri (vinegar) 167g/*Panax notoginseng*(Burk.)F.H.Chen,Radix et Rhizoma Notoginseng 67g/*Agrimonia pilosa* Ledeb.Herba Agrimoniae 400g/*Ailanthus altissima*(Mill.)Wingle,Cortex Ailanthi 333g/*Isatis indigotica* Fort.Folium Isatidis 333g | Treatment of excessive menstruation and prolonged menstruation due to deficiency of the flushing process and Yin deficiency and blood heat | SFDA single page standard |
| Ningxin Bushen pill | *Juglans regia* L.Semen Juglandis (degreased, salted) 241g/*Dipsacus asperoides* C.Y.Cheng et T.M.Ai,Radix Dipsaci (roasted with wine) 241g/*Euryale ferox* Salisb.Semen Euryales (salt) 320g/*Codonopsis pilosula* (Franch.)Radix Codonopsis (rice juice) 1600g/*Fossilia* Ossia Mastodi Os Draconis (Shuifei) 241g/*Ziziphus jujuba* Mill.var.*spinosa*(Bge.)Hu ex H.F.Chou,Semen Ziziphi Spinosae (fried) 241g/*R. laevigata* Michx.Fructus *Rosae Laevigatae* (depilated, nucleated) 241g/*Nelumbo nucifera* Gaertn.Stamen Nelumbo (salted) 320g/*Dipsacus asperoides* C.Y.Cheng et T.M.Ai,Radix Dipsaci(salted) 320g/*Polygonum multiflorum* Thunb.Radix Polygoni Multiflori(bean) 320g/*Lycium barbarum* L.Fructus Lycii 320g/*Poria cocos* (Schw.)Wolf,Poria (fried) 160g/ *Psoralea corylifolia* L.Fructus Psoraleae(salted) 400g/*Rubus chingii* Hu,Fructus Rubi(fried) 160g/*Dioscorea opposita* Thunb.Rhizoma Dioscoreae (fried) 241g, /*Astragalus complanatus* R.Brown. Semen Astragali Complanati (steamed with salt) 320g/*Allium tuberosum* Rottl.Semen Allii Tuberosl (fried) 160g/*Amomum villosum* Lour.Fructus Amomi (fried) 47.5g, /*Cuscuta chinensis* Lam.Semen Cuscutae (salted) 241g/*Nelumbo nucifera* Gaertn. Semen Nelumbinis (fried) 241g/*Bos taurus domesticus* Gmelin (roasted, clam powder fried) 100g | Treatment of kidney deficiency, tinnitus, dizziness, palpitations, night sweats, body fatigue, waist and knee weakness | Drug standards of the Ministry of health Volume 8 of traditional Chinese Medicine prescription preparations |
|  |  |  |  |
| Name of prescription | Prescription | Traditional uses | Prescription sources |
| Shuilu Erwei pill | *Euryale ferox* Salisb.Semen Euryales 500g/*R. laevigata* Michx.Fructus *Rosae Laevigatae* 500g | Treatment of kidney deficiency, spermatorrhea, men's spermatorrhea, women's leucorrhea | Drug standards of the Ministry of health Volume 1 of traditional Chinese medicine prescription preparations |
| Jinjihubu pill | *Cibotium barometz*(L.)J.Sm.Rhizoma Cibotii 1278g/*Millettia speciosa* Champ. Radix Millettiae Speciosae 204g/*Kadsura coccinea* (Lem.)A.C.Smith,Radix Kadsurae Coccineae 1022g/*Drynaria fortunei*(Kunze)J.Sm.Rhizoma Drynariae 205g/*Ziziphus jujuba* Mill. Fructus Jujubae 153g/*Spatholobus suberectus* Dunn,Caulis Spatholobi 767g/*Taxillus sutchuenensis*(DC.)Danser,Herba Taxilli (salt wine system) 256g/*R. laevigata* Michx.Fructus *Rosae Laevigatae*(salt system) 256g/*Flemingia prostrata* Roxb.[Flemingia philippinensis Merr.Et Rolfe;Moghania philippinensis （Merr.et Rolfe）Li;M.prostrata（Roxb.）Wang et Tang],Radix Flemingiae Prostratae 204g | Treatment of numbness of the limbs, soreness of the waist and knees, frequent nocturnal urination | Drug standards of the Ministry of health Volume 1 of traditional Chinese medicine prescription preparations |
| Rosa laevigata Paste | *R. laevigata* Michx.Fructus *Rosae Laevigatae* 1000g | Treatment of spermatorrhea, enuresis and excessive leucorrhea due to kidney deficiency | Drug standards of the Ministry of health Volume 1 of traditional Chinese medicine prescription preparations |
| Zhidai tablet | *Patrinia heterophylla* Bunge,Radix Patriniae Heterophyllae 195g/ *Sophora flavescens* Ait.Radix Sophorae Flavescentis 195g/*R. laevigata* Michx.Fructus *Rosae Laevigatae* 260g/*Anemarrhena asphodeloides* Bge.Rhizoma Anemarrhenae(fried with salt) 130g/*Clematis armandii* Franch.Caulis Clematidis Armandii (fried) 130g | Treatment of damp-heat infusion, red, white and yellow belts | Drug standards of the Ministry of health Volume 1 of traditional Chinese medicine prescription preparations |
| Baibu wine | *Cervus nippon* Temminck,Cornu Cervi (pound) 120g/*Anemarrhena asphodeloides* Bge.Rhizoma Anemarrhenae 40g/*Codonopsis pilosula* (Franch.)Radix Codonopsis 30g/*Dioscorea opposita* Thunb.Rhizoma Dioscoreae (fried) 24g/*Astragalus membranaceus*(Fisch.)Bge.var.*mongholicus*(Bge.)Hsiao,Radix Astragali (roasted) 24g/*Poria cocos* (Schw.)Wolf,Poria 24g/*Euryale ferox* Salisb.Semen Euryales 24g/*Lycium barbarum* L.Fructus Lycii 24g/*Cuscuta chinensis* Lam.Semen Cuscutae 24g/*R. laevigata* Michx.Fructus *Rosae Laevigatae* 24g/*Rehmannia glutinosa* Libosch.Radix Rehmanniae 24g/*Achyranthes bidentata* Bl.Radix Achyranthis Bidentatae 18g/*Asparagus cochinchinensis*(Lour.)Merr.Radix Asparagi 24g/*Ophiopogon japonicus* (Thunb.)Ker-Gawl,Radix Ophiopogonis12g/*Broussoneria papyrifera*（L.） Vent. Fructus BrouSieb.et Zucc.Fructus Corni (enucleated) 24g/*Schisandra chinensis* (Turcz.)Baill.Fructus Schisandrae Chinensis 6g/*Dimocarpus longan* Lour.Arillus Longan 6g/*Phellodendron chinense* Schneid.Cortex Phellodendri Chinensis12g/*Cornus officinalis* Sieb. Et Zucc, Fructus Cornus 6g | Treatment of physical weakness, weakness of the waist and knees, dizziness and lightheadedness | Drug standards of the Ministry of health Volume 2 of traditional Chinese medicine prescription preparations |
| Yanshou tablet | *Polygonum multiflorum* Thunb.Radix Polygoni Multiflori 72g/*Siegesbeckia orientalis* L.Herba Siegesbeckiae 16g/*Cuscuta chinensis* Lam.Semen Cuscutae 16g/*Eucommia ulmoides* Oliv.Cortex Eucommiae/*Ligustrum lucidum* Ait.Fructus Ligustri Lucidi 8g/*Morus alba* L.Folium Mori8g/*Lonicerae japonica* Thunb.Flos Lonicerae Japonicae 4g/*Morus alba* L.Fructus Mori 16g*Sesamum indicum* L. Semen Sesami Nigrum 16g/*Rehmannia glutinosa* Libosch.Radix Rehmanniae 4g/*R. laevigata* Michx.Fructus *Rosae Laevigatae* 16g/Eclipta prostrata (L.) L.Herba Ecliptae 16g/Glycine max(Linn.)Merr.Sojae Semen Nigrum 16g/*Achyranthes bidentata* Bl.Radix Achyranthis Bidentatae 8g | Treatment of liver and kidney deficiency, dizziness and dizziness, tinnitus and deafness, sore limbs, lumbar weakness, frequent night urination, premature graying of hair | Drug standards of the Ministry of health Volume 2 of traditional Chinese medicine prescription preparations |
| Shuilu Erwei tablet | *R. laevigata* Michx.Fructus *Rosae Laevigatae* 600g/*Euryale ferox* Salisb.Semen Euryales 600g | Treatment of kidney deficiency, spermatorrhea, men's spermatorrhea, women's leucorrhea | Drug standards of the Ministry of health Volume 5 of traditional Chinese medicine prescription preparations |
| Shenbao syrup | *Cnidium monnieri*(L.)Cuss,Fructus Cnidii 28g/*Cuscuta chinensis* Lam.Semen Cuscutae 66g/*Poria cocos* (Schw.)Wolf,Poria 30g/*Foeniculum vulgare* Mill. Fructus Foeniculi 14.4g/*R. laevigata* Michx.Fructus *Rosae Laevigatae* 94.6/*Angelica sinensis* (Oliv.)Diels Radix Angelicae Sinensis 46.8g/*Polygonum multiflorum* Thunb.Radix Polygoni Multiflori 74.4g/*Rehmannia glutinosa* Libosch.Radix Rehmanniae 94g/*Dioscorea opposita* Thunb.Rhizoma Dioscoreae 46.3g/*Trigonella foenum-graecum* L. Semen Trigonellae 94g/*Cistanche deserticola* Y.C.Ma,Herba Cistanches 47.3g/*Ligusticum chuanxiong* Hort. Rhizoma Chuanxiong 28.3g/ *Psoralea corylifolia* L.Fructus Psoraleae 28.5g/*Panax ginseng* C.A.Mey. Dadix et Rhizoma Ginseng Rubra 20g/*Schisandra chinensis* (Turcz.)Baill.Fructus Schisandrae Chinensis 36g/*Atractylodes macrocephala* Koidz.Rhizoma Atractylodis Macrocephalae 14.2g/*Rubus chingii* Hu,Fructus Rubi 32.9g/*Plantago asiatica* L. Semen Plantaginis 16.5g/*Lycium barbarum* L.Fructus Lycii 66g/*Epimedium brevicornu* Maxim.Herba Epimedii 94.6g/*Astragalus membranaceus*(Fisch.)Bge.var.*mongholicus*(Bge.)Hsiao,Radix Astragali 51.4g/*Glycyrrhiza uralensis* Fisch.Radix et Rhizoma Glycyrrhizae Praeparata cum Melle 14.2g | Treatment of impotence, spermatorrhea, lumbar and leg pain, mental weakness, frequent nocturnal urination, fear of cold, excessive menstruation in women, leucorrhea | Chinese Pharmacopoeia, Volume 1, 2020 Edition |
| Fitness medicinal wine | *Ligustrum lucidum* Ait.Fructus Ligustri Lucidi 29.4g/*Cuscuta chinensis* Lam.Semen Cuscutae 29.4g/*R. laevigata* Michx.Fructus *Rosae Laevigatae* 29.4g/*Cistanche deserticola* Y.C.Ma,Herba Cistanches 29.4g/*Polygonatum sibiricum* Red.Rhizoma Polygonati 29.4g/*Rehmannia glutinosa* Libosch.Radix Rehmanniae 73.5g/*Angelica sinensis* (Oliv.)Diels Radix Angelicae Sinensis 147g/*Cynomorium songaricum* Rupr. Herba Cynomorii 58.8g/*Epimedium brevicornu* Maxim.Herba Epimedii 58.8g/*Polygala tenuifolia* Willd. Radix Polygalae 58.5g/*Glycyrrhiza uralensis* Fisch.Radix et Rhizoma Glycyrrhizae Praeparata cum Melle 14.7g/*Aconitum carmichaeli* Debx. Radix Aconiti Lateralis Praeparata (prepared) 44.1g/*Astragalus membranaceus*(Fisch.)Bge.var.*mongholicus*(Bge.)Hsiao,Radix Astragalis 88.2g/ *Bombyx mori* L. Bombycidae 5.9g/*Gallus domestiaus* 23.5g | Treatment of physical weakness, dizziness, forgetfulness and fatigue, excessive urination at night, anemia and chlorosis, loss of appetite | Drug standards of the Ministry of health Volume 2 of traditional Chinese medicine prescription preparations |
| Jianshen tablet | Eclipta prostrata (L.) L.Herba Ecliptae 72g/*Spatholobus suberectus* Dunn,Caulis Spatholobi 108g/*R. laevigata* Michx.Fructus *Rosae Laevigatae* 72g/mugwort 72g/*Morus alba* L.Fructus Mori 54g/*Cuscuta chinensis* Lam.Semen Cuscutae 36g/*Agrimonia pilosa* Ledeb.Herba Agrimoniae 72g/*Ostrea rivilaris* Gould, Concha Ostreae (calcined) 108g/*Cibotium barometz*(L.)J.Sm.Rhizoma Cibotii (made) 54g/*Ligustrum lucidum* Ait.Fructus Ligustri Lucidi (made) 108g/*Glycyrrhiza uralensis* Fisch.Radix et Rhizoma Glycyrrhizae 18g, Albizzia bark 36g/*Polygonum multiflorum* Thunb.Radix Polygoni Multiflori 54g/*Schisandra chinensis* (Turcz.)Baill.Fructus Schisandrae Chinensis (made) 54g | Treatment of spermatorrhea, sore limbs and weakness | Drug standards of the Ministry of health Volume 2 of traditional Chinese medicine prescription preparations |
| Yishenling granule | *Lycium barbarum* L. Fructus Lycii 20g/*Ligustrum lucidum* Ait.Fructus Ligustri Lucidi 30g/*Aconitum carmichaeli* Debx. Radix Aconiti Lateralis Praeparata (prepared) 2g/*Euryale ferox* Salisb.Semen Euryales (fried) 30g/*Plantago asiatica* L. Semen Plantaginis (fried) 10g/ *Psoralea corylifolia* L.Fructus Psoraleae (fried) 20g/*Rubus chingii* Hu,Fructus Rubi 20g/*Schisandra chinensis* (Turcz.)Baill.Fructus Schisandrae Chinensis 5g/*Morus alba* L.Fructus Mori 20g/*Astragalus complanatus* R.Brown. Semen Astragali Complanati 25g/*Allium tuberosum* Rottl.Semen Allii Tuberosl(fried) 10g, /*Epimedium brevicornu* Maxim.Herba Epimedii 15g/*R. laevigata* Michx.Fructus *Rosae Laevigatae* 20g | Treatment of kidney deficiency impotence, premature ejaculation, spermatorrhea, less sperm, dead sperm | Drug standards of the Ministry of health Volume 2 of traditional Chinese medicine prescription preparations |
| Name of prescription | Prescription | Traditional uses | Prescription sources |
| Zhuang Yao Jian Shen Wan | *Cibotium barometz*(L.)J.Sm.Rhizoma Cibotii (made) 205g/*R. laevigata* Michx.Fructus *Rosae Laevigatae* 60g*Kadsura coccinea* (Lem.)A.C.Smith,Radix Kadsurae Coccineae 115g/*Taxillus chinensis*(DC.)Danser,Herba Taxilli 58g/chicken blood vine 115g/*Flemingia prostrata* Roxb.[Flemingia philippinensis Merr.Et Rolfe;Moghania philippinensis （Merr.et Rolfe）Li;M.prostrata（Roxb.）Wang et Tang],Radix FlemingiaeProstratae 31g/*Millettia speciosa* Champ. Radix Millettiae Speciosae 71g, /*Cuscuta chinensis* Lam.Semen Cuscutae 6g/*Ligustrum lucidum* Ait. Fructus Ligustri Lucidi 6g | Treatment of kidney deficiency and back pain, weak knees, frequent urination, rheumatism and bone pain, neurasthenia | Drug standards of the Ministry of health Volume 3 of traditional Chinese medicine prescription preparations |
| Holly juice | *Ligustrum lucidum* Ait.Fructus Ligustri Lucidi (steamed with wine) 200g/*R. laevigata* Michx.Fructus Rosae Laevigatae 200g/*Ziziphus jujuba* Mill. Fructus Jujubae 200g/*Morus alba* L.Fructus Mori 100g/*Cuscuta chinensis* Lam.Semen Cuscutae 50g/*Polygonatum sibiricum* Red.Rhizoma Polygonati (steamed) 50g/*Cynomorium songaricum* Rupr. Herba Cynomorii 35g/*Rehmannia glutinosa* Libosch.Radix Rehmanniae 30g/*Trigonella foenum-graecum* L. Semen Trigonellae 30g/*Epimedium brevicornu* Maxim.Herba Epimedii 30g/*Schisandra chinensis* (Turcz.)Baill.Fructus Schisandrae Chinensis 15g | Treatment of liver and kidney deficiency, dizziness, frequent urination, soreness and weakness of the waist and knees, neurasthenia | Drug standards of the Ministry of health Volume 4 of traditional Chinese medicine prescription preparations |
| Bushen Qiangshen capsule | *Epimedium brevicornu* Maxim.Herba Epimedii 225g/*R. laevigata* Michx.Fructus *Rosae Laevigatae* 135g/*Cuscuta chinensis* Lam.Semen Cuscutae 135g/*Ligustrum lucidum* Ait. Fructus Ligustri Lucidi (made) 135g/*Cibotium barometz*(L.)J.Sm.Rhizoma Cibotii (made) 135g | Treatment of lumbago and weakness, dizziness and tinnitus, blurred eyes and palpitations | Drug standards of the Ministry of health Volume 4 of traditional Chinese medicine prescription preparations |
| Luwei Bushen pill | *Cervus nippon* Temminck, Cauda Cervi (depilated) 31g/*Paeonia suffruticosa* Andr. Cortex Moutan 15g/*Angelica sinensis* (Oliv.)Diels Radix Angelicae Sinensis(steamed with wine) 230g/*Dioscorea opposita* Thunb.Rhizoma Dioscoreae230g/*Codonopsis pilosula* (Franch.)Radix Codonopsis (steamed) 460g/*Chinemys reevesii*(Gray) Colla Carapacis et Plastri Testudinis 77g/*Cuscuta chinensis* Lam.Semen Cuscutae (steamedwith salt) 123g/*Cynomorium songaricum* Rupr. Herba Cynomorii 153g(steamed) /*Alisma orientalis*(Sam.) Juzep.Rhizoma Alismatis 46g/*Tenodera sinensis* Saussure, Oṏtheca Mantidis (steamed with salt)123g/ *Morinda officinalis* How,Radix Morindae Officinalis (steamed with salt) 153g/*Polygonatum sibiricum* Red.Rhizoma Polygonati (steamed) 123g/*Cordyceps sinensis*(Berk.) Sacc.Cordyceps 77g/*Eucommia ulmoides* Oliv.Cortex Eucommiae (slightly fried)77g/77g Cervus nippon Temminck, Colla Cervi Cornus/*Nelumbo nucifera* Gaertn.Stamen Nelumbo 153g/*Gekko gecko* Linnaeus, Gecko (decapitated and scaly) 5.5g/*Poria cocos* (Schw.)Wolf,Poria 309g/*R. laevigata* Michx.Fructus *Rosae Laevigatae*(enucleated and steamed with salt) 31g/*Lycium barbarum* L.Fructus Lycii 108g, pilose/*Cervus nippon* Temminck,Cornu Cervi Pantotrichum(steamed with wine) 31g/*Drynaria fortunei*(Kunze)J.Sm.Rhizoma Drynariae 46g/*Rubus chingii* Hu,Fructus Rubi (steamed with salt) 77g/*Astragalus membranaceus*(Fisch.)Bge.var.*mongholicus*(Bge.)Hsiao,Radix Astragali 309g | Treating weakness of qi and blood, dizziness and dizziness, forgetfulness, lumbago and leg pain | Drug standards of the Ministry of health Volume 4 of traditional Chinese medicine prescription preparations |
| Anyang Guben ointment | *Lindera aggregata* (Sims)Kosterm. Radix Linderae 36g/*Angelica dahurica* (Fisch.ex Hoffm.)Benth. Et Hook.f.var.formosana(Boiss.)Shan et Yuan, Radix Angelicae Dahuricae 36g/ *Akebia quinata* (Thunb.)Decne. Caulis Akebiae 36g/*Angelica sinensis* (Oliv.)Diels Radix Angelicae Sinensis 36g/ *Paeonia lactiflora* Pall. Radix Paeoniae Rubra 36g/*Rheum palmatum* L.Radix et Rhizoma Rhei 36g/ *Dipsacus asperoides* C.Y.Cheng et T.M.Ai,Radix Dipsaci 36g/*Ailanthus altissima*(Mill.)Wingle,Cortex Ailanthi 36g/*Cyathula officinalis* Kuan, Radix Cyathulae 36g/*Eucommia ulmoides* Oliv.Cortex Eucommiae 36g/*Aconitum carmichaeli* Debx. Radix Aconiti Lateralis Praeparata 36g/*Cynomorium songaricum* Rupr. Herba Cynomorii 36g/*Carthamus tinctorius* L. Flos Carthami 36g/*Morinda officinalis* How,Radix Morindae Officinalis 36g/*Artemisia argyi* Lévl. et Vant. Folium Artemisiae Argyi 72g/*Cyperus rotundus* L. Rhizoma Cyperi 72g/ *Cinnamomum cassia* Presl, Cortex Cinnamomi 72g/*Leonurus japonicus* Houtt. Herba Leonuri 72g/ *R. laevigata* Michx.Fructus *Rosae laevigatae* 18g/*Daemonorops draco* Bl. Sanguis Draconis 14.4g/ *Boswellia carterii* Birdw, Olibanum 7.2g/*Commiphora myrrha* Engler (C.molmol Engler) Myrrha 7.2g/*Acacia catechu* (L.f.)Willd. Catechu 7.2g | Treatment of infertility, dysmenorrhea, menstrual disorders, kidney deficiency | Drug standards of the Ministry of health Volume 6 of traditional Chinese medicine prescription preparations |
| Yilingjing (mixture) | *Polygonum multiflorum* Thunb.Radix Polygoni MultifloriPraeparata cum Succo Glycines Sotae 600g/*R. laevigata* Michx.Fructus *Rosae Laevigatae* 300g/*Morus alba* L. Fructus Mori 300g/*Ligustrum lucidum* Ait Fructus Ligustri lucidi(steamed with wine) 150g, Sigesbeckia orientails Linnaeus (steamed with honey wine) 75g/*Siegesbeckia orientalis* L. Herba Siegesbeckiae (steamed with wine) 75g/*Cuscuta chinensis* Lam.Semen Cuscutae (steamed with wine) 150g | Treatment of dizziness, frequent nighttime urination, tinnitus and palpitations | Drug standards of the Ministry of health Volume 6 of traditional Chinese medicine prescription preparations |
| Tiaojing leucorrhea pill | *Codonopsis pilosula* (Franch.)Radix Codonopsis 52g/Larimichthyus crocea （Richardson), Inglucies Piscis 21g/*Artemisia Argyi* Lévl. Et Vant. Folium Artemisiae Argyi 26g/*Fossilia* Ossis Mastrodi, Os Draconis 26g/*Paeonia suffruticosa* Andr. Cortex Moutan 21g/*polygonatum odoratum* (Mill.)Druce, Rhizoma Polygonati Odorati 26g/*Curculigo orchioides* Gaertn.Rhizoma Curculiginis 26g/*Paeonia lactiflora* Pall.Radix Paeoniae Alba 31g/*Epimedium brevicornu* Maxim.Herba Epimedii 16g/*Ligustrum lucidum* Ait. Fructus Ligustri lucidi/*Euryale ferox* Salisb.Semen Euryales 21g/ *Psoralea corylifolia* L.Fructus Psoraleae 16g/*Alisma orientalis*(Sam.)Juzep.Rhizoma Alismatis 26g/*Polygonum multiflorum* Thunb.Radix Polygoni Multiflori 33g/*Cynomorium songaricum* Rupr. Herba Cynomorii (steamed) 16g/*Taxillus sutchuenensis*(DC.)Danser,Herba Taxilli (salt) 31g/*Chaenomeles speciosa* (Sweet) Nakai, Fructus Chaenomelis 26g/*Dendrobium nobile* Lindl. Caulis Dendrobii 10g/*Cuscuta chinensis* Lam.Semen Cuscutae 31g/ *Equus asinus* L. Colla Corii Asini 26g/*Achyranthes bidentata* Bl.Radix Achyranthis Bidentatae 26g/*Chinemys Reevesii*(Gray),Carapax et Plastrum Testudinis(made of vinegar) 52g/*Ostrea rivilaris* Gould, Concha Ostreae (calcined) 26g/*Angelica sinensis* (Oliv.)Diels Radix Angelicae Sinensis 52g/*R. laevigata* Michx.Fructus *Rosae Laevigatae* 21g/*Poria cocos* (Schw.) Wolf,Poria 39g/*Dioscorea opposita* Thunb.Rhizoma Dioscoreae 31g/*Dipsacus asperoides* C.Y.Cheng et T.M.Ai, Radix Dipsaci 26g/Magnetitum (calcined) 31g/*Aucklandia lappa* Decne. Radix Aucklandiae 21g/*Citrus reticulata* Blanco,Pericarpium Citri Reticulatae 21g/*Rubus chingii* Hu,Fructus Rubi 26g/*Schisandra chinensis* (Turcz.)Baill.Fructus Schisandrae Chinensis 16g/*Glehnia littoralis* Fr. Schmidt ex Miq. Radix Glehniae 20g | Treatment of irregular menstruation, excessive leucorrhea, soreness in the lower back and knees | Drug standards of the Ministry of health Volume 9 of traditional Chinese medicine prescription preparations |
| Morinda officinalis tonic wine | *Morinda officinalis* How,Radix Morindae Officinalis 10g/*Polygonum multiflorum* Thunb.Radix Polygoni Multiflori 8g/*Eucommia ulmoides* Oliv.Cortex Eucommiae 6g/*Cistanche deserticola* Y.C.Ma,Herba Cistanches 4g/*Dipsacus asperoides* C.Y.Cheng et T.M.Ai,Radix Dipsaci 5g/*Curculigo orchioides* Gaertn.Rhizoma Curculiginis 3g/The extract of *R. laevigata* Michx. 10ml/*Epimedium brevicornu* Maxim.Herba Epimedii (leaf) 6g/*Rubus chingii* Hu,Fructus Rubi 5g/*Angelica sinensis* (Oliv.)Diels Radix Angelicae Sinensis 4g/*Codonopsis pilosula* (Franch.)Radix Codonopsis 2g/*Rehmannia glutinosa* Libosch.Radix Rehmanniae 5g/*Lycium barbarum* L.Fructus Lycii 4g/*Glycyrrhiza uralensis* Fisch.Radix et Rhizoma Glycyrrhizae 8g/*Astragalus membranaceus*(Fisch.)Bge.var.*mongholicus*(Bge.)Hsiao,Radix Astragali 3g/*Cibotium barometz* (L.) J.Sm. Rhizoma Cibotii 4g | Treatment of weakness of the waist and knees caused by deficiency of kidney yang and failure of the vital fire | Drug standards of the Ministry of health Volume 17 of traditional Chinese medicine prescription preparations |
| Shouwu Qiangshen tablet | *Polygonum multiflorum* Thunb.Radix Polygoni Multiflori 409g/Eclipta prostrata (L.) L. Herba Ecliptae 273g/*Eucommia ulmoides* Oliv.Folium Eucommiae (fried with salt water) 46g/*Siegesbeckia orientalis* L. Herba Siegesbeckiae 91g/*Achyranthes bidentata* Bl.Radix Achyranthis Bidentatae (fried) 46g/*Morus alba* L.Folium Mori 46g/*Ligustrum lucidum* Ait.Fructus Ligustri Lucidi (steamed) 182g/*Morus alba* L.Fructus Mori 91g/*R. laevigata* Michx.Fructus *Rosae Laevigatae* 68g/*Rubus chingii* Hu,Fructus Rubi 91g | Treatment of liver and kidney weakness, dizziness, soreness of the limbs, weakness of the waist and knees, frequent nocturia | Drug standards of the Ministry of health Volume 7 of traditional Chinese medicine prescription preparations |
| Name of prescription | Prescription | Traditional uses | Prescription sources |
| Mazongshe medicinal wine | *Japalura polygonata*（Hall.）Japalura polygonata (dry) 40g/*Flemingia prostrata* Roxb.[Flemingia philippinensis Merr.Et Rolfe;Moghania philippinensis （Merr.et Rolfe）Li;M.prostrata（Roxb.）Wang et Tang,Radix Flemingiae Prostratae (honey roasted) 40g/*Kadsura coccinea* (Lem.)A.C.Smith,Radix Kadsurae Coccineae 40g/*Eucommia ulmoides* Oliv. Caulis Eucommiae 80g/*Taxillus sutchuenensis*(DC.)Danser,Herba Taxilli 20g/*Bauhinia championi* （Benth.）Benth. Caulis Bauhiniae (honey roasted) 0g/*Spatholobus suberectus* Dunn,Caulis Spatholobi (honey roasted) 80g/*Millettia speciosa* Champ. Radix Millettiae Speciosae (honey roasted) 40g/*Litsea cubeba* (Lour.) Pers. [L.citrata Blume], Semen Litseae 40g/Pterospermum heterophyllum Hance, Radix Pterospermi 20g/*Ardisia gigantifolia* Stapf, Rhizoma Ardisiae Gigantifoliae 20g/*Cibotium barometz* (L.) J.Sm. Rhizoma Cibotii 80g/*R. laevigata* Michx.Fructus *Rosae Laevigatae* 20g | Treatment of lumbar muscle strain, rheumatism of back and leg pain, joint pain | Drug standards of the Ministry of health Volume 8 of traditional Chinese medicine prescription preparations |
| Jinyingzi granule | This product is a granule made of *R. laevigata* Michx.Fructus *Rosae Laevigatae* | Treatment of kidney deficiency | Drug standards of the Ministry of health Volume 8 of traditional Chinese medicine prescription preparations |
| Xiaxiao pill | *Nelumbo nucifera* Gaertn. Semen Nelumbinis 120g/*Dioscorea opposita* Thunb.Rhizoma Dioscoreae 120g (fried with bran), /*Polygonum multiflorum* Thunb.Radix Polygoni Multiflori 120g/ 60g pericarp, 60g keel (calcined) /*R. laevigata* Michx.Fructus *Rosae Laevigatae* 60g/*Polygala tenuifolia* Willd. Radix Polygalae 30g (made with Glycyrrhiza uralensis)/*Poria cocos* (Schw.)Wolf,Poria 120g/*Euryale ferox* Salisb.Semen Euryales 120g/*Nelumbo nucifera* Gaertn.Stamen Nelumbo 60g/*Cuscuta chinensis* Lam.Semen Cuscutae 60g/*Ziziphus jujuba* Mill.var.*spinosa* (Bge.) Hu ex H.F. Chou, Semen Ziziphi Spinosae 60g/ *Terminalia chebula* Retz.Fructus Chebulae (simmered) 60g/*Alisma orientalis*(Sam.) Juzep.Rhizoma Alismatis 45g (fried) | Treatment of seminal emission, seminal clouding, urinary emission and frequent urination | Drug standards of the Ministry of health Volume 9 of traditional Chinese medicine prescription preparations |
| Gujing Bushen pill | *Rehmannia glutinosa* Libosch.Radix Rehmanniae 27g/*Cornus officinalis* Sieb.et Zucc.Fructus Corni 13g/*Lycium barbarum* L.Fructus Lycii 27g/*Schisandra chinensis* (Turcz.)Baill.Fructus Schisandrae Chinensis 6g/*Rubus chingii* Hu,Fructus Rubi 13g/*Acorus tatarinowii* Schott, Rhizoma Acori Tatarinowii 6g/ *Broussonetia papyrifera* (L.)Vent. Fructus Broussonetiae 13g/*Dioscorea opposita* Thunb.Rhizoma Dioscoreae 27g/*R. laevigata* Michx.Fructus *Rosae Laevigatae* 11g/*Poria cocos* (Schw.) Wolf, Poria 18g/*Achyranthes bidentata* Bl.Radix Achyranthis Bidentatae 7g/*Foeniculum vulgare* Mill. Fructus Foeniculi 13g/*Eucommia ulmoides* Oliv.Cortex Eucommiae 27g/*Morinda officinalis* How,Radix Morindae Officinalis 27g/*Cistanche deserticola* Y.C.Ma,Herba Cistanches 27g/*Polygala tenuifolia* Willd. Radix Polygalae 9g/*Cuscuta chinensis* Lam. Semen Cuscutae 18g/*Glycyrrhiza uralensis* Fisch.Radix et Rhizoma Glycyrrhizae 11g | Treatment of spleen and kidney deficiency cold, food loss and fatigue, back pain and body fatigue | Drug standards of the Ministry of health Volume 9 of traditional Chinese medicine prescription preparations |
| Shenyanping granule | *R. laevigata* Michx.Fructus *Rosae Laevigatae* 422g/*Cuscuta chinensis* Lam.Semen Cuscutae 281g/*Dioscorea opposita* Thunb.Rhizoma Dioscoreae 615g/Eclipta prostrata (L.) L.Herba Ecliptae 253g/*Ligustrum lucidum* Ait.Fructus Ligustri Lucidi 253g/*Nelumbo nucifera* Gaertn.Stamen Nelumbo 169g/*Astragalus membranaceus*(Fisch.)Bge.var.*mongholicus*(Bge.)Hsiao,Radix Astragali 281g/*Codonopsis pilosula* (Franch.)Radix Codonopsis 281g/*Atractylodes macrocephala* Koidz.Rhizoma Atractylodis Macrocephalae 169g/*Poria cocos* (Schw.) Wolf, Poria 281g/*Perilla frutescens* (L.) Britt. Folium Perillae 169g/*Cryptotympana pustulata* Fabricius, Periostracum Cicadae 169g/*Leonurus japonicus* Houtt. Herba Leonuri 422g | Treatment of tiredness and fatigue, dizziness and tinnitus, dullness and poor appetite, weakness of the waist and knees, increased nocturia | Drug standards of the Ministry of health Volume 10 of traditional Chinese medicine prescription preparations |
| Jinying Shouwu juice | *Polygonum multiflorum* Thunb.Radix Polygoni Multiflori 100g/*Rehmannia glutinosa* Libosch.Radix Rehmanniae 15g/The Extract of *R. laevigata* Michx. 400ml/*Cibotium barometz*(L.)J.Sm.Rhizoma Cibotii 100g/*Angelica sinensis* (Oliv.)Diels Radix Angelicae Sinensis 5g/*Cuscuta chinensis* Lam.Semen Cuscutae 8g/*Ligusticum chuanxiong* Hort. Rhizoma Chuanxiong 5g | Treatment of lumbar soreness, tinnitus, dizziness, dizziness, tendon impotence, hair loss, gray hair, menstrual disorders due to liver and kidney deficiency, yin deficiency and blood deficiency | Drug standards of the Ministry of health Volume 10 of traditional Chinese medicine prescription preparations |
| Huanyuan Gujing pill | *Rehmannia glutinosa* Libosch.Radix Rehmanniae 180g/*Dioscorea opposita* Thunb.Rhizoma Dioscoreae (fried) 120g/*Paeonia suffruticosa* Andr. Cortex Moutan 120g/*Poria cocos* (Schw.)Wolf,Poria 90g/*Fossilia* Ossia Mastodi, Os Draconis(calcined) 90g/*Euryale ferox* Salisb.Semen Euryales 60g/*Phellodendron chinense* Schneid.Cortex Phellodendri Chinensis(salted) 90g/*R. laevigata* Michx.Fructus *Rosae Laevigatae* 120g/ *Cornus officinais* Sieb. et Zucc. Fructus Corni 180g/*Ostrea rivilaris* Gould, Concha Ostreae (calcined) 60g/*Nelumbo nucifera* Gaertn.Stamen Nelumbo 60g/*Polygala tenuifolia* Willd. Radix Polygalae 60g/*Anemarrhena asphodeloides* Bge.Rhizoma Anemarrhenae (salted) 60g/*Cynomorium songaricum* Rupr. Herba Cynomorii (steamed) 90g | Treatment of deficiency of kidney yin, spermatorrhea, women's deficiency | Drug standards of the Ministry of health Volume 11 of traditional Chinese medicine prescription preparations |
| Shouwubushen wine | *Polygonum multiflorum* Thunb.Radix Polygoni Multiflori 175g/*Paeonia lactiflora* Pall.Radix Paeoniae Alba 15g/*Angelica sinensis* (Oliv.)Diels Radix Angelicae Sinensis 25g/*Dimocarpus longan* Lour. Arillus Longan 40g/*Rehmannia glutinosa* Libosch.Radix Rehmanniae 60g/*Achyranthes bidentata* Bl.Radix Achyranthis Bidentatae 10g/*Astragalus membranaceus*(Fisch.)Bge.var.*mongholicus*(Bge.)Hsiao,Radix Astragali 10g/*Lycium barbarum* L.Fructus Lycii 30g/The Extract of *R. laevigata* Michx. 75ml | Treatment of fatigue, forgetfulness and insomnia, hair loss and graying, dizziness and tinnitus, panic attacks, dreaminess and panic attacks, yellowing of the face, and frequent nocturia due to deficiency of qi and blood, liver and kidney | Drug standards of the Ministry of health Volume 11 of traditional Chinese medicine prescription preparations |
| Jiannaobushen oral liquid | *Panax* *ginseng* C.A.Mey.,Radix et Rhizoma Ginseng 110g/*Cervus nippon* Temminck,Cornu Cervi Pantotrichum 26g/Canis familiaris Linnaeus.Fenis et Testis Canis 52g/ *Cinnamomum cassia* Presl, Cortex Cinnamomi 111g/Aleuritopteris argentea (Gmel.) Fee. Herba Aleuritopteris 44g/*Arctium lappa* L. Fructus Arctii (fried) 66g/*R. laevigata* Michx.Fructus *Rosae Laevigatae* 45g/*Eucommia ulmoides* Oliv.Cortex Eucommiae (charcoal) 133g/*Cyathula officinalis* Kuan, Radix Cyathulae 133g/*Lonicerae japonica* Thunb. Flos Lonicerae Japonicae 96g/*Forsythia suspensa* (Thunb.)Vahl, Fructus Forsythiae 88g/*Cryptotym panapustulata* Fabricius, Periostracum Cicadae 88g/*Dioscorea opposita* Thunb.Rhizoma Dioscoreae 176g/*Polygala tenuifolia* Willd. Radix Polygalae (liquorice) 154g/*Ziziphus jujuba* Mill.var.*spinosa* (Bge.) Hu ex H.F. Chou, Semen Ziziphi Spinosae (fried) 155g/*Amomum villosum* Lour.Fructus Amomi 154g/ *Angelica sinensis* (Oliv.)Diels Radix Angelicae Sinensis 132g, keel (calcined) 129g/*Ostrea rivilaris* Gould, Concha Ostreae 155g/*Poria cocos* (Schw.) Wolf, Poria 309g/*Atractylodes macrocephala* Koidz.Rhizoma Atractylodis Macrocephalae (fried with bran) 155g/*Cinnamomum cassia* Presl, Ramulus Cinnamomi 129g/ *Glycyrrhiza uralensis* Fisch.Radix et Rhizoma Glycyrrhizae 103g/*Paeonia lactiflora* Pall.Radix Paeoniae Alba 129g/*Amomum kravanh* Pierre ex Gagnep. Fructus Rotundus Amomi 128g | Treatment of forgetfulness and insomnia, dizziness, tinnitus and palpitations, lumbar and knee weakness, neurasthenia | Drug standards of the Ministry of health Volume 11 of traditional Chinese medicine prescription preparations |
|  |  |  |  |
|  |  |  |  |
| Name of prescription | Prescription | Traditional uses | Prescription sources |
| Guilu Bushen Oral Liquid | *Cuscuta chinensis* Lam.Semen Cuscutae (fried)/ /*Epimedium brevicornu* Maxim.Herba Epimedii (steamed)/*Dipsacus asperoides* C.Y.Cheng et T.M.Ai,Radix Dipsaci(steamed)/*Cynomorium songaricum* Rupr. Herba Cynomorii(steamed)/*Cibotium barometz*(L.)J.Sm.Rhizoma Cibotii (steamed)/*Ziziphus jujuba* Mill.var.*spinosa* (Bge.) Hu ex H.F. Chou, Semen Ziziphi Spinosae/*Polygonum multiflorum* Thunb.Radix Polygoni MultifloriPraeparata cum Succo Glycines Sotae/*Glycyrrhiza uralensis* Fisch.Radix et Rhizoma Glycyrrhizae Praeparata cum Melle(honey)/*Citrus reticulata* Blanco,Pericarpium Citri Reticulatae (steamed)/ *Cervus Nippon* Temminck, Colla Cervi Cornus (fried)/*Rehmannia glutinosa* Libosch.Radix Rehmanniae/*Chinemys reevesii* (Gray), Colla Carapacis et Plastri Tes (fried)/*R. laevigata* Michx.Fructus *Rosae Laevigatae* (steamed)/*Astragalus membranaceus*(Fisch.)Bge.var.*mongholicus*(Bge.)Hsiao,Radix Astragali (honey roasted)/*Dioscorea opposita* Thunb. Rhizoma Dioscoreae (fried)/*Rubus chingii* Hu,Fructus Rubi (steamed) | Treatment of physical weakness, mental fatigue, lumbar and leg weakness, dizziness, excessive urination at night, forgetfulness and insomnia | Drug standards of the Ministry of health Volume 18 of traditional Chinese medicine prescription preparations |
| Eucommia butiansu pill | *Eucommia ulmoides* Oliv.Cortex Eucommiae (fried with salt water) 31.25g, /*Cuscuta chinensis* Lam.Semen Cuscutae (prepared) 31.25g/*Cistanche deserticola* Y.C.Ma,Herba Cistanches 31.25g/*Polygala tenuifolia* Willd. Radix Polygalae (prepared) 31.25g/*Angelica sinensis* (Oliv.)Diels Radix Angelicae Sinensis(prepared with wine) 31.25g/*Nelumbo nucifera* Gaertn. Semen Nelumbinis 31.25g/*Alisma orientalis*(Sam.)Juzep.Rhizoma Alismatis 31.25g/*Paeonia suffruticosa* Andr. Cortex Moutan 31.25g/*Paeonia lactiflora* Pall. Radix Paeoniae alba 31.25g,/*Epimedium brevicornu* Maxim.Herba Epimedii 28.125g/ *Astragalus membranaceus* (Fisch.) Bge.var.*mongholicus*(Bge.)Hsiao,Radix Astragali 62.5g/*Rehmannia glutinosa* Libosch.Radix Rehmanniae 62.5g/*Dioscorea opposita* Thunb.Rhizoma Dioscoreae 62.5g/ *Poria cocos* (Schw.)Wolf,Poria 62.5g/*Atractylodes macrocephala* Koidz.Rhizoma Atractylodis Macrocephalae 62.5g/*Citrus reticulata* Blanco,Pericarpium Citri Reticulatae 15.625g/*Amomum villosum* Lour. Fructus Amomi 15.625g/*Ligustrum lucidum* Ait.Fructus Ligustri Lucidi 14.06g/*R. laevigata* Michx.Fructus *Rosae Laevigatae* 14.06g/*Cornus officinalis* Sieb.et Zucc.Fructus Corni 3.125g/*Morinda officinalis* How,Radix Morindae Officinalis 3.125g/*Platycladus orientalis* (L.) Franco, Semen Platycladi 3.125g/ *Codonopsis pilosula* (Franch.)Radix Codonopsis 62.5g/*Lycium barbarum* L.Fructus Lycii 62.5g/*Glycyrrhiza uralensis* Fisch.Radix et Rhizoma Glycyrrhizae 31.25g | Treatment of lumbar spine weakness, excessive urination at night, neurasthenia | Drug standards of the Ministry of health Volume 12 of traditional Chinese medicine prescription preparations |
| Eucommia ulmoides butiansu tablets | *Eucommia ulmoides* Oliv.Cortex Eucommiae (fried with salt water) 31.25g/*Cuscuta chinensis* Lam.Semen Cuscutae (prepared) 31.25g/*Cistanche deserticola* Y.C.Ma,Herba Cistanches 31.25g/*Polygala tenuifolia* Willd. Radix Polygalae (prepared) 31.25g/*Angelica sinensis* (Oliv.)Diels Radix Angelicae Sinensis (prepared with wine) 31.25g/*Nelumbo nucifera* Gaertn. Semen Nelumbinis 31.25g/*Alisma orientalis*(Sam.)Juzep.Rhizoma Alismatis 31.25g/*Paeonia suffruticosa* Andr. Cortex Moutan 31.25g and Paeonia alba 31.25g,/*Epimedium brevicornu* Maxim.Herba Epimedii 28.125g/*Astragalus membranaceus*(Fisch.)Bge.var.*mongholicus*(Bge.)Hsiao,Radix Astragali 62.5g/ *Rehmannia glutinosa* Libosch.Radix Rehmanniae 62.5g/*Dioscorea opposita* Thunb.Rhizoma Dioscoreae 62.5g/*Poria cocos* (Schw.)Wolf,Poria 62.5g/*Atractylodes macrocephala* Koidz.Rhizoma Atractylodis Macrocephalae 62.5g/*Citrus reticulata* Blanco,Pericarpium Citri Reticulatae 15.625g/*Amomum villosum* Lour. Fructus Amomi 15.625g/*Ligustrum lucidum* Ait.Fructus Ligustri Lucidi 14.06g/*R. laevigata* Michx.Fructus *Rosae Laevigatae* 14.06g/*Cornus officinalis* Sieb.et Zucc.Fructus Corni 3.125g/*Morinda officinalis* How,Radix Morindae Officinalis 3.125g/*Platycladus orientalis* (L.) Franco, Semen Platycladi 3.125g/ *Codonopsis pilosula* (Franch.)Radix Codonopsis 62.5g/*Lycium barbarum* L.Fructus Lycii 62.5g/*Glycyrrhiza uralensis* Fisch.Radix et Rhizoma Glycyrrhizae 31.25g | Treatment of lumbar spine weakness, excessive urination at night, neurasthenia | Drug standards of the Ministry of health Volume 12 of traditional Chinese medicine prescription preparations |
| Suoyangbushen capsule | *Cynomorium songaricum* Rupr. Herba Cynomorii 31g/*Curculigo orchioides* Gaertn.Rhizoma Curculiginis 31g/*Morinda officinalis* How,Radix Morindae Officinalis 31g/*Angelica sinensis* (Oliv.)Diels Radix Angelicae Sinensis 31g/*Cnidium monnieri* (L.) Cuss. Fructus Cnidii 31g/*Cistanche deserticola* Y.C.Ma,Herba Cistanches 31g (steamed)/*Allium tuberosum* Rottl. Ex Spreng. Semen Allii Tuberosi 47g/*Schisandra chinensis* (Turcz.)Baill.Fructus Schisandrae Chinensis 20g (steamed)/*Panax ginseng* C.A. Mey. Dadix et Rhizoma Ginseng Rubra 16g/*Bos taurus domesticus* Gmelin 16g/Canis familiaris Linnaeus. Fenis et Testis Canis 16g/*Cervus nippon* Temminck,Cornu Cervi Pantotrichum 10g/*Aconitum carmichaelii* Debx. Aconiti Lateralis Radix Praeparata 10g/*Cinnamomum cassia* Presl, Cortex Cinnamomi 10g/*Foeniculum vulgare* Mill. Fructus Foeniculi 10g/*Actinolite*, Tremolitum (calcined) 10g/*Zanthoxylum bungeanum* Maxim. Pericarpium Zanthoxyli 10g,/*Cuscuta chinensis* Lam.Semen Cuscutae 31g/*Eucommia ulmoides* Oliv.Cortex Eucommiae (salted) 31g/*Astragalus complanatus* R.Br. Semen Astragali Complanati (salted) 31g/*Codenopsisp ilosula* (Franch.) Nannf. Radix Codonopisis (honey roasted) 31g/*Cornus officinalis* Sieb.et Zucc.Fructus Corni (steamed) 31g/*Epimedium brevicornu* Maxim.Herba Epimedii 31g/*Astragalus membranaceus*(Fisch.)Bge. var.*mongholicus*(Bge.)Hsiao,Radix Astragali(honey roasted) 31g/*Dioscorea opposita* Thunb.Rhizoma Dioscoreae 31g/*Rehmannia glutinosa* Libosch.Radix Rehmanniae 31g/ *Psoralea corylifolia* L.Fructus Psoraleae (fried with salt) 20g/*Lycium barbarum* L.Fructus Lycii 20g/*Rubus chingii* Hu,Fructus Rubi 20g/*Polygala tenuifolia* Willd. Radix Polygalae 20g/*Nelumbo nucifera* Gaertn.Stamen Nelumbo 20g/ *R. laevigata* Michx.Fructus *Rosae Laevigatae* 20g/*Alisma orientalis*(Sam.)Juzep.Rhizoma Alismatis 10g/ *Glycyrrhiza uralensis* Fisch. Radix et Rhizoma Glycyrrhizae | Treatment of impotence, spermatorrhea and premature ejaculation caused by kidney yang deficiency or kidney yin deficiency | Drug standards of the Ministry of health Volume 12 of traditional Chinese medicine prescription preparations |
| Shujin Jianyao pill | *Cibotium barometz*(L.)J.Sm.Rhizoma Cibotii 600g/*R. laevigata* Michx.Fructus *Rosae Laevigatae* 192g/*Spatholobus suberedus* Dunn, Caulis Spatholobi 360g/*Flemingia prostrata* Roxb.[Flemingia philippinensisMerr.Et Rolfe;Moghania philippinensis （Merr.et Rolfe）Li;M.prostrata（Roxb.） Waang et Tang],Radix Flemingiae Prostratae 144g/ *Kadsura coccinea*（Lem.）A.C. Smith. Radix Kadsurae 360g/*Millettia speciosa* Champ. Radix Millettiae Speciosae 240g/*Ligustrum lucidum* Ait.Fructus Ligustri Lucidi (steamed) 30g/*Taxillus sutchuenensis*(DC.), Danser,Herba Taxilli (steamed) 180g/*Cuscuta chinensis* Lam.Semen Cuscutae (salt) 30g/ *Corydalis yanhusuo* W.T.Wang, Rhizoma Corydalis (made) 26g/*Zanthoxylum nitidum* (Roxb.) DC. Radix Zanthoxyli 26g/*Boswellia carterii* Birdw. Olibanum (made) 12g/*Commiphora myrrha* Engler (C.molmol Engler) Myrrha (made) 20g | Treatment of back and knee pain | Drug standards of the Ministry of health Volume 12 of traditional Chinese medicine prescription preparations |
| Zhuangyaojianshen tablet | *Cibotium barometz*(L.)J.Sm.Rhizoma Cibotii 555.9g/*Kadsura coccinea*（Lem.）A.C. Smith. Radix Kadsurae 333g, Flemingia prostrata/*Flemingia prostrata* Roxb.[Flemingia philippinensis Merr.Et Rolfe;Moghania philippinensis （Merr.et Rolfe）Li;M.prostrata（Roxb.）Wang et Tang],Radix Flemingiae Prostratae 133.9g/*Taxillus sutchuenensis*(DC.)Danser,Herba Taxilli (steamed) 167.1g/*Spatholobus suberectus* Dunn,Caulis Spatholobi 333g/*R. laevigata* Michx.Fructus *Rosae Laevigatae* 177.8g/*Ligustrum lucidum* Ait.Fructus Ligustri Lucidi (steamed) 28.4g/*Millettia speciosa* Champ. Radix Millettiae Speciosae 221.6g,/ *Cuscuta chinensis* Lam.Semen Cuscutae (salt water) 28.4g | Treatment of kidney deficiency and lumbago, weakness of the knees, neurasthenia, frequent urination, rheumatism and bone pain | Drug standards of the Ministry of health Volume 13 of traditional Chinese medicine prescription preparations |
|  |  |  |  |
|  |  |  |  |
|  |  |  |  |
| Name of prescription | Prescription | Traditional uses | Prescription sources |
| Eucommia ulmoides medicinal wine | *Eucommia ulmoides* Oliv.Cortex Eucommiae 25g, Paeonia Alba 5g, acanthopanax senticosus 15g/*Cibotium barometz*(L.)J.Sm.Rhizoma Cibotii 5g/*Rehmannia glutinosa* Libosch.Radix Rehmanniae 12g, cinnamon 5g/*Codonopsis pilosula* (Franch.)Radix Codonopsis 10g/*Drynaria fortunei*(Kunze)J.Sm.Rhizoma Drynariae 5g/*Atractylodes macrocephala* Koidz.Rhizoma Atractylodis Macrocephalae 10g/*R. laevigata* Michx.Fructus *Rosae Laevigatae* 5g/*Ligustrum lucidum* Ait. Fructus Ligustri Lucidi 10g/*Spatholobus suberectus* Dunn,Caulis Spatholobi 5g /*Epimedium brevicornu* Maxim.Herba Epimedii 10g/*Cyathula officinalis* Kuan, Radix Cyathulae 5g/*Poria cocos* (Schw.) Wolf, Poria 10g/*Angelica sinensis* (Oliv.)Diels Radix Angelicae Sinensis 2g/*Cuscuta chinensis* Lam.Semen Cuscutae 5g | Treatment of liver and kidney deficiency, tendon and bone impotence, wind-cold and damp paralysis | Drug standards of the Ministry of health Volume 13 of traditional Chinese medicine prescription preparations |
| Qirong tablets | *Lycium barbarum* L.Fructus Lycii 70g/*Cistanche deserticola* Y.C.Ma,Herba Cistanches 140g/*Cynomorium songaricum* Rupr. Herba Cynomorii 210g/*Cnidium monnieri*(L.)Cuss,Fructus Cnidii 42g/*Ligustrum lucidum* Ait.Fructus Ligustri Lucidi 56g/*Schisandra chinensis* (Turcz.)Baill.Fructus Schisandrae Chinensis 42g/*R. laevigata* Michx.Fructus *Rosae Laevigatae* 56g/*Epimedium brevicornu* Maxim.Herba Epimedii 98g/*Cuscuta chinensis* Lam. Semen Cuscutae 84g | Treatment of insomnia and forgetfulness caused by kidney deficiency | Drug standards of the Ministry of health Volume 13 of traditional Chinese medicine prescription preparations |
| Morinda officinalis oral liquid | *Morinda officinalis* How,Radix Morindae Officinalis 20g/*Polygonum multiflorum* Thunb.Radix Polygoni Multiflori 16g/*Eucommia ulmoides* Oliv.Cortex Eucommiae 12g/*Cistanche deserticola* Y.C.Ma, Herba Cistanches 8g/*Dipsacus asperoides* C.Y.Cheng et T.M.Ai,Radix Dipsaci 10g/*Curculigo orchioides*  Gaertn.Rhizoma Curculiginis 6g/*R. laevigata* Michx.Fructus *Rosae Laevigatae* 20g/*Epimedium brevicornu* Maxim.Herba Epimedii (leaf) 12g/*Rubus chingii* Hu,Fructus Rubi 10g/*Angelica sinensis* (Oliv.)Diels Radix Angelicae Sinensiss 8g/*Codonopsis pilosula* (Franch.)Radix Codonopsis 4g/*Rehmannia glutinosa* Libosch.Radix Rehmanniae 10g/*Lycium barbarum* L.Fructus Lycii 8g/*Glycyrrhiza uralensis* Fisch.Radix et Rhizoma Glycyrrhizae 16g/*Astragalus membranaceus*(Fisch.)Bge.var.*mongholicus*(Bge.) Hsiao,Radix Astragali 6g /*Cibotium barometz*(L.)J.Sm.Rhizoma Cibotii 16g | Treatment of weakness of the waist and knees caused by deficiency of kidney-yang | Drug standards of the Ministry of health Volume 14 of traditional Chinese medicine prescription preparations |
| Zhuangyaobushen pill | *Ligustrum lucidum* Ait. Fructus Ligustri Lucidi (Wine)24g/*Polygonatum sibiricum* Red.Rhizoma Polygonati 24g/*Rehmannia glutinosa* Libosch.Radix Rehmanniae 36g/*R. laevigata* Michx.Fructus *Rosae Laevigatae* 24g/*Cibotium barometz*(L.)J.Sm.Rhizoma Cibotii/*Polygonum multiflorum* Thunb.Radix Polygoni MultifloriPraeparata cum Succo Glycines Sotae 15g/*Flemingia prostrata* Roxb.[Flemingia philippinensis Merr.Et Rolfe;Moghania philippinensis （Merr.et Rolfe）Li;M.prostrata（Roxb.）Wang et Tang],Radix Flemingiae Prostratae 30g | Treatment of lumbago and weakness, dizziness and tinnitus, blurred eyes and palpitations | Chinese Pharmacopoeia, Volume I, 2020 Edition |
| Buxue Tiaojing tablet | *Spatholobus suberectus* Dunn,Caulis Spatholobi 300g/*Equus asinus* L. Colla Corii Asini (fried with sea clam powder) 18g/Rhodomyrtus tomentosa (Ait.) Hassk. Fructus Rhodomyrti 300g/*Cinnamomum cassia* Presl, Cortex Cinnamomi 15g/*Codonopsis pilosula* (Franch.)Radix Codonopsis 90g/*Artemisia argyi* Lévl. et Vant. Folium Artemisiae Argyi (fried) 150g/*Leonurus japonicus* Houtt. Herba Leonuri (prepared) 210g/*R. laevigata* Michx.Fructus *Rosae Laevigatae* 300g/*Ficus simplicissima* Lour.[F. hirta Vahl var. Palmatiloba（Merr.）Chum], Radix Fici Hirtae 150g/*Cyperus rotundus* L. Rhizoma Cyperi (prepared) 300g/Litsea cubeba (Lour.) Pers. Radix Litseae Cubebae 300g/*Alpinia officinarum* Hance, Rhizoma Alpiniae Officinarum 210g/*Clematis armandii* Franch.Caulis Clematidis Armandii 72g/*Flemingia prostrata* Roxb.[Flemingia philippinensis Merr.Et Rolfe;Moghania philippinensis （Merr.et Rolfe）Li;M.prostrata（Roxb.）Wang et Tang],Radix Flemingiae Prostratae 300g/*Taxillus chinensis*(DC.)Danser,Herba Taxilli 300g/Mallotus apelta (Lour.) Muell. Arg. Folium Malloti Apeltae150g/Capsella bursa-pastoris ( Linnaeus ) Medikus, Herba Capsellae Bursa-pastor 120g/*Glycyrrhiza uralensis* Fisch.Radix et Rhizoma Glycyrrhizae Praeparata cum Melle 30g | Treating anemia in women, impotence, redness and whitening, menstrual pain, menstrual leakage, amenorrhea | Drug standards of the Ministry of health Volume 14 of traditional Chinese medicine prescription preparations |
| Jinjie tablet | *R. laevigata* Michx.Fructus *Rosae Laevigatae* 200g/*Gekko gecko* Linnaeus, Gecko 60g/*Epimedium brevicornu* Maxim.Herba Epimedii 555g/*Allium tuberosum* Rottl. Ex Spreng. Semen Allii Tuberosi 60g/*Cornus officinalis* Sieb.et Zucc.Fructus Corni 250g | For the treatment of loss of libido, impotence, spermatorrhea, premature ejaculation, nocturnal urination, residual urination, excessive leucorrhea, soreness and weakness of the waist and knees caused by kidney-yang deficiency | Drug standards of the Ministry of health Volume 14 of traditional Chinese medicine prescription preparations |
| Jinyingzi syrup | This product is a syrup made from processed *R. laevigata* Michx.Fructus *Rosae Laevigatae* | Treatment of seminal emission and spermatorrhea, diarrhea and dysentery | Drug standards of the Ministry of health Volume 14 of traditional Chinese medicine prescription preparations |
| Powerful fitness capsule | *Spatholobus suberectus* Dunn,Caulis Spatholobi 277g/*Polygonatum sibiricum* Red.Rhizoma Polygonati 55g/*R. laevigata* Michx.Fructus *Rosae Laevigatae*(salt water system) 55g/*Millettia speciosa* Champ. Radix Millettiae Speciosae 249g/*Ligustrum lucidum* Ait.Fructus Ligustri Lucidi (salt water system) 5g/*Gallus domestiaus* 44g,/*Cuscuta chinensis* Lam.Semen Cuscutae (salt water system) 55g/*Glycyrrhiza uralensis* Fisch.Radix et Rhizoma Glycyrrhizae 166g/*Polygala tenuifolia* Willd. Radix Polygalae (Glycyrrhiza uralensis system) 111g/Caryopteris incana (Thunb.) Miq. Herba Caryopteridis Incanae 166g/*Cistanche deserticola* Y.C.Ma,Herba Cistanches (salt water system) 55g/*Kadsura coccinea* (Lem.)A.C.Smith,Radix Kadsurae Coccineae 138g/*Rehmannia glutinosa* Libosch.Radix Rehmanniae 138g /*Epimedium brevicornu* Maxim.Herba Epimedii 111g/*Bombyx mori* L. Bombycidae (fried) 11g | Treatment of liver and kidney deficiency, deficiency of yin and blood, dizziness and dizziness, atrophy, forgetfulness and insomnia, kidney deficiency and lumbago | Drug standards of the Ministry of health Volume 14 of traditional Chinese medicine prescription preparations |
| Gengnianle tablets | *Epimedium brevicornu* Maxim.Herba Epimedii 125g/*Ostrea rivilaris* Gould, Concha Ostreae 125g/*Anemarrhena asphodeloides* Bge.Rhizoma Anemarrhenae 25g/*R. laevigata* Michx.Fructus *Rosae Laevigatae* 42g/*Phellodendron chinense* Schneid.Cortex Phellodendri Chinensis 25g/*Plantago asiatica* L. Semen Plantaginis 62.5g/*Panax* ginseng C.A.Mey.,Radix et Rhizoma Ginseng 25g/*Morus alba* L.Fructus Mori 62.5g/*Angelica sinensis* (Oliv.)Diels Radix Angelicae Sinensis 62.5g/*Juglans regia* L.Semen Juglandis 62.5g/ *Cervus nippon* Temminck,Cornu Cervi Pantotrichum 8g/*Psoralea corylifolia* L.Fructus Psoraleae 62.5g/ *Dipsacus asperoides* C.Y.Cheng et T.M.Ai,Radix Dipsaci 62.5g/*Polygonum multiflorum* Thunb.Radix Polygoni Multiflori 125g/*Paeonia lactiflora* Pall.Radix Paeoniae Alba 62.5g/*Polygonum multiflorum* Thunb.Radix Polygoni Multiflori (prepared) 125g/*Achyranthes bidentata* Bl.Radix Achyranthis Bidentatae 42g/*Glycyrrhiza uralensis* Fisch.Radix et Rhizoma Glycyrrhizae Praeparata cum Melle 25g/*Rehmannia glutinosa* Libosch. Radix Rehmanniae Praeparata 35g | Treatment of restless sleep at night, palpitations, tinnitus, suspiciousness, hot sweating, irritability, back pain before and after menopause | Drug standards of the Ministry of health Volume 15 of traditional Chinese medicine prescription preparations |
| Gan Shen An syrup | *Polygonum multiflorum* Thunb.Radix Polygoni MultifloriPraeparata cum Succo Glycines Sotae 293g/*Sesamum indicum* L. Semen Sesami Nigrum 203g/Eclipta prostrata (L.) L.Herba Ecliptae 122g/*Morus alba* L.Fructus Mori 65g/*R. laevigata* Michx.Fructus *Rosae Laevigatae* 65g,/*Cuscuta chinensis* Lam.Semen Cuscutae 65g/{Xi} grass 65g/*Morus alba* L.Folium Mori 33g/*Achyranthes bidentata* Bl.RadixAchyranthis Bidentatae 33g/*Ligustrum lucidum* Ait. Fructus Ligustri Lucidi 33g/*Eucommia ulmoides* Oliv. Cortex Eucommiae 33g/*Rehmannia glutinosa* Libosch.Radix Rehmanniae 16g/*Lonicerae japonica* Thunb.Flos Lonicerae Japonicae 16g/Glycine max(Linn.)Merr.Sojae Semen Nigrum 16g | Treatment of dizziness, tinnitus, lumbago, premature graying of hair | Drug standards of the Ministry of health Volume 15 of traditional Chinese medicine prescription preparations |
| Name of prescription | Prescription | Traditional uses | Prescription sources |
| Tonic Tablet for Kidney-Reinforcing | *Epimedium brevicornu* Maxim.Herba Epimedii 161g/*Cuscuta chinensis* Lam.Semen Cuscutae 97g/*R. laevigata* Michx.Fructus *Rosae Laevigatae* 97g/*Ligustrum lucidum* Ait. Fructus Ligustri Lucidi 97g/*Cibotium barometz*(L.)J.Sm.Rhizoma Cibotii (hot) 97g | Treatment of lumbago and weakness, dizziness and tinnitus, blurred eyes and palpitations | Drug standards of the Ministry of health Volume 15 of traditional Chinese medicine prescription preparations |
| Shenbao mixture | *Cnidium monnieri*(L.)Cuss,Fructus Cnidii 28g/*Ligusticum chuanxiong* Hort. Rhizoma Chuanxiong 28.3g/*Cuscuta chinensis* Lam.Semen Cuscutae 66g/*Psoralea corylifolia* L.Fructus Psoraleae 28.5g/*Poria cocos* (Schw.)Wolf,Poria 30g/*Panax ginseng* C.A.Mey. Dadix et Rhizoma Ginseng Rubra 20g/*Foeniculum vulgare* Mill. Fructus Foeniculi 14.4g/*Schisandra chinensis* (Turcz.)Baill.Fructus Schisandrae Chinensis 36g/*R. laevigata* Michx.Fructus *Rosae Laevigatae* 94.6g/*Atractylodes macrocephala* Koidz.Rhizoma Atractylodis Macrocephalae 14.2g/*Angelica sinensis* (Oliv.)Diels Radix Angelicae Sinensis 46.8g/*Rubus chingii* Hu,Fructus Rubi 32.9g/ *Polygonum multiflorum* Thunb.Radix Polygoni Multiflori 74.4g/ *Plantago asiatica* L. Semen Plantaginis 16.5g/ *Rehmannia glutinosa* Libosch.Radix Rehmanniae 94g/*Lycium barbarum* L.Fructus Lycii 66g/*Dioscorea opposita* Thunb.Rhizoma Dioscoreae 46.3g/*Epimedium brevicornu* Maxim.Herba Epimedii 94.6g/*Trigonella foenum-graecum* L. Semen Trigonellae 94g/*Astragalus membranaceus*(Fisch.)Bge.var.*mongholicus*(Bge.)Hsiao,Radix Astragali 51.4g/*Cistanche deserticola* Y.C.Ma,Herba Cistanches 47.3g/*Glycyrrhiza uralensis* Fisch.Radix et Rhizoma Glycyrrhizae Praeparata cum Melle 14.2g | Treatment of lumbar and leg pain, mental discomfort, frequent nocturnal urination, fear of cold; women with excessive menstruation, leucorrhoea | Chinese Pharmacopoeia, Volume I, 2020 Edition |
| Jinjihu patch | *Cibotium barometz*(L.)J.Sm.Rhizoma Cibotii 521g/*Millettia speciosa* Champ. Radix Millettiae Speciosae 83g/ *Kadsura coccinea*（Lem.）A.C. Smith. Radix Kadsurae 417g/*Drynaria fortunei*(Kunze)J.Sm.Rhizoma Drynariae 83g/*Ziziphus jujuba* Mill. Fructus Jujubae 63g/*Spatholobus suberectus* Dunn,Caulis Spatholobi 312g/*R. laevigata* Michx.Fructus *Rosae Laevigatae* 104g/*Flemingia prostrata* Roxb.[Flemingia philippinensis Merr.Et Rolfe;Moghania philippinensis （Merr.et Rolfe）Li;M.prostrata（Roxb.）Wang et Tang],Radix Flemingiae Prostratae 83g/*Taxillus sutchuenensis*(DC.)Danser,Herba Taxilli (made of salt and wine) 104g | Treatment of paralysis of the limbs, soreness of the waist and knees, frequent nocturia | Drug standards of the Ministry of health Volume 15 of traditional Chinese medicine prescription preparations |
| Shenrong sanbian pill | *Epimedium brevicornu* Maxim.Herba Epimedii (roasted with mutton oil)/*Psoralea corylifolia* L.Fructus Psoraleae(roasted with salt)/*Actinolite*, Tremolitum (calcined)/*Rubus chingii* Hu,Fructus Rubi/*R. laevigata* Michx.Fructus *Rosae Laevigatae*/*Lycium barbarum* L.Fructus Lycii/*Achyranthes bidentata* Bl.Radix Achyranthis Bidentatae/*Cervus nippon* Temminck,Cornu Cervi Pantotrichum/*Cervus nippon* Temminck, Penis et Testis Cervi/Canis familiaris Linnaeus. Fenis et Tesitis Canis/*Equus asinus* L./  *Cynomorium songaricum* Rupr. Herba Cynomorii/*Allium tuberosum* Rottl.Semen Allii Tuberosl/*Cuscuta*  *chinensis* Lam.Semen Cuscutae/*Dipsacus asperoides* C.Y.Cheng et T.M.Ai,Radix Dipsaci/*Rehmannia glutinosa* Libosch.Radix Rehmanniae/*Rheum palmatum* L.Radix et Rhizoma Rhei(salt)/*Panax* ginseng C.A.Mey.,Radix et Rhizoma Ginseng/*Cinnamomum cassia* Presl, Cortex Cinnamomi/*Aconitum carmichaeli* Debx. Radix Aconiti Lateralis Praeparata (prepared)/*Illicium uerum* Hook.f. Fructus Anisi Stellati/*Eucommia ulmoides* Oliv.Cortex Eucommiae (charcoal)/*Atractylodes macrocephala* Koidz.Rhizoma Atractylodis Macrocephalae(fried)/ *Rehmannia glutinosa* Libosch.Radix Rehmanniae/*Ligusticum chuanxiong* Hort. Rhizoma Chuanxiong/ *Aucklandia lappa* Decne. Radix Aucklandiae | Treatment of kidney-yang deficiency and kidney yin deficiency: impotence and spermatorrhea, dimming of the eyes, mental fatigue, weakness of the waist and knees | Drug standards of the Ministry of health Volume 15 of traditional Chinese medicine prescription preparations |
| Jianshen Zhuangyao pill | *Ligustrum lucidum* Ait.Fructus Ligustri Lucidi (steamed with wine) 75g/*Polygonatum sibiricum* Red.Rhizoma Polygonati 75g/*Cibotium barometz*(L.)J.Sm.Rhizoma Cibotii 75g/*R. laevigata* Michx.Fructus *Rosae Laevigatae* 75g/*Flemingia prostrata* Roxb.[Flemingia philippinensis Merr.Et Rolfe;Moghania philippinensis （Merr.et Rolfe）Li;M.prostrata（Roxb.）Wang et Tang],Radix Flemingiae Prostratae 93.8g/*Polygonum multiflorum* Thunb.Radix Polygoni Multiflori Praeparata cum Succo Glycines Sotae 46.9g/*Rehmannia glutinosa* Libosch.Radix Rehmanniae 112.6g | Treatment of rheumatoid arthritis, neurasthenia | Drug standards of the Ministry of health Volume 15 of traditional Chinese medicine prescription preparations |
| Guilu Bushen capsule | *Cuscuta chinensis* Lam.Semen Cuscutae(fried) 128g/*Epimedium brevicornu* Maxim.Herba Epimedii (steamed) 106g/*Dipsacus asperoides* C.Y.Cheng et T.M.Ai,Radix Dipsaci (steamed) 106g/*Cynomorium songaricum* Rupr. Herba Cynomorii (steamed) 128g/*Cibotium barometz*(L.)J.Sm.Rhizoma Cibotii(steamed) 160g/*Ziziphus jujuba* Mill.var.*spinosa* (Bge.) Hu ex H.F. Chou, Semen Ziziphi Spinosae (fried) 106g /*Polygonum multiflorum* Thunb.Radix Polygoni MultifloriPraeparata cum Succo Glycines Sotae 160g/*Glycyrrhiza uralensis* Fisch.Radix et Rhizoma Glycyrrhizae Praeparata cum Melle 53g/*Citrus reticulata* Blanco,Pericarpium Citri Reticulatae (steamed) 53g/*Cervus Nippon* Temminck, Colla Cervi Cornus (fried) 23g/*Rehmannia glutinosa* Libosch.Radix Rehmanniae 160g/*Chinemys Reevesii*(Gray),Colla Carapacis et Plastri Testudinis(fried) 34g/*R. laevigata* Michx.Fructus *Rosae Laevigatae* (steamed) 128g/*Astragalus membranaceus*(Fisch.)Bge.var.*mongholicus*(Bge.)Hsiao,Radix Astragali(honey) 106g/ *Dioscorea opposita* Thunb.Rhizoma Dioscoreae (fried) 106g/*Rubus chingii* Hu,Fructus Rubi (steamed) 213g | Treatment of physical weakness, mental fatigue, lumbar and leg weakness, dizziness, excessive urination at night, forgetfulness and insomnia | Drug standards of the Ministry of health Volume 17 of traditional Chinese medicine prescription preparations |
| Yishi granule | *Codonopsis pilosula* (Franch.)Radix Codonopsis 230g/*Angelica sinensis* (Oliv.)Diels Radix Angelicae Sinensis 230g/*Schisandra chinensis* (Turcz.)Baill.Fructus Schisandrae Chinensis (steamed) 115g/*Dioscorea opposita* Thunb.Rhizoma Dioscoreae 380g/*Polygonum multiflorum* Thunb.Radix Polygoni Multiflori 230g/*R. laevigata* Michx.Fructus *Rosae Laevigatae* 230g/*Rubus chingii* Hu,Fructus Rubi 380g/*Magnolia officinalis* Rehd.et Wils. Cortex Magnoliae Officinalis (ginger) 230g/*Aucklandia lappa* Decne. Radix Aucklandiae 230g/*Atractylodes macrocephala* Koidz.Rhizoma Atractylodis Macrocephalae (coke) 230g/*Santalum album* L. Lignum Santali (coke) 230g/*Photinia serrulata* Lindl. Folium Photiniae 380g/*Cuscuta chinensis* Lam.Semen Cuscutae 380g/*Massa Medicata Fermentata,*Medicated Leaven (coke) 230g | Treatment of adolescent pseudomyopia and visual fatigue caused by liver and kidney deficiency and deficiency of qi and blood | Drug standards of the Ministry of health Volume 17 of traditional Chinese medicine prescription preparations |
| Cuixian pill | *Nelumbo nucifera* Gaertn.Stamen Nelumbo 40g, continuous interruption 30g/*Allium tuberosum* Rottl.Semen Allii Tuberosl(salt stir-fry) 20g/*Astragalus complanatus* R.Brown. Semen Astragali Complanati (stir-fried) 40g/Schizandra chinensis 20g/*Rubus chingii* Hu,Fructus Rubi (salt stir-frying) 20g/*Polygonum multiflorum* Thunb.Radix Polygoni Multiflori 40g/ *Psoralea corylifolia* L.Fructus Psoraleae (salt stir-frying) 30g/*Juglans regia* L.Semen Juglandis 20g, Poria 20g/Larimichthyus crocea (Richardson) Inglucies Piscis (made) 10g/*Panax* ginseng C.A.Mey.,Radix et Rhizoma Ginseng 10g/*Lycium barbarum* L. Fructus Lycii 40g/*Nelumbo nucifera* Gaertn. Semen Nelumbinis (fried) 30g/*Ostrea rivilaris* Gould, Concha Ostreae (calcined) 30g/*Cervus Nippon* Temminck, Cornu Cervi Pantotrichum 10g/*Euryale ferox* Salisb.Semen Euryales (stir-fried) 40g/*Dioscorea opposita* Thunb.Rhizoma Dioscoreae 30g/*R.laevigata* Michx. Fructus *Rosae Laevigatae* 30g | Treatment of kidney deficiency | Drug standards of the Ministry of health Volume 17 of traditional Chinese medicine prescription preparations |
| Guhanyangshengjing | *Panax* ginseng C. A. Mey., Radix et Rhizoma Ginseng/ *Astragalus membranaceus*(Fisch.)Bge.var.*mongholicus*(Bge.)Hsiao, Radix Astragali(honey roasted)/*R. laevigata* Michx. Fructus *Rosae Laevigatae*/ *Lycium barbarum* L.Fructus Lycii/*Ligustrum lucidum* Ait.Fructus Ligustri Lucidi (prepared)/ *Cuscuta chinensis* Lam. Semen Cuscutae/*Epimedium brevicornu* Maxim. Herba Epimedii/ *Paeonia lactiflora* Pall.Radix Paeoniae Alba/ *Glycyrrhiza uralensis* Fisch.Radix et Rhizoma Glycyrrhizae Praeparata cum Melle(honey)/ *Hordeum vulgare* L. Fructus Hordei Germinatus (fried)/ *Polygonatum sibiricum* Red. Rhizoma Polygonati (prepared)/ *Apis cerana* Fabricius | Treatment of dizziness, palpitation, dizziness, tinnitus, forgetfulness, insomnia, fatigue and weakness caused by deficiency of qi and yin and deficiency of kidney essence | Drug standards of the Ministry of health Volume 18 of traditional Chinese medicine prescription preparations |
| Name of prescription | Prescription | Traditional uses | Prescription sources |
| Zishen Ningshen pill | *Rehmannia glutinosa* Libosch.Radix Rehmanniae/ *Dioscorea opposita* Thunb. Rhizoma Dioscoreae/ *R. laevigata* Michx.Fructus *Rosae Laevigatae*/ *Ziziphus jujuba* Mill.var.*spinosa* (Bge.) Hu ex H. F. Chou, Semen Ziziphi Spinosae (fried)/ *Polygonum multiflorum* Thunb. Caulis Polygoni Multiflori/ *Ligustrum lucidum* Ait. Fructus Ligustri Lucidi / *Cuscuta chinensis* Lam. Semen Cuscutae (prepared)/ *Millettia speciosa* Champ. Radix Millettiae Speciosae/ *Poria cocos* (Schw.) Wolf, Poria/ Hyriopsis cumingii (Lea), Concha Margaritifera/ *Paeonia lactiflora* Pall. Radix Paeoniae Alba(fried)/ *Salvia miltiorrhiza* Bge. Radix et Rhizoma Salviae Miltiorrhizae/ *Polygonum multiflorum* Thunb.Radix Polygoni Multiflori / *Polygonatum sibiricum* Red. Rhizoma Polygonati (prepared)/ *Schisandra chinensis* (Turcz.) Baill. Fructus Schisandrae Chinensis*Ficus simplicissima* Lour. [F. hirta Vahl var. palmatiloba （Merr.）Chum], Radix Fici Hirtae | Treatment of liver and kidney deficiency, dizziness and tinnitus, insomnia and dreaminess, dizziness and forgetfulness, lumbago, neurasthenia | Drug standards of the Ministry of health Volume 18 of traditional Chinese medicine prescription preparations |
| Invincible medicinal wine | *Astragalus membranaceus*(Fisch.)Bge. var. *mongholicus*(Bge.) Hsiao, Radix Astragali Angelica sinensis/ *Rehmannia glutinosa* Libosch.Radix Rehmanniae/ Paeonia lactiflora Pall. Radix Paeoniae Rubra/ *Panax* ginseng C. A. Mey. Radix et Rhizoma Ginseng/ *Atractylodes macrocephala* Koidz. Rhizoma Atractylodis Macrocephalae/ *Cuscuta chinensis* Lam. Semen Cuscutae/ *Ligusticum chuanxiong* Hort. Rhizoma Chuanxiong/ *Eucommia ulmoides* Oliv. Cortex Eucommiae/ *Cinnamomum cassia* Presl, Cortex Cinnamomi/ *Cinnamomum cassia* Presl, Ramulus Cinnamomi/peach kernel/ *Rubus chingii* Hu,Fructus Rubi/ *Ligustrum lucidum* Ait. Fructus Ligustri Lucidi/ *R. laevigata* Michx. Fructus *Rosae Laevigatae*/ *Trigonella foenum-graecum* L. Semen Trigonellae/ *Drynaria fortunei*(Kunze) J. Sm. Rhizoma Drynariae/ *Cistanche deserticola* Y. C. Ma, Herba Cistanches/ dragon's blood/ *Angelica dahurica* (Fisch.ex Hoffm.) Benth. Et Hook. f. var. formosana(Boiss.) Shan et Yuan, Radix Angelicae Dahuricae/ *Lycium barbarum* L.Fructus Lycii/ *Boswellia carterii* Birdw. Olibanum (made)/ *Commiphora myrrha* Engler (C.molmol Engler) Myrrha (made)/ *Elecphas maximus* Linnaeus, Periostracum Elecphas/ Manis pentadactyla Linnaeus, Squama Manis/ *Taxillus sutchuenensis*(DC.) Danser, Herba Taxilli/ *Dipsacus asperoides* C. Y. Cheng et T. M. Ai, Radix Dipsaci/ *Rehmannia glutinosa* Libosch. Radix Rehmanniae/ Asarum heterotropoides/ *Salvia miltiorrhiza* Bge. Radix et Rhizoma Salviae Miltiorrhizae/ *Paeonia suffruticosa* Andr. Cortex Moutan/ *Polygonatum sibiricum* Red. Rhizoma Polygonati (prepared)/ *Pueraria lobata* (Willd.) Ohwi, Radix Puerariae Lobatae/ *Sparganium stoloniferum* Buch.-Ham. Rhizoma Sparganii/ Pheretima aspergillum (E.Perrier), Pheretima/ S*patholobus suberedus* Dunn, Caulis Sppatholobi lamb/ *Chaenomeles speciosa* (Sweet) Nakai, Fructus Chaenomelis/ *Luffa cylindrica* (L.) Roem. Fructus Luffae Retinervus/ *Gentiana macrophylla* Pall. Radix Gentianae Macrophyllae | Treatment of acute and chronic sprains and contusions, shoulder, back and lumbar pain, old age and physical weakness, lumbago and leg pain | Drug standards of the Ministry of health Volume 19 of traditional Chinese medicine prescription preparations |
| Changchun Yishou ointment | *Asparagus cochinchinensis*(Lour.)Merr.Radix Asparagi 100g/*Ophiopogon japonicus* (Thunb.)Ker-Gawl,Radix Ophiopogonis 100g/*Rehmannia glutinosa* Libosch.Radix Rehmanniae 100g/*Dioscorea opposita* Thunb.Rhizoma Dioscoreae 100g/*Achyranthes bidentata* Bl.Radix Achyranthis Bidentatae 100g/*Rehmannia glutinosa* Libosch.Radix Rehmanniae 100g/*Eucommia ulmoides* Oliv.Folium Eucommiae 100g/*Polygonum multiflorum* Thunb.Radix Polygoni Multiflori Praeparata cum Succo Glycines Sotae 100g/*Poria cocos* (Schw.)Wolf,Poria 100g/*Panax* ginseng C.A.Mey.,Radix et Rhizoma Ginseng 50g/*Aucklandia lappa* Decne. Radix Aucklandiae 100g/*Platycladus orientalis* (L.) Franco, Semen Platycladi 100g/*Schisandra chinensis* (Turcz.)Baill.Fructus Schisandrae Chinensis 100g/*Cibotium barometz*(L.)J.Sm.Rhizoma Cibotii 100g/*Zanthoxylum bungeanum* Maxim. Pericarpium Zanthoxyli 50g/ *Alisma orientalis*(Sam.)Juzep.Rhizoma Alismatis 50g/*Acorus tatarinowii* Schott, Rhizoma Acori Tatarinowii 50g/*Polygala tenuifolia* Willd. Radix Polygalae (roasted) 50g /*Cuscuta chinensis* Lam.Semen Cuscutae 200g/ *R. laevigata* Michx.Fructus *Rosae Laevigatae* 200g/*Lycium barbarum* L.Fructus Lycii 75g/*Rubus chingii* Hu, Fructus Rubi 75g/*Lycium chinense* Mill. Cortex Lyciipericarp 75g | Treatment of physical weakness and fatigue, palpitation and insomnia, dizziness, lumbar and knee weakness | Drug standards of the Ministry of health Volume 19 of traditional Chinese medicine prescription preparations |
| Guhan Yangsheng granule | *Panax* ginseng C.A.Mey.,Radix et Rhizoma Ginseng/*Astragalus membranaceus*(Fisch.)Bge.var.*mongholicus*(Bge.)Hsiao,Radix Astragali (honey roasted)/*R. laevigata* Michx. Fructus *Rosae Laevigatae*/*Lycium barbarum* L.Fructus Lycii/*Ligustrum lucidum* Ait.Fructus Ligustri Lucidi/*Cuscuta chinensis* Lam.Semen Cuscutae/ *Epimedium brevicornu* Maxim.Herba Epimedii/*Paeonia lactiflora* Pall.Radix Paeoniae Alba/*Glycyrrhiza uralensis* Fisch.Radix et Rhizoma Glycyrrhizae Praeparata cum Melle (honey)/*Hordeum vulgare* L. Fructus Hordei Germinatus (fried)/*Polygonatum sibiricum* Red.Rhizoma Polygonati(prepared) | Treatment of dizziness and palpitation, dizziness and tinnitus, forgetfulness and insomnia, fatigue and weakness, post-illness | Drug standards of the Ministry of health Volume 19 of traditional Chinese medicine prescription preparations |
| Xiaoer Zhixieling capsule | *Panax* ginseng C.A.Mey.,Radix et Rhizoma Ginseng 9g/*Gallus gallus domesticus* Brisson, Corneum Galli Gigerii (fried) 27g/*Poria cocos* (Schw.)Wolf,Poria 36g/*Terminalia chebula* Retz.Fructus Chebulae(fried) 36g/*Euryale ferox* Salisb.Semen Euryales (fried with bran) 18g/*Coix lacryma-jobi* L.var.*ma-yuen*(Roman.)Stapf,Semen Coicis (fried with bran) 36g/*Papaver somniferum* L.Pericarpium papaveris 18g/*Massa Medicata Fermentata,*Medicated Leaven (fried with bran) 18g/ *Atractylodes macrocephala* Koidz.Rhizoma Atractylodis Macrocephalae (fried with bran) 36g/*R. laevigata* Michx.  Fructus *Rosae Laevigatae* (fried with bran) 18g | Treatment of spleen deficiency and dampness, intestinal slippage and prolonged diarrhea | Drug standards of the Ministry of health Volume 20 of traditional Chinese medicine prescription preparations |
| Yishen Yangyuan mixture | *Polygonum multiflorum* Thunb.Radix Polygoni Multiflori 156g/*Cibotium barometz*(L.)J.Sm.Rhizoma Cibotii 156g/*Polygonatum sibiricum* Red.Rhizoma Polygonati 156g/*Cuscuta chinensis* Lam.Semen Cuscutae 10.4g/*R. laevigata* Michx.Fructus *Rosae Laevigatae* 364g/*Psoralea corylifolia* L.Fructus Psoraleae 10.4g / *Angelica sinensis* (Oliv.)Diels Radix Angelicae Sinensis8.3g/*Citrus reticulata* Blanco,Pericarpium Citri Reticulatae 6.3g | Treating deficiency of liver and kidney, weakness of spleen qi, withered face, tiredness and poor circulation, soreness of waist and knees | Drug standards of the Ministry of health Volume 20 of traditional Chinese medicine prescription preparations |
| Zhuangyaojianshen oral liquid | *Cibotium barometz*(L.)J.Sm.Rhizoma Cibotii/*Taxillus sutchuenensis*(DC.)Danser,Herba Taxilli/*R. laevigata* Michx.Fructus *Rosae Laevigatae*/ *Kadsura coccinea*（Lem.）A.C. Smith. Radix Kadsurae/*Ligustrum lucidum* Ait.Fructus Ligustri Lucidi/*Millettia speciosa* Champ. Radix Millettiae Speciosae/*Flemingia prostrata* Roxb. [Flemingia philippinensis Merr.Et Rolfe;Moghania philippinensis （Merr.et Rolfe）Li;M.prostrata（Roxb.）Wang et Tang],Radix Flemingiae Prostratae/*Spatholobus suberectus* Dunn,Caulis Spatholobi/*Cuscuta chinensis* Lam.Semen Cuscutae (made of salt) | Treatment of kidney deficiency and back pain, rheumatism and bone pain, weakness of the knees, frequent urination | New drug regularization standard 32 |
| Yishenling capsule | *Lycium barbarum* L. Fructus Lycii/*Ligustrum lucidum* Ait.Fructus Ligustri Lucidi/*Aconitum carmichaeli* Debx. Radix Aconiti Lateralis Praeparata(prepared)/*Euryale ferox* Salisb.Semen Euryales (fried)/*Plantago asiatica* L. Semen Plantaginis (fried)/ *Psoralea corylifolia* L.Fructus Psoraleae(fried)/*Rubus chingii* Hu,Fructus Rubi/*Schisandra chinensis* (Turcz.)Baill.Fructus Schisandrae Chinensis/*Morus alba* L.Fructus Mori/ *Astragalus complanatus* R.Brown. Semen Astragali Complanati/*Allium tuberosum* Rottl. Ex Spreng. Semen Allii Tuberosi (fried)/*Epimedium brevicornu* Maxim.Herba Epimedii/*R. laevigata* Michx.Fructus *Rosae Laevigatae* | Treatment of kidney deficiency | New drug regularization standard 34 |
| Cangling Zhixie oral liquid | Lonicerae Japonicae 100g/*Bupleurum chinense* DC.Radix Bupleuri 100g/ *Pueraria lobata* (Willd.) Ohwi, Radix Puerariae Lobatae 120g/*Scutellaria baicalensis* Georgi, Radix Scutellariae 100g / *Verbena officinalis* L. Herba Verbenae 150g/*R. laevigata* Michx.Fructus *Rosae Laevigatae* 150g/*Inula helenium* L. Radix Inulae 60g/*Areca catechu* L. Semen Arecae 30g/*Glycyrrhiza uralensis* Fisch.Radix et Rhizoma Glycyrrhizae 30g | Treatment to restore intestinal digestion and absorption function; anti-diarrhea to strengthen the body's immunity | National Drug Standards |
|  |  |  |  |
|  |  |  |  |
| Name of prescription | Prescription | Traditional uses | Prescription sources |
| Guhan Yangshengjing tablet | *Panax* ginseng C.A.Mey.,Radix et Rhizoma Ginseng/*Astragalus membranaceus*(Fisch.)Bge.var.*mongholicus*(Bge.)Hsiao,Radix Astragali(roasted)/*R. laevigata* Michx.Fructus *Rosae Laevigatae*/*Lycium barbarum* L.Fructus Lycii/*Ligustrum lucidum* Ait.Fructus Ligustri Lucidi/*Cuscuta chinensis* Lam. Semen Cuscutae/*Epimedium brevicornu* Maxim.Herba Epimedii/*Paeonia lactiflora* Pall.Radix Paeoniae Alba/ *Glycyrrhiza uralensis* Fisch. Radix et Rhizoma Glycyrrhizae Preparata cum Melle/*Hordeum vulgare* L. Fructus Hordei Germinatus(fried)/*Polygonatum sibiricum* Red.Rhizoma Polygonati | Treatment of dizziness, palpitations, dizziness, tinnitus, forgetfulness, insomnia, fatigue and weakness caused by deficiency of qi and yin and unstable kidney essence; cerebral arteriosclerosis, coronary heart disease, prostate enlargement, menopausal syndrome, post-illness weakness | Chinese Pharmacopoeia, Volume I, 2020 Edition |
| Guhan yangsheng granule | *Panax* ginseng C.A.Mey.,Radix et Rhizoma Ginseng/*Astragalus membranaceus*(Fisch.)Bge.var.*mongholicus*(Bge.)Hsiao,Radix Astragali(roasted)/*R. laevigata* Michx.Fructus Rosae Laevigatae/*Lycium barbarum* L.Fructus Lycii/*Ligustrum lucidum* Ait.Fructus Ligustri Lucidi (made)/*Cuscuta chinensis* Lam.Semen Cuscutae/*Epimedium brevicornu* Maxim.Herba Epimedii/*Paeonia lactiflora* Pall.Radix Paeoniae Alba/ *Glycyrrhiza uralensis* Fisch. Radix et Rhizoma Glycyrrhizae Preparata cum Melle/*Hordeum vulgare* L. Fructus Hordei Germinatus(fried)/*Polygonatum sibiricum* Red. Rhizoma Polygonati(made) | Treatment of dizziness and palpitation, dizziness and tinnitus, forgetfulness and insomnia, fatigue and weakness, post-illness | Chinese Pharmacopoeia, Volume I, 2010 Edition |
| Guilu Bushen pill | *Cuscuta chinensis* Lam.Semen Cuscutae(Salt) 51g/*Epimedium brevicornu* Maxim.Herba Epimedii 43g/*Dipsacus asperoides* C.Y.Cheng et T.M.Ai,Radix Dipsaci 43g/*Cynomorium songaricum* Rupr. Herba Cynomorii 51g/*Cibotium barometz*(L.)J.Sm.Rhizoma Cibotii 64g/*Ziziphus jujuba* Mill.var.*spinosa* (Bunge) Hu ex H.F. Chou, Semen Ziziphi Spinosae 43g/*Polygonum multiflorum* Thunb.Radix Polygoni Multiflori Praeparata cum Succo Glycines Sotae 64g/*Glycyrrhiza uralensis* Fisch.Radix et Rhizoma Glycyrrhizae Praeparata cum Melle 21g/ *Citrus reticulata* Blanco,Pericarpium Citri Reticulatae 21g/*Cervus Nippon* Temminck, Colla Cervi Cornus 9g/*Rehmannia glutinosa* Libosch.Radix Rehmanniae 64g/*Chinemys Reevesii*(Gray),Colla Carapacis et Plastri Testudinis 13g/*R. laevigata* Michx.Fructus *Rosae Laevigatae* 51g/*Astragalus membranaceus*(Fisch.)Bge.var.*mongholicus*(Bge.)Hsiao, Radix Astragali(roasted) 43g/*Dioscorea opposita* Thunb.Rhizoma Dioscoreae 43g/*Rubus chingii* Hu,Fructus Rubi 85g | Treatment of physical weakness, mental fatigue, lumbar and leg weakness, dizziness, kidney deficiency and coldness, loss of libido, excessive urination at night, forgetfulness and insomnia | Chinese Pharmacopoeia, Volume I, 2020 Edition |
| Gushen Dingchuan pill | *Rehmannia glutinosa* Libosch.Radix Rehmanniae 72g/*Aconitum carmichaeli* Debx. Radix Aconiti Lateralis Praeparata 78g/*Paeonia suffruticosa* Andr. Cortex Moutan 52g/*Achyranthes bidentata* Bl.Radix Achyranthis Bidentatae 104g/*Psoralea corylifolia* L.Fructus Psoraleae(salt) 156g/*Amomum villosum* Lour.Fructus Amomi 42g/*Plantago asiatica* L. Semen Plantaginis 104g/*Poria cocos* (Schw.)Wolf,Poria 104g/*Alpinia oxphylla* Miq. Fructus Alpiniae Oxyphyllae (salt) 52g/ *Cinnamomum cassia* Presl, Cortex Cinnamomi 52g/*Dioscorea opposita* Thunb.Rhizoma Dioscoreae 104g/*Alisma orientalis*(Sam.)Juzep.Rhizoma Alismatis 78g/*R. laevigata* Michx. Fructus *Rosae Laevigatae* 52g | Treatment of chronic bronchitis, emphysema, bronchial asthma, asthma in the elderly with deficiency of spleen and kidney and deficiency of lung and kidney qi | Chinese Pharmacopoeia, Volume I, 2020 Edition |
| Shouwu pill | *Polygonum multiflorum* Thunb.Radix Polygoni Multiflori Praeparata cum Succo Glycines Sotae 360g/*Rehmannia glutinosa* Libosch.Radix Rehmanniae 20g/*Achyranthes bidentata* Bl.Radix Achyranthis Bidentatae 40g/*Morus alba* L.Fructus Mori 182g/*Ligustrum lucidum* Ait. Fructus Ligustri Lucidi (wine) 40g/Eclipta prostrata (L.) L.Herba Ecliptae 235g/*Morus alba* L.Folium Mori 40g*Sesamum indicum* L. Semen Sesami Nigrum 16g/ *Cuscuta chinensis* Lam.Semen Cuscutae 80g/*R. laevigata* Michx.Fructus *Rosae Laevigatae* 259g/*Psoralea corylifolia* L.Fructus Psoraleae(Salt) 40g/*Siegesbeckia orientalis* L.Herba Siegesbeckiae 80g/*Lonicerae japonica* Thunb. Flos Lonicerae Japonicae 20g | Treatment of liver and kidney deficiency, dizziness, tinnitus, waist pain and limb numbness, premature graying of the hair | Chinese Pharmacopoeia, Volume I, 2020 Edition |
| Yishenling granule | *Lycium barbarum* L.Fructus Lycii 200g/*Ligustrum lucidum* Ait. Fructus Ligustri Lucidi 300g/*Aconitum carmichaeli* Debx. Radix Aconiti Lateralis Praeparata 20g/*Euryale ferox* Salisb.Semen Euryales 300g/*Plantago asiatica* L. Semen Plantaginis 100g/*Psoralea corylifolia* L.Fructus Psoraleae 200g/*Rubus chingii* Hu,Fructus Rubi 200g/*Schisandra chinensis* (Turcz.)Baill.Fructus Schisandrae Chinensis 50g/*Morus alba* L.Fructus Mori 200g/*Astragalus complanatus* R.Brown. Semen Astragali Complanati 250g/*Allium tuberosum* Rottl. Ex Spreng. Semen Allii Tuberosi 100g/*Epimedium brevicornu* Maxim.Herba Epimedii 150g/*R. laevigata* Michx.Fructus *Rosae Laevigatae* 200g | Treatment of kidney deficiency impotence, premature ejaculation, spermatorrhea, less sperm, dead sperm | Chinese Pharmacopoeia, Volume I, 2020 Edition |
| Wuzi Jiangzhi capsule | *Cnidium monnieri*(L.)Cuss,Fructus Cnidii/*Epimedium brevicornu* Maxim.Herba Epimedii/*R. laevigata* Michx.Fructus *Rosae Laevigatae*/Trogopterus xanthipes Milne-Edwards, Trogopteri Faeces/*Cuscuta chinensis* Lam.Semen Cuscutae/*Lycium barbarum* L.Fructus Lycii/*Salvia miltiorrhiza* Bge. Radix et Rhizoma Salviae Miltiorrhizae/*Spirodela polyrrhiza* (L.) Schleid, Herba Spirodelae/*Schisandra chinensis* (Turcz.)Baill.Fructus Schisandrae Chinensis/*Paeonia lactiflora* Pall. Radix Paeoniae Rubra/*Acorus tatarinowii* Schott, Rhizoma Acori Tatarinowii/ *Polygonum multiflorum* Thunb.Radix Polygoni Multiflori/*Typha angustifolia* L. Pollen Typhae/*Alisma orientalis*(Sam.)Juzep.Rhizoma Alismatis/*Silybum marianum* (L.) Gaertn, Fructus Silybi | Treatment of lumbar and knee weakness, tinnitus, weakness, shortness of breath, lazy speech, chest tightness, chest tingling, dull or blotchy tongue | 77 volumes of new drug positive ratio standards |
| XiPai Maizi Bizi capsule | *Morus alba* L.Fructus Mori/*R. laevigata* Michx.Fructus *Rosae Laevigatae*/*Euryale ferox* Salisb.Semen Euryales/*Gardenia jasminoides* Ellis, Fructus Gardeniae/*Dioscorea spongiosa* J.Q.XI,M.Mizuno et W.L.Zhao, Rhizoma Dioscoreae Spongiosae | Treatment of prostatitis and prostatic hyperplasia resulting in frequent urination, incomplete draining, soreness of the waist and knees, dizziness, poor sleep and tinnitus, premature ejaculation and dream emission | 77 volumes of new drug positive ratio standards |
| XiPai Maizi Bizi oral liquid | *Morus alba* L.Fructus Mori 90g/*R. laevigata* Michx.Fructus *Rosae Laevigatae* 90g/*Euryale ferox* Salisb.Semen Euryales 50g/*Gardenia jasminoides* Ellis, Fructus Gardeniae 40g/*Dioscorea spongiosa* J.Q.XI,M.Mizuno et W.L.Zhao, Rhizoma Dioscoreae Spongiosae 60g | Treatment of prostatitis and prostatic hyperplasia resulting in frequent urination, incomplete draining, soreness of the waist and knees, dizziness, poor sleep and tinnitus, premature ejaculation and dream emission | Drug standards of the Ministry of health Uygur Medicine sub volume |
| Yishen Yangyuan granule | *Polygonum multiflorum* Thunb.Radix Polygoni Multiflori/*Cibotium barometz*(L.)J.Sm.Rhizoma Cibotii/*R. laevigata* Michx.Fructus *Rosae Laevigatae*/*Psoralea corylifolia* L.Fructus Psoraleae/*Polygonatum sibiricum* Red.Rhizoma Polygonati/*Angelica sinensis* (Oliv.)Diels Radix Angelicae Sinensis/*Cuscuta chinensis* Lam.Semen Cuscutae/*Citrus reticulata* Blanco,Pericarpium Citri Reticulatae | Treating deficiency of liver and kidney, weakness of spleen qi, withered face, tiredness and poor circulation, soreness of waist and knees | 81 volumes of new drug positive ratio standards |
|  |  |  |  |
| Name of prescription | Prescription | Traditional uses | Prescription sources |
| Guhan Yangshengjing oral liquid | *Panax* ginseng C.A.Mey.,Radix et Rhizoma Ginseng/*Astragalus membranaceus*(Fisch.) Bge.var.*mongholicus*(Bge.) Hsiao,Radix Astragali(roasted)/*R. laevigata* Michx.Fructus *Rosae Laevigatae*/*Lycium barbarum* L.Fructus Lycii / *Ligustrum lucidum* Ait.Fructus Ligustri Lucidi/*Cuscuta chinensis* Lam.Semen Cuscutae/*Epimedium brevicornu* Maxim.Herba Epimedii/*Paeonia lactiflora* Pall.Radix Paeoniae Alba/*Glycyrrhiza uralensis* Fisch. Radix et Rhizoma Glycyrrhizae Preparata cum Melle/*Hordeum vulgare* L. Fructus Hordei Germinatus(fried)/*Polygonatum sibiricum* Red. Rhizoma Polygonati | Treatment of dizziness, palpitations, dizziness, tinnitus due to deficiency of qi and yin and deficiency of kidney essence | Chinese Pharmacopoeia, Volume I, 2020 Edition |
| Guhan Yangshengjing granule | *Panax* ginseng C.A.Mey.,Radix et Rhizoma Ginseng/*Astragalus membranaceus*(Fisch.)Bge.var.*mongholicus*(Bge.)Hsiao, Radix Astragali(roasted)/*R. laevigata* Michx.Fructus *Rosae Laevigatae*/*Lycium barbarum* L.Fructus Lycii/*Ligustrum lucidum* Ait.Fructus Ligustri Lucidi/*Cuscuta chinensis* Lam.Semen Cuscutae/ *Epimedium brevicornu* Maxim.Herba Epimedii/*Paeonia lactiflora* Pall.Radix Paeoniae Alba/ *Glycyrrhiza uralensis* Fisch. Radix et Rhizoma Glycyrrhizae Preparata cum Melle/*Hordeum vulgare* L. Fructus Hordei Germinatus(fried)/*Polygonatum sibiricum* Red.Rhizoma Polygonati | Treatment of dizziness and palpitation, dizziness and tinnitus, forgetfulness and insomnia, fatigue and weakness, post-illness | Chinese Pharmacopoeia, Volume I, 2020 Edition |
| Baji capsule | *Cibotium barometz*(L.)J.Sm.Rhizoma Cibotii/*Astragalus membranaceus*(Fisch.)Bge.var.*mongholicus*(Bge.)Hsiao,Radix Astragali/*Glycyrrhiza uralensis* Fisch. Radix et Rhizoma Glycyrrhizae/*Lycium barbarum* L.Fructus Lyciiv / *Codonopsis pilosula* (Franch.)Radix Codonopsis/*Angelica sinensis* (Oliv.)Diels Radix Angelicae Sinensis/*Rubus chingii* Hu,Fructus Rubi/*Epimedium brevicornu* Maxim.Herba Epimedii/*R. laevigata* Michx.Fructus *Rosae Laevigatae*/*Curculigo orchioides* Gaertn.Rhizoma Curculiginis/*Dipsacus asperoides* C.Y.Cheng et T.M.Ai,Radix Dipsaci/*Cistanche deserticola* Y.C.Ma,Herba Cistanches/*Eucommia ulmoides* Oliv.Cortex Eucommiae/*Polygonum multiflorum* Thunb.Radix Polygoni Multiflori/*Morinda officinalis* How,Radix Morindae Officinalis | Treatment of weakness of the waist and knees caused by deficiency of kidney-yang | Registration criteria |
| Qirong capsule | *Cuscuta chinensis* Lam.Semen Cuscutae/*Epimedium brevicronu* Maxim.Herba Epimedii/*R. laevigata* Michx.Fructus *Rosae Laevigatae*/*Schisandra chinensis* (Turcz.)Baill.Fructus Schisandrae Chinensis/*Ligustrum lucidum* Ait.Fructus Ligustri Lucidi/*Cnidium monnieri*(L.)Cuss,Fructus Cnidii/*Cynomorium songaricum* Rupr. Herba Cynomorii/ *Cistanche deserticola* Y.C.Ma,Herba Cistanches/*Lycium barbarum* L. Fructus Lycii | Treatment of neurasthenia, kidney deficiency, sexual dysfunction, insomnia | Registration criteria |
| Shengjing tablet | *Ginkgo biloba* L.Folium Ginkgo/ *Verbena officinalis* L. Herba Verbenae/*Sargentodoxa cuneata*(Oliv.)Rehd.Caulis Sargentodoxae/*Eucommia ulmoides* Oliv.Cortex Eucommiae/*Rubus chingii* Hu,Fructus Rubi / *R. laevigata* Michx.Fructus *Rosae Laevigatae*/*Curculigo orchioides* Gaertn.Rhizoma Curculiginis/*Drynaria fortunei*(Kunze) J.Sm.Rhizoma Drynariae/*Psoralea corylifolia* L.Fructus Psoraleae/*Morus alba* L.Fructus Mori/*Polygonum multiflorum* Thunb. Radix Polygoni Multiflori/ *Polygonatum sibiricum* Red.Rhizoma Polygonati/*Epimedium brevicornu* Maxim.Herba Epimedii/ *Astragalus complanatus* R.Brown. Semen Astragali Complanati /*Cuscuta chinensis* Lam.Semen Cuscutae /*Cordyceps sinensis*(Berk.) Sacc.Cordyceps/*Panax* ginseng C.A.Mey.,Radix et Rhizoma Ginseng/*Lycium barbarum* L.Fructus Lycii/ *Cervus nippon* Temminck,Cornu Cervi Pantotrichum | Treatment of soreness and weakness of the waist and knees, dizziness and tinnitus due to deficiency of kidney-yang, fatigue, azoospermia, oligospermia, weak sperm, and non-liquidation of semen in men | Registration criteria |
| Shenbao capsule | *Cistanche deserticola* Y.C.Ma,Herba Cistanches/*Astragalus membranaceus*(Fisch.)Bge.var.*mongholicus*(Bge.)Hsiao,Radix Astragali / *Trigonella foenum-graecum* L. Semen Trigonellae/*Epimedium brevicornu* Maxim.Herba Epimedii/*Dioscorea opposita* Thunb. Rhizoma Dioscoreae/*Lycium barbarum* L.Fructus Lycii/*Rehmannia glutinosa* Libosch.Radix Rehmanniae/*Plantago asiatica* L. Semen Plantaginis/*Polygonum multiflorum* Thunb.Radix Polygoni Multiflori/*Rubus chingii* Hu,Fructus Rubi/*Angelica sinensis* (Oliv.)Diels Radix Angelicae Sinensis/*Atractylodes macrocephala* Koidz.Rhizoma Atractylodis Macrocephalae/*R. laevigata* Michx.Fructus *Rosae Laevigatae*/*Schisandra chinensis* (Turcz.)Baill.Fructus Schisandrae Chinensis/*Foeniculum vulgare* Mill. Fructus Foeniculi/*Panax ginseng* C.A.Mey. Dadix et Rhizoma Ginseng Rubra/*Poria cocos* (Schw.)Wolf,Poria/*Psoralea corylifolia* L. Fructus Psoraleae/*Cuscuta chinensis* Lam.Semen Cuscutae/*Ligusticum chuanxiong* Hort. Rhizoma Chuanxiong/*Cnidium monnieri*(L.)Cuss,Fructus Cnidii/*Glycyrrhiza uralensis* Fisch. Radix et Rhizoma Glycyrrhizae Preparata cum Melle | Treatment of impotence, spermatorrhea, lumbar and leg pain, mental weakness, frequent nocturnal urination, fear of cold, excessive menstruation and leucorrhea in women | Registration criteria |
| XiPai Maizi Bizi tablet | *Gardenia jasminoides* Ellis, Fructus Gardeniae/*Euryale ferox* Salisb.Semen Euryales/*R. laevigata* Michx.Fructus *Rosae Laevigatae*/*Morus alba* L.Fructus Mori/*Dioscorea spongiosa* J.Q.Xi,M.Mizuno et W.L.Zhao, Rhizoma Dioscoreae Spongiosae | Treatment of prostatitis and prostatic hyperplasia resulting in frequent urination, incomplete draining, soreness of the waist and knees, dizziness, poor sleep and tinnitus, premature ejaculation and dream emission | Registration criteria |
| Yanshou tablet | *Polygonum multiflorum* Thunb.Radix Polygoni Multiflori 0.24g/*Siegesbeckia orientalis* L. Herba Siegesbeckiae 53mg/*Cuscuta chinensis* Lam.Semen Cuscutae 53mg/*Eucommia ulmoides* Oliv.Cortex Eucommiae 27mg/*Ligustrum lucidum* Ait.Fructus Ligustri Lucidi 27mg/*Morus alba* L.Folium Mori 27mg/ *Lonicerae japonica* Thunb. Caulis Lonicerae Japonicae 13mg/*Morus alba* L.Fructus Mori 53mg/*Sesamum indicum* L. Semen Sesami Nigrum 53mg/*Rehmannia glutinosa* Libosch.Radix Rehmanniae 13mg/*R. laevigata* Michx.Fructus *Rosae Laevigatae* 53mg/*Eclipta prostrate* L. Herba Ecliptae 53mg/Glycine max(Linn.)Merr.Sojae Semen Nigrum 53mg/*Achyranthes bidentata* Bl.Radix Achyranthis Bidentatae 27mg (drug content of each tablet) | Treatment of liver and kidney deficiency, dizziness and dizziness, tinnitus and deafness, sore limbs, lumbar weakness, frequent night urination, premature graying of hair | New national proprietary Chinese medicine 2nd Edition |
| Yilingjing mixture | *Polygonum multiflorum* Thunb.Radix Polygoni Multiflori Praeparata cum Succo Glycines Sotae 0.36g/*R. laevigata* Michx.Fructus *Rosae Laevigatae* 0.18g/*Morus alba* L.Fructus Mori 0.18g/*Ligustrum lucidum* Ait.Fructus Ligustri Lucidi (steamed with wine) 91mg/*Siegesbeckia orientalis* L.Herba Siegesbeckiae (steamed with honey wine) 45mg/*Cyathula officinalis* Kuan, Radix Cyathulae (steamed with wine) 45mg/*Cuscuta chinensis* Lam.Semen Cuscutae (steamed with wine) 91mg (drug content per ml) | Treatment of dizziness, frequent nighttime urination, tinnitus and palpitations | New national proprietary Chinese medicine 2nd Edition |
| Zishen Jiannao liquid | *Rubus chingii* Hu,Fructus Rubi/*Cuscuta chinensis* Lam.Semen Cuscutae/*Lycium barbarum* L.Fructus Lycii/*R. laevigata* Michx.Fructus *Rosae Laevigatae*/*Ligustrum lucidum* Ait.Fructus Ligustri Lucidi/*Astragalus membranaceus*(Fisch.)Bge.var.*mongholicus*(Bge.)Hsiao,Radix Astragali/*Salvia miltiorrhiza* Bge. Radix et Rhizoma Salviae Miltiorrhizae/*Paeonia lactiflora* Pall.Radix Paeoniae Alba/*Glycyrrhiza uralensis* Fisch.Radix et Rhizoma Glycyrrhizae Praeparata cum Melle(with honey)/*Polygonum multiflorum* Thunb.Radix Polygoni Multiflori Praeparata cum Succo Glycines Sotae/*Epimedium brevicornu* Maxim. Folium Epimedii/*Cinnamomum cassia* Presl, Cortex Cinnamomi | Treatment of dizziness and dizziness caused by liver and kidney deficiency, forgetfulness and insomnia, soreness and weakness of the waist and knees, frequent nocturnal urination | New national proprietary Chinese medicine 2nd Edition |
|  |  |  |  |
|  |  |  |  |
|  |  |  |  |
| Name of prescription | Prescription | Traditional uses | Prescription sources |
| Western Han health preservingoral liquid (Zishen Jiannao liquid) | *Rubus chingii* Hu,Fructus Rubi 120g/ *Cuscuta chinensis* Lam.Semen Cuscutae 120g/*Lycium* L.Fructus Lycii 120g/ *R. laevigata* Michx.Fructus *Rosae Laevigatae* 120g/ *Ligustrum lucidum* Ait.Fructus Ligustri Lucidi 120g/*A stragalus membranaceus*(Fisch.)Bge.var.*mongholicus*(Bge.)Hsiao,Radix Astragali 150g/ *Salvia miltiorrhiza* Bge. Radix et Rhizoma Salviae Miltiorrhizae 120g/*Paeonia lactiflora* Pall.Radix Paeoniae Alba 120g/ *Glycyrrhiza uralensis* Fisch.Radix et Rhizoma Glycyrrhizae Praeparata cum Melle 50g/*Polygonum multiflorum* Thunb.Radix Polygoni Multiflori 150g /*Epimedium brevicornu* Maxim.Herba Epimedii 240g/*Cinnamomum cassia* Presl, Cortex Cinnamomi 10g | Treatment of dizziness and lightheadedness, forgetfulness and insomnia, lumbar and knee weakness, frequent nocturia | Chinese Pharmacopoeia, Volume I, 2020 Edition |
| Pills for inourishing the brain and kidney | *Panax ginseng* C.A.Mey. Dadix et Rhizoma Ginseng Rubra 30g/*Cervus nippon* Temminck,Cornu Cervi Pantotrichum 7g/Canis familiaris Linnaeus. Fenis Et Tesitis Canis 14g/*Cinnamomum cassia* Presl, Cortex Cinnamomi 30g/Aleuritopteris argentea (Gmel.) Fee. Herba Aleuritopteris 12g/Arctium lappa L. Fructus Arctii (fried) 18g/ *R. laevigata* Michx.Fructus *Rosae Laevigatae* 12g/*Eucommia ulmoides* Oliv. Cortex Eucommiae(fried) 36g/ *Cyathula officinalis* Kuan, Radix Cyathulae 36g/*Lonicerae japonica* Thunb.Flos Lonicerae Japonicae 26g/*Forsythia suspensa* (Thunb.) Vahl, Fructus Forsythiae 24g/*Cryptotym panapustulata* Fabricius, Periostracum Cicadae 24g/*Dioscorea opposita* Thunb.Rhizoma Dioscoreae 48g/*Polygala tenuifolia* Willd. Radix Polygalae (prepared) 42g/*Ziziphus jujuba* Mill.var.*spinosa*(Bge.)Hu ex H.F.Chou,Semen Ziziphi Spinosae(fried) 42g/*Amomum villosum* Lour.Fructus Amomi 42g/ *Angelica sinensis* (Oliv.)Diels Radix Angelicae Sinensis 36g/*Os Draconis*(FossiliaOssiaMastodi) 35g/*Ostrea rivilaris* Gould, Concha Ostreae(calcined) 42g, Poria 84g/ *Atractylodes macrocephala* Koidz.Rhizoma Atractylodis Macrocephalae(fried) 42g/*Cinnamomum cassia* Presl, Ramulus Cinnamomi 35g/*Glycyrrhiza uralensis* Fisch.Radix et Rhizoma Glycyrrhizae 28g/*Amomum kravanh* Pierre ex Gagnep. Fructus Amomi Rotundus 35g/ *Paeonia lactiflora* Pall.Radix Paeoniae Alba (wine)35g | Treatment of amnesia, insomnia, dizziness, tinnitus, palpitations, lumbar and knee weakness, seminal emission; neurasthenia and sexual dysfunction due to deficiency of the spleen and kidneys | Chinese Pharmacopoeia, Volume I, 2020 Edition |
| Radix *R. laevigata* | | |  |
| Sanjin capsule | *R. laevigata* Michx.Radix *Rosae Laevigatae* 2020g/*Smilax china* L. Rhizoma Smilacis Chinae 1010g/*Akebia puinata* (Thunb.)De-cne. Fructus Akebiae Puinatae 1010g/Lygodium japonicum (Thunb.) Sw. Herba Lygodii 606g/*Centella asiatica* (CL.) Urb. Herba Centellae 606g/Talcum powder 25g/Starch 25g/Magnesium stearate 3.5g | Treatment of short urination | Compilation of national Chinese patent medicine standards Department of Nephrology, internal medicin |
| Tiezhuo Qingzhuo pill | *Galium aparine* L. var. tenerum  Gren.et (Godr.) Rebb,Herba Galium 417g/Lespedeza cuneata (Dum. -Cours.) G. Don, Herba Lespedezae Cuneatae 417g/*Dioscorea hypoglauca* Palibin, Rhizoma Dioscore Aehypoglaucae 333g/*Houttuynia cordata* Thunb. Herba Houttuyniae 417g/*Taraxacum mongolicum* Hand.-Mazz. Herba Taraxaci 417g/*Formica fusca* L. (黑蚂蚁) 250g/*Akebia quinata* (Thunb.) Decne. Fructus Akebiae 417g/*Plantago asiatica* L. Semen Plantaginis 250g/*Poria cocos* (Schw.)Wolf,Poria 250g/*Dioscorea opposita* Thunb.Rhizoma Dioscoreae 250g/*Alpinia oxyphylla* Miq. Fructus Alpiniae Oxyphyllae/*Cuscuta chinensis* Lam.Semen Cuscutae 250g/*Astragalus complanatus* R.Brown. Semen Astragali Complanati 25og/*R. laevigata* Michx.Radix *Rosae Laevigatae* 333g/*Polygala tenuifolia* Willd. Radix Polygalae 125g/*Glycyrrhiza uralensis* Fisch. Radix et Rhizoma Glycyrrhizae 50g/Starch 240g | Treatment of chronic prostatitis | Compilation of national Chinese patent medicine standards Department of surgery and Gynecology |
| Guangdong Herbal Tea | *Ilex asprella* (Hook.f.et Arn.)Champ.ex Benth. Radix Ilicis Asprellae 38500g/*Oroxylum indicum* (L.) Vent. Semen Oroxyli 125g/*Lophatherum gracile* Brongn. Herba Lophatheri 2250g/Lygodium japonicum (Thunb.) Sw. Herba Lygodii 13000g/*Polygonum chinense* L. Herba Polygonum 5750g/Vitex negundo L. Herba Viticis Negundo 7375g/*R. laevigata* Michx.Radix *Rosae Laevigatae* 13250g/*Microcos paniculata* L. Folium Microctis 2250g/*Helicteres angustifolia* L. Radix Helicteres Angustifoliae 5375g/*Desmodium styracifolium* (Osb.) Merr. Herba Desmodii Styracifolii 3375g | Treatment of four seasonal colds, fever and sore throat, damp-heat stagnation, dry mouth and yellow urine | Drug standards of the Ministry of health Volume 2 of traditional Chinese medicine prescription preparations |
| Ganhe tea | *Scutellaria baicalensis* Georgi, Radix Scutellari 75g/ *Atractylodes lancea*(Thunb.)DC.Rjizoma Atractylodis 75g/*Paeonia lactiflora* Pall. Radix Paeoniae Rubra 75g/*Glycyrrhiza uralensis* Fisch.Radix et Rhizoma Glycyrrhizae 75g/*Alpinia officinarum* Hance, Rhizoma Alpiniae Officinarum 59g/*Saposhnikovia divaricate* (Turcz.) Schischk. Radix Saposhnikoviae 56g/*Citrus reticulata* Blanco, Citri Reticulatae Pericarpium Viride 56g/*Perilla frutescens* (L.) Britt. Folium Perillae 56g/*Schizonepeta tenuifolia* Briq. Herba Schizonepetae 56g/*Bupleurum chinense* DC.Radix Bupleuri 56g/*Artemisia annua* L. Herba Artemisiae Annuae 56g/ *Ilex cornuta* Lindl.ex Paxt. Folium llicis Latifoliae 38g/Massa Medicata Fermentata (fried) 38g/*Platycodon grandiflorum* (Jacq.) A.DC. Radix Pllatycodonis 38g/*Hordeum vulgare* L. Fructus Hordei Germinatus (fried) 30g/ *Crataegus pinnatifida* Bge. var. Major N.E.Br. Fructus Crataegi (fried) 30g/*Ilex rotunda* Thunb. Cortex Ilicis Rotundae 300g/*Cleistocalyx operculatus* （Roxb.）Merr. et Perry, Flos Cleistocalyx 1500g/*R. laevigata* Michx.Radix *Rosae Laevigatae* 1500g/ Ilex asprella (Hook. et Arn.) Champ. ex Benth.Radix et Caulis Ilicis Asprellae 1500g | Treatment of cold and fever, heat stroke and thirst, prevention of cold and flu | Drug standards of the Ministry of health Volume 9 of traditional Chinese medicine prescription preparations |
| Jinji tablets | 1. *laevigata* Michx.Radix *Rosae Laevigatae*/*Spatholobus suberectus* Dunn,Caulis Spatholobi/*Flemingia prostrata* Roxb.[Flemingia philippinensis Merr.Et Rolfe;Moghania philippinensis Merr.et Rolfe）Li;M.prostrata（Roxb.）Wang et Tang],Radix Flemingiae Prostratae/*Mahonia bealei* (Fort.) Carr. Caulis Mahoniae/*Zabthoxylum nitidum* (Roxb.) DC. Radix Zanthoxyli/*Andrographis paniculata* (Burm.f.) Nees, Herba Andrographis | Treatment of adnexitis caused by damp-heat infiltration | Drug standards of the Ministry of health Volume 18 of traditional Chinese medicine prescription preparations |
| Jinji capsule | *R. laevigata* Michx.Radix *Rosae Laevigatae*/*Spatholobus suberectus* Dunn,Caulis Spatholobi/*Flemingia prostrata* Roxb.[Flemingia philippinensis Merr.Et Rolfe;Moghania philippinensis （Merr.et Rolfe）Li;M.prostrata（Roxb.）Wang et Tang],Radix Flemingiae Prostratae/*Mahonia bealei* (Fort.) Carr. Caulis Mahoniae/*Zabthoxylum nitidum* (Roxb.) DC. Radix Zanthoxyli/*Andrographis paniculata* (Burm.f.) Nees, Herba Andrographis | Treatment of adnexitis, endometritis, pelvic inflammatory disease caused by damp-heat infusion | Drug standards of the Ministry of health Volume 16 of traditional Chinese medicine prescription preparations |
| Jinji granule | *R. laevigata* Michx.Radix *Rosae Laevigatae*/*Spatholobus suberectus* Dunn,Caulis Spatholob/*Flemingia prostrata* Roxb.[Flemingia philippinensis Merr.Et Rolfe;Moghania philippinensis （Merr.et Rolfe）Li;M.prostrata（Roxb.）Wang et Tang],Radix Flemingiae Prostratae/*Mahonia bealei* (Fort.) Carr. Caulis Mahoniae/*Zabthoxylum nitidum* (Roxb.) DC. Radix Zanthoxyli/ *Andrographis paniculata* (Burm.f.) Nees, Herba Andrographis | Treatment of adnexitis caused by damp-heat infiltration | Drug standards of the Ministry of health Volume 16 of traditional Chinese medicine prescription preparations |
| Guangdong herbal tea granules | Ilex asprella (Hook. et Arn.) Champ. ex Benth.Radix et Caulis Ilicis Asprellae 308g/*Helicteres angustifolia* L. Radix Helicteres Angustifoliae 43g/Vitex negundo L. Herba Viticis Negundo 59g/*Lophatherum gracile* Brongn. Herba Lophatheri 18g/*Oroxylum indicum* (L.) Vent. Semen Oroxyli 1g/*Microcos paniculata* L. Folium Microctis 18g/*Polygonum chinense* L. Herba Polygonum 46g/Lygodium japonicum (Thunb.) Sw. Herba Lygodii 104g/*Desmodium styracifolium* (Osb.) Merr. Herba Desmodii Styracifolii 27g/*R. laevigata* Michx.Radix *Rosae Laevigatae* 106g | Treatment of four seasonal colds, fever and sore throat, damp-heat stagnation, dry mouth and yellow urine | Drug standards of the Ministry of health Volume 19 of traditional Chinese medicine prescription preparations |
|  |  |  |  |
|  |  |  |  |
|  |  |  |  |
| Name of prescription | Prescription | Traditional uses | Prescription sources |
| Kuai Ying tea | Ilex asprella (Hook. et Arn.) Champ. ex Benth.Radix et Caulis Ilicis Asprellae 18750g/*R. laevigata* Michx.Radix *Rosae Laevigatae* 9375g/*Schefflera octophylla* (Lour.) Harms [Aralia octophylla Lour. ], Cortex Schefflerae 4688g/*Polygonum chinense* L. Herba Polygonum 6250g/*Ilex rotunda* Thunb. Cortex Ilicis Rotundae 6250g/*Lophatherum gracile* Brongn. Herba Lophatheri 4688g/*Helicteres angustifolia* L. Radix Helicteres Angustifoliae 3125g/ Lygodium japonicum (Thunb.) Sw. Herba Lygodii 6250g/*Desmodium styracifolium* (Osb.) Merr. Herba Desmodii Styracifolii 3125g/ Vitex negundo L. Herba Viticis Negundo 10938g/*Microcos paniculata* L. Folium Microctis 7813g/*Oroxylum indicum* (L.) Vent. Semen Oroxyli 625g/Bai Hua Cha 6250g | Treatment of cold and flu | Drug standards of the Ministry of health Volume 19 of traditional Chinese medicine prescription preparations |
| Sanjin granule | *R. laevigata* Michx.Radix *Rosae Laevigatae*/diamond thorn, sheep opening/Lygodium japonicum (Thunb.) Sw. Herba Lygodii/*Centella asiatica* (CL.) Urb. Herba Centellae | Treatment of short and red urine, dripping and astringent pain | Drug standards of the Ministry of health Volume 20 of traditional Chinese medicine prescription preparations |
| Gynecologic Qianjin tablets | *Flemingia prostrata* Roxb.[Flemingia philippinensis Merr.Et Rolfe;Moghania philippinensis （Merr.et Rolfe）Li;M.prostrata（Roxb.）Wang et Tang],Radix Flemingiae Prostratae/*R. laevigata* Michx.Radix *Rosae Laevigatae*/*Andrographis paniculata* (Burm.f.) Nees, Herba Andrographis/*Mahonia bealei* (Fort.) Carr. Caulis Mahoniae/single-sided needle/*Angelica sinensis* (Oliv.)Diels Radix Angelicae Sinensis/ *Spatholobus suberectus* Dunn,Caulis Spatholobi/*Codonopsis pilosula* (Franch.)Radix Codonopsis | Treatment of chronic pelvic inflammatory disease, endometritis, chronic cervicitis | Chinese Pharmacopoeia, Volume I,  2020 Edition |
| Gynecologic Qianjin capsule | *Flemingia prostrata* Roxb.[Flemingia philippinensis Merr.Et Rolfe;Moghania philippinensis （Merr.et Rolfe）Li;M.prostrata（Roxb.）Wang et Tang],Radix Flemingiae Prostratae/*R. laevigata* Michx.Radix *Rosae Laevigatae*/*Andrographis paniculata* (Burm.f.) Nees, Herba Andrographis/*Mahonia bealei* (Fort.) Carr. Caulis Mahoniae/single-sided needle/*Angelica sinensis* (Oliv.)Diels Radix Angelicae Sinensis/ *Spatholobus suberectus* Dunn,Caulis Spatholobi/*Codonopsis pilosula* (Franch.)Radix Codonopsis | Treatment of chronic pelvic inflammatory disease, endometritis, chronic cervicitis | Chinese Pharmacopoeia, Volume I, 2020 Edition |
| Jinji pill | *Zabthoxylum nitidum* (Roxb.) DC. Radix Zanthoxyli/*Mahonia bealei* (Fort.) Carr. Caulis Mahoniae/*Flemingia prostrata* Roxb.[Flemingia philippinensis Merr.Et Rolfe;Moghania philippinensis （Merr.et Rolfe）Li;M.prostrata（Roxb.）Wang et Tang],Radix Flemingiae Prostratae/*Spatholobus suberectus* Dunn,Caulis Spatholobi/ *R. laevigata* Michx.Radix *Rosae Laevigatae*/*Andrographis paniculata* (Burm.f.) Nees, Herba Andrographis | Treatment of adnexitis, endometritis, pelvic inflammatory disease caused by damp-heat infusion | Registration criteria |
| Sanjin pill | *R. laevigata* Michx.Radix *Rosae Laevigatae* 808g/*Smilax china* L. Rhizoma Smilacis Chinae 404g/*Akebia puinata* (Thunb.)De-cne. Fructus Akebiae Puinatae 404g/Lygodium japonicum (Thunb.) Sw. Herba Lygodii 242.4g/*Centella asiatica* (CL.) Urb. Herba Centellae 242.4g | Treatment of acute and chronic pyelonephritis, cystitis, and urinary tract infections | Chinese Pharmacopoeia, Volume I, 2020 Edition |
